# Exploration of Thiourea-Based Scaffolds for the Construction of Bacterial Ureases Inhibitors

**DOI:** 10.1021/acsomega.3c03702

**Published:** 2023-07-28

**Authors:** Wojciech Tabor, Aikaterini Katsogiannou, Danai Karta, Evgenia Andrianopoulou, Łukasz Berlicki, Stamatia Vassiliou, Agnieszka Grabowiecka

**Affiliations:** † Department of Bioorganic Chemistry, Faculty of Chemistry, Wrocław University of Science and Technology, Wybrzeże Wyspiańskiego 27, 50-370 Wrocław, Poland; ‡ Laboratory of Organic Chemistry, Department of Chemistry, University of Athens, Panepistimiopolis, Zografou, 15771 Athens, Greece

## Abstract

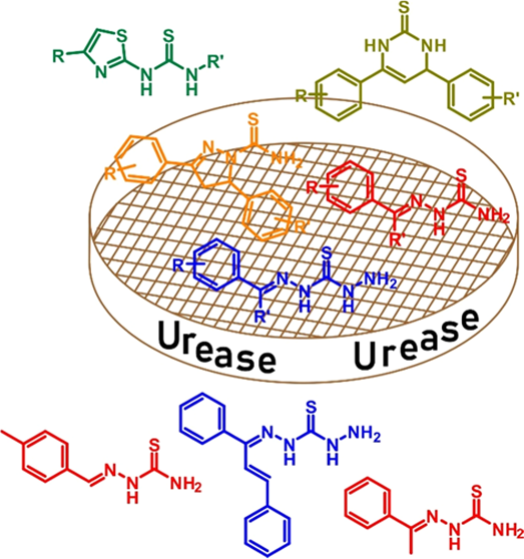

A series of 32 thiourea-based urease inhibitors were synthesized and evaluated against native bacterial enzyme and whole cells of *Sporosarcina pasteurii* and *Proteus mirabilis* strains. The proposed inhibitors represented structurally diverse thiosemicarbazones and thiocarbohydrazones, benzyl-substituted thiazolyl thioureas, 1*H*-pyrazole-1-carbothioamides, and dihydropirimidine-2(1*H*)-thiones. Kinetic characteristics with purified *S. pasteurii* enzyme determined low micromolar inhibitors within each structural group. (*E*)-2-(1-Phenylethylidene)hydrazine-1-carbothioamide **19** (*K*_*i*_ = 0.39 ± 0.01 μM), (*E*)-2-(4-methylbenzylidene)hydrazine-1-carbothioamide **16** (*K*_*i*_ = 0.99 ± 0.04 μM), and *N*′-((1*E*,2*E*)-1,3-diphenylallylidene)hydrazinecarbothiohydrazide **29** (*K*_*i*_ = 2.23 ± 0.19 μM) were used in modeling studies that revealed sulfur ion coordination of the active site nickel ion and hydrogen bonds between the amide group and the side chain of Asp363 and Ala366 carbonyl moiety. Whole-cell studies proved the activity of compounds in Gram-positive and Gram-negative microorganisms. Ureolysis control observed in *P. mirabilis* PCM 543 (e.g., IC_50_ = 304 ± 14 μM for 1-benzyl-3-(4-(4-hydroxyphenyl)thiazol-2-yl)thiourea **52**) is a valuable achievement, as urease is recognized as a major virulence factor of this urinary tract pathogen.

## 1 Introduction

**Figure 1:**
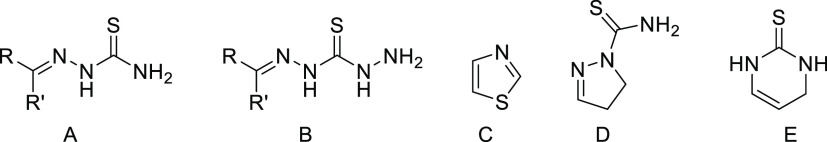
Structure of Monosubstituted Thiosemicarbazones (**A**), Monosubstituted Thiocarbohydrazones (**B**), Thiazole (**C**), 4,5-Dihydro-1*H*-pyrazole-1-carbothioamide (**D**), and 3,4-Dihydropyrimidine-2(1*H*)-thione (**E**).

Thiosemicarbazones are an important class of compounds endowed with biological properties, making them of interest to structural and medicinal chemists (the general structure of monosubstituted thiosemicarbazones is shown in Figure 1A). Many variations exist, including those where some or all of the nitrogen atoms are substituted by organic groups. Several thiosemicarbazones have been found to exhibit biological and medicinal properties, such as antimicrobial,^1,2^ antiviral,^3^ anticancer,^4^ and antitubercular qualities.^5^ In addition, their extraordinary complexing capacity for metal ions such as iron, copper, and zinc provides additional versatility as potential candidates for the preparation of coordination complexes.^6^

In addition to thiosemicarbazones, in recent years, there has been a special interest in the chemistry of thiocarbohydrazones, compounds that share the general formula depicted in Figure 1B and are considered the higher homologs of thiosemicarbazones.^7^

Thiazoles have emerged as important bioactive heterocycles and consist of a five-membered ring that contains nitrogen and sulfur (Figure 1C). Thiazole derivatives are studied extensively due to their important biological activities, such as anticancer,^8^ antimicrobial,^9^ anti-HIV,^10^ antimalarial,^11^ antihypertension,^12^ anti-inflammatory,^13^ and many others. The thiazole ring structure is present in commercial products, including sulfathiazole, ritonavir, penicillin, abafungin, and tiazofurin.^14^

4,5-Dihydro-1*H*-pyrazole-1-carbothioamide (Figure 1D) is a scaffold that draws attention as a potent therapeutic for several CNS diseases.^15^ It has also been proposed as a potential antidepressant^16^ and anticarcinogenic agent.^17^ Its other reported biological activities include antimicrobial,^18^ antiviral,^19^ anti-inflammatory,^20^ antitubercular,^21^ and antimalarial^22^ properties.

3,4-Dihydropyrimidine-2(1*H*)-thiones are heterocyclic compounds with a pyrimidine ring system (Figure 1E). Several biological activities have been reported for dihydropyrimidine-2-thiones, including anti-inflammatory^23^ and antioxidant activities.^24^ Pyrimidine is also a component of several biomolecules, such as nutrient molecules and nucleic acids, and plays an important role in different biological processes.^25^

Scaffolds **A**, **B**, **D**, and **E** in Figure 1 contain a structural fragment of thiourea, which can act as a substrate analogue for the reaction catalyzed by urea amidohydrolase (urease), an enzyme produced in several clinically relevant microorganisms but not in mammals. It catalyzes the hydrolysis of urea to ammonia and carbamate, which further decomposes spontaneously to another molecule of ammonia and carbon dioxide. Expression and activity of this enzyme are pivotal for the survival in the host organism and effective colonization in the case of several known species of bacterial and fungal pathogens. Nevertheless, it is not required for growth in optimal conditions and as such is considered one of the so-called non-essential targets for novel therapeutics. Urease-derived ammonia is toxic and causes tissue injury at the site of infection, and its protonation to ammonium ions disturbs physiological pH and sensitizes host systems to colonization.^26^ Urease enables *Helicobacter pylori* to spread within the gastric tract and *Cryptococcus neoformans* to cross the blood–brain barrier, whereas the enzyme activity in uropathogenic strains results in damage to the lower and upper urinary tract and recurring urinary tract infections (UTIs).^27^ Therefore, it is an extensively studied virulence factor of these pathogenic microorganisms.^28,29^ In light of the decades-long crisis in introducing new antibiotics, especially Gram-negative-active ones, antivirulence agents have become of great interest.^30^ This search has become more urgent as a consequence of SARS-CoV-2-related global antibiotic usage increases and thus predicted intensification of microbial drug-resistance spread.^31^

Thiourea is a fragment present in several urease inhibitors with kinetic properties reported mostly against urease of plant origin.^32^ In particular, thiosemicarbazone and thiazoline derivatives have been an object of considerable interest as potential urease inhibitors in recent years.^33,34^ Our research group has worked on the search for new urease inhibitors for several years.^35^ We have reported the relevance of various scaffolds, and this paper describes our first attempt to characterize thioureas.^36−40^ We exploited the aforementioned scaffolds that, with the exception of the thiazole ring (Figure 1C), contained thiourea fragments. Because thiazole had been reported for its antiureolytic activity,^41^ it was considered a promising scaffold as well and has been additionally modified to serve as a thiourea derivative. The inhibitory activity of the compounds was studied against purified bacterial enzyme and whole cells of ureolytic Gram-positive and Gram-negative microbial strains. In particular, monosubstituted thiosemicarbazones and thiocarbohydrazones, thiazolyl thioureas, 4,5-dihydro-1*H*-pyrazole-1-carbothioamides, and 3,4-dihydropyrimidine-2(1*H*)-thiones were synthesized and evaluated for their ureolytic activity.

The first two groups mentioned were particularly effective, achieving low micromolar values of *K*_*i*_ against *Sporosarcina pasteurii* urease. For monosubstituted thiocarbohydrazones, chalcone-derived 3,4-dihydropyrimidine-2(1*H*)-thiones, chalcone-derived 4,5-dihydro-1*H*-pyrazole-1-carbothioamides, and thiazolyl thioureas, to the best of our knowledge, this is the first report concerning their antiureolytic potency.

## 2 Results and Discussion

### 2.1 Chemistry

**Scheme 1:**
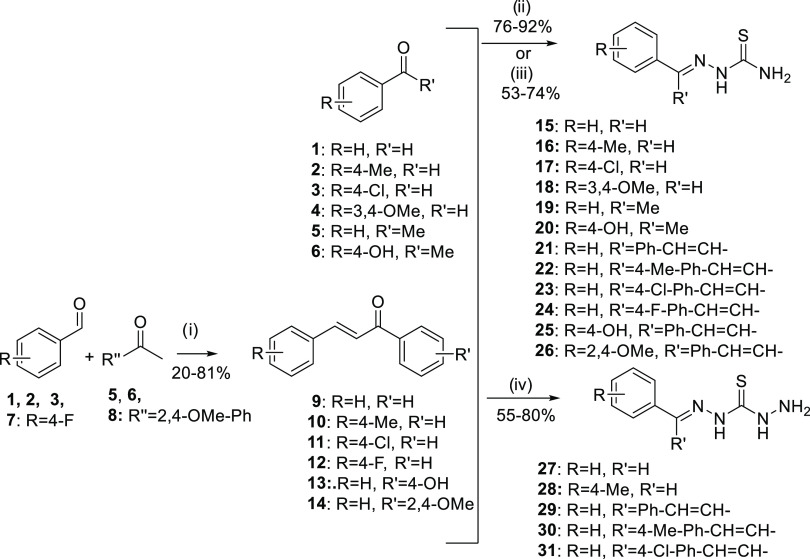
Syntheses of Thiosemicarbazones **15**–**26** and Thiocarbohydrazones **27**–**31** Reagents and conditions: (i) EtOH/H_2_O 1:1, NaOH, 0 °C, 1 h, rt, 24 h; (ii) **1**–**6**, thiosemicarbazide, EtOH 95%, cat. AcOH, 75 °C, 4 h; (iii) **9**–**14**, thiosemicarbazide, EtOH 95%, cat. HCl 6N, rt, 24 h; (iv) thiocarbohydrazide, **1**–**2** or **9**–**11**, EtOH 95%/H_2_O, cat. AcOH (for **1**–**2**) or cat. HCl 6N (for **9**–**11**), 80 °C, 3 h.

Thiosemicarbazones **15**–**20** were obtained in high yields in the reaction of the appropriate aromatic aldehyde **1**–**4** or acetophenone **5**–**6** with thiosemicarbazide in warm 95% ethanol using a catalytic amount of acetic acid. In each case, the stereochemistry of the double bond was *E*, as judged by the absence of a signal in the area of 14 ppm of ^1^H NMR, which is characteristic for *Z* isomers.^42^ Thiosemicarbazones **21**–**26** were obtained in medium yields by the reaction of the appropriate chalcone **9**–**14** (easily obtained from base-catalyzed Claisen-Schmidt condensation of aromatic aldehydes **1**–**3** and **7** with acetophenones **5**, **6**, and **8**) with thiosemicarbazide in 95% ethanol at room temperature (rt) using a catalytic amount of 6N HCl. Although a mixture of isomers was observed, stereoisomers with the double bonds in the *E* configuration were the most energy-favorable (Scheme 1).^43^

Thiocarbohydrazones **27**–**31** were obtained by reacting aldehydes **1** and **2** or chalcones **9**–**11** with thiocarbohydrazide in a mixture of warm ethanol/water using catalytic amounts of acetic acid or 6N HCl. The same trend concerning the double bonds was again observed: derivatives **27**–**28** were exclusively in the *E* configuration, while in derivatives **29**–**31**, a mixture of conformers was observed in ^1^H and ^13^C NMR, with *E*/*E* being predominant (Scheme 1).^43^

**Scheme 2:**
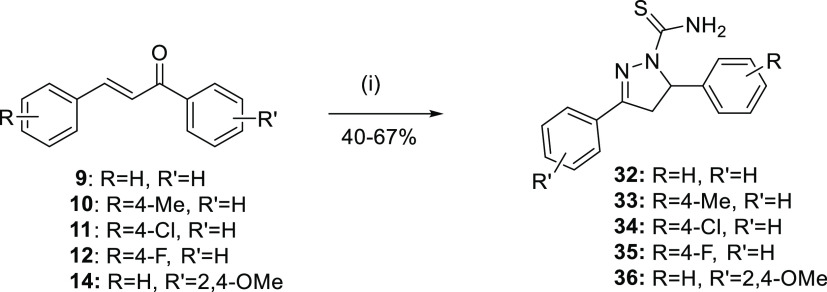
Synthesis of 1*H*-Pyrazole-1-carbothioamides **32**–**36** Reagents and conditions: (i) thiosemicarbazide, abs EtOH, NaOH, US, rt, 1 h.

**Scheme 3:**
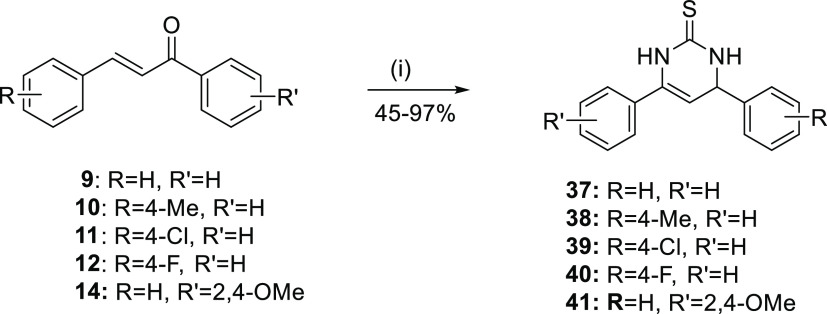
Synthesis of Dihydropyrimidine-2(1*H*)-thiones **37**–**41** Reagents and conditions: (i) thiourea, abs EtOH, NaOH, US, 50 °C, 1 h.

Chalcones’ constrained cyclized derivatives **32**–**41** were obtained by following the synthetic routes outlined in Schemes 2 and 3. Chalcones **9**–**14** reacted with thiosemicarbazide in basic conditions under ultrasonication to give 1*H*-pyrazole-1-carbothioamides **32**–**36** in medium yield. The ^1^H NMR spectra showed the characteristic ABX pattern of three protons of pyrazoline. Chalcones **9**–**14** reacted with thiourea under basic conditions and ultrasonication at 50 °C to give dihydropyrimidine-2(1*H*)-thiones **37**–**41** in medium to excellent yields.

**Scheme 4:**
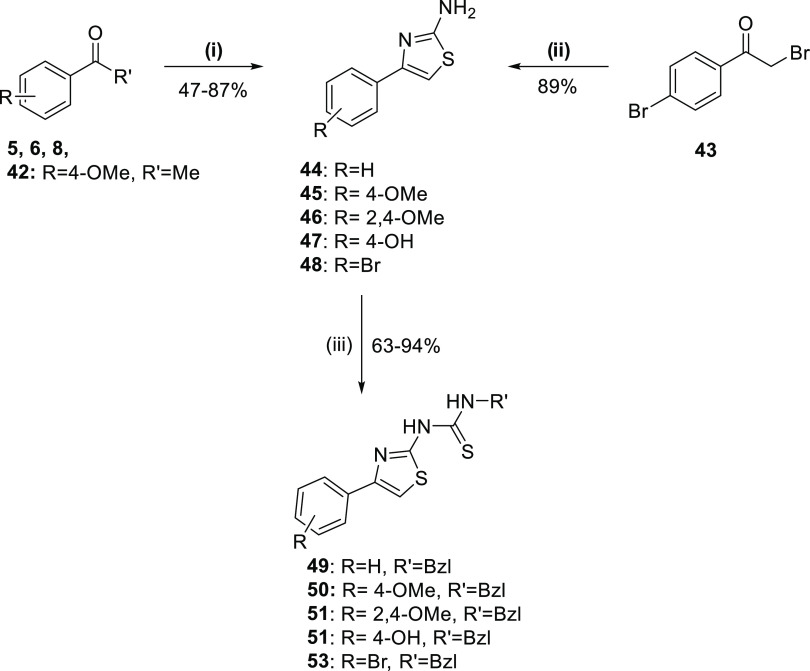
Synthesis of Thiazolyl Thioureas **49**–**53** Reagents and conditions: (i) thiourea, I_2_, 100 °C, 18 h, (ii) thiourea, abs EtOH, reflux, 80 °C, 18 h, (iii) thiourea, abs EtOH, reflux, 80 °C, 30 min, (iv) DMF, benzyl isothiocyanate, 70 °C, 2 days.

Aminothiazoles **44**–**48** were synthesized either by heating thiourea, the corresponding acetophenone **5**, **6**, **8**, **42**, and molecular iodine at 100 °C or by refluxing thiourea and the appropriate phenacyl bromide **43** in ethanol. Thioureas were then generated by condensation of aminothiazoles with benzyl isothiocyanate in dimethylformamide (DMF) at 70 °C for 2 days to give thiazolyl thiourea derivatives **49**–**53** in medium to good yields (Scheme 4).

### 2.2 Activity of Thiourea Derivatives against Bacterial Urease/SAR

The kinetic properties of the compounds as potential urease inhibitors were evaluated against native enzyme purified from *S. pasteurii* CCM 2056 culture. Residual enzyme activity was expressed as ammonia release from urea followed quantitively using the Berthelot reaction. All of the studied structures were reversibly inhibitory, with dissociation constants ranging from submicromolar to moderate micromolar concentrations (Table 1). The α quantifier between the enzyme-substrate-inhibitor complex (ESI) dissociation constant *K*_i_* and the EI dissociation constant *K*_*i*_ was calculated to determine the influence of substrate binding on the stability of the EI complex. Inhibitors revealed a mixed competitive type of inhibition except for compounds **20**, **21**, **25**, **26**, and **52**, which were noncompetitive activity reducers (see the Supporting Information for kinetic characteristics). For modeled compounds **19**, **16**, and **29**, the values of α were 152.01, 38.04, and 14.52, respectively. Values much higher than 1 indicated that the binding of urea significantly impaired the formation of the EI complex, which suggested that the mechanism of inhibition was closer to competitive for these structures. For comparison, the α value of compound **52** was 1.37. This result indicated that the binding of urea did not affect the inhibition in any way, neither positive nor negative, typical of a noncompetitive mode of inhibition. The available crystal structure of *S. pasteurii* urease was used to propose the mode of binding of the selected most active structures in the urease active site.^44^

**Table 1:**
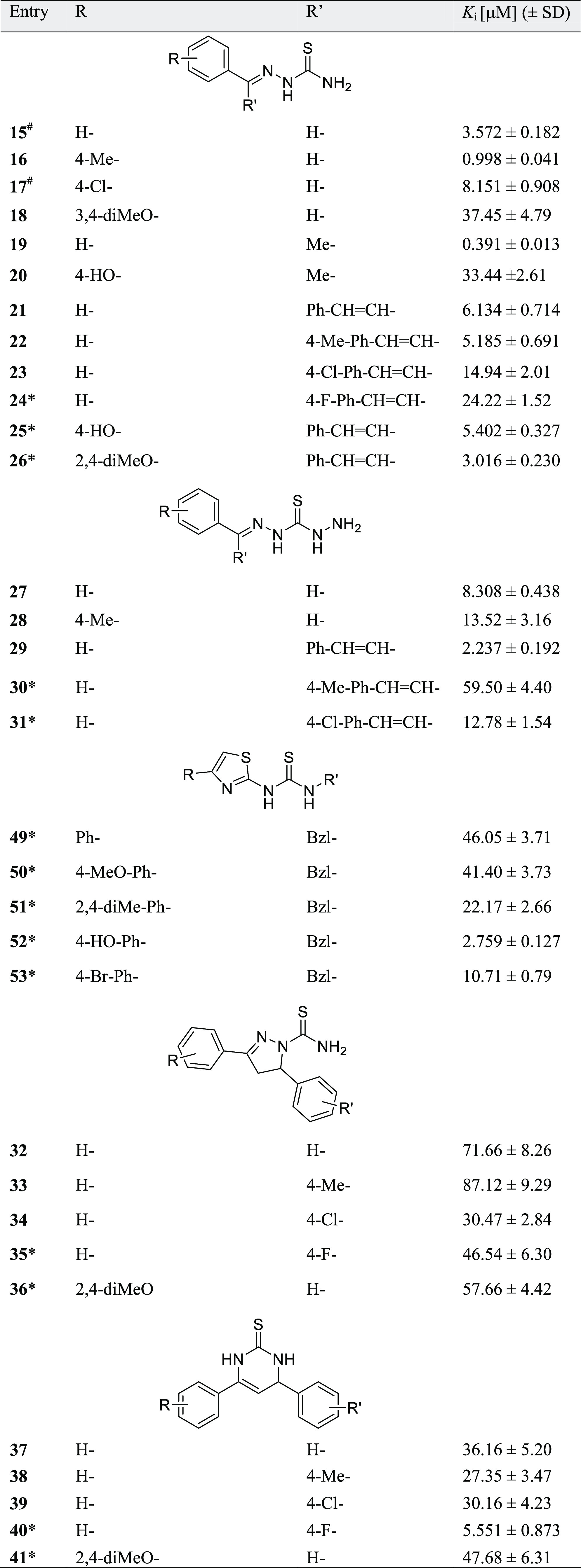
Inhibitory Activity of Thiourea Derivatives against Native *S. pasteurii* CCM 2056 Urease

a Inhibitory activity against jack bean urease reported in the literature: IC_50_ = 4.24 μM^45^ and *K*_*i*_ = 0.39 ± 0.04 μM^46^ for compounds **15** and **17**, respectively.

b Synthesis of the marked compounds is reported for the first time in this paper.

**Figure 2:**
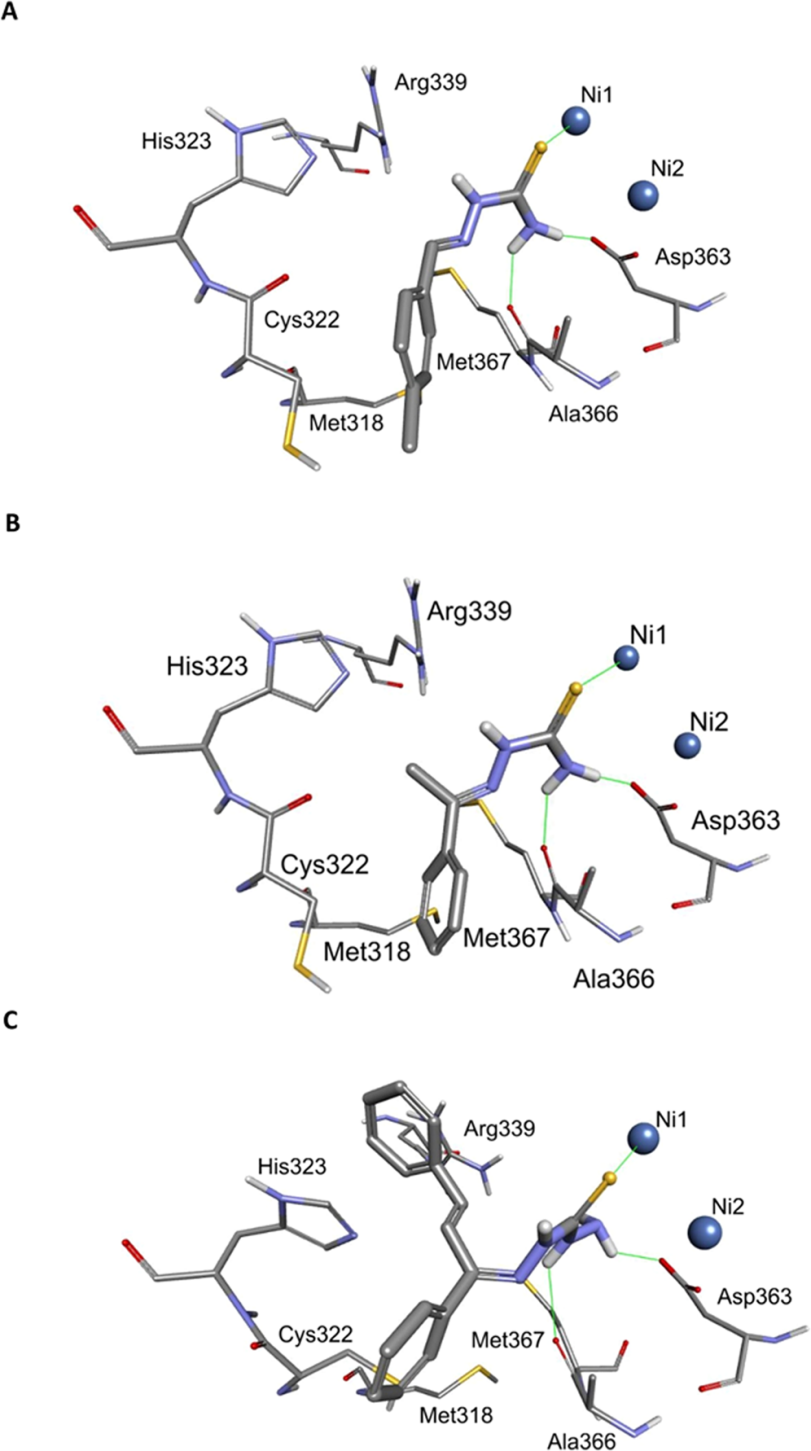
Modeled complexes of inhibitors **16** (**A**), **19** (**B**), and **29** (**C**) with *S. pasteurii* urease. Hydrogen bonds and interactions with nickel ions are shown as thin green lines.

The largest structural group of studied inhibitors, namely, thiosemicarbazones (**15**–**26**), can be divided into two subgroups—linear compounds **15**–**18** and branched compounds **19**–**26**. The entire group showed significant activity with *K*_*i*_ values in the low micromolar range (not exceeding 10 μM for 8 out of 12 compounds in this group), including the most potent structures in the study (inhibitors **16** and **19**, *K*_*i*_ = 0.998 and 0.391 μM, respectively). To our knowledge, these two compounds have not been studied as urease inhibitors before. Their mode of binding to *S. pasteurii* urease was modeled (Figure 2). As expected, the overall binding modes of **16** and **19** are very similar. The sulfur atom coordinates the nickel ion present in the active site, while the amide group forms hydrogen bonds with the Asp363 side chain and Ala366 carbonyl moiety. The aromatic fragment docks to the hydrophobic cavity formed by Met367, Ala366, Met318, and Cys322. The presence of an additional methyl group in compound **19** in comparison to inhibitor **16** does not significantly affect its binding mode.

Thiocarbohydrazones **27**–**31** also exhibited decent inhibitory activity with the most potent compound **29** with *K*_*i*_ = 2.24 μM, for which the model of binding to *S. pasteurii* urease was computed (Figure 2). The thiocarbohydrazone fragment interacts in a similar way to the above-described thiosemicarbazone moiety and forms interactions with nickel ions and hydrogen bonds with Asp363 and Ala366. Although the styryl group is relatively large, it fits between Arg339 and His323 and forms cation-π interactions with these amino acids’ side chains. The second phenyl group docks to the hydrophobic cavity formed by Met367, Ala366, Met318, and Cys322. In summary, all styryl-substituted compounds exhibit very similar potency.

Most benzyl-substituted thiazolyl thioureas **49**–**53** are less active than previously discussed thiosemicarbazones and thiocarbohydrazones. Most likely, the substitution of the thiourea fragment at both sides significantly impacts its possibility of binding to nickel ions in the active site. It is worth mentioning that the most active compound in this group, compound **52** (*K*_*i*_ = 2.76 μM), showed noncompetitive inhibition.

1*H*-Pyrazole-1-carbothioamides (**32**–**36**) and dihydropyrimidine-2(1*H*)-thiones (**37**–**41**) were generally less potent and showed moderate inhibitory activity. This could be attributed to the branched structure of these scaffolds, which does not allow fitting into the highly limited space of the active site of urease.

It is generally difficult to discuss the effectiveness of the studied thioureas in comparison with inhibitors reported by other groups: most data available in the literature were limited to IC_50_ values, which are highly dependent on the concentration of enzyme used in a study as opposed to the dissociation constant *K*_*i*_. Moreover, the inhibitory activity was determined against jack bean urease in most cases, which does not characterize their antivirulence relevance against pathogenic microorganisms.

### 2.3 Control of Whole-Cell Bacterial Ureolysis

The efficiency of the proposed inhibitors was verified by *in vitro* measurements of ammonia release from urea in urease-induced live cells of the Gram-positive *S. pasteurii* strain CCM 2056 and the Gram-negative representative of the uropathogenic species *P. mirabilis* PCM 543 (Table 2). The total progress curves of the urease reaction in the whole-cell system were inhibitor concentration-dependent and linear (*R*^2^ = 0.98), with no detectable initial delay. The number of cells introduced in the assays of the two strains was adjusted to ensure comparable levels of urease activity in untreated control reactions to provide IC_50_ quantification.

**Table 2:**
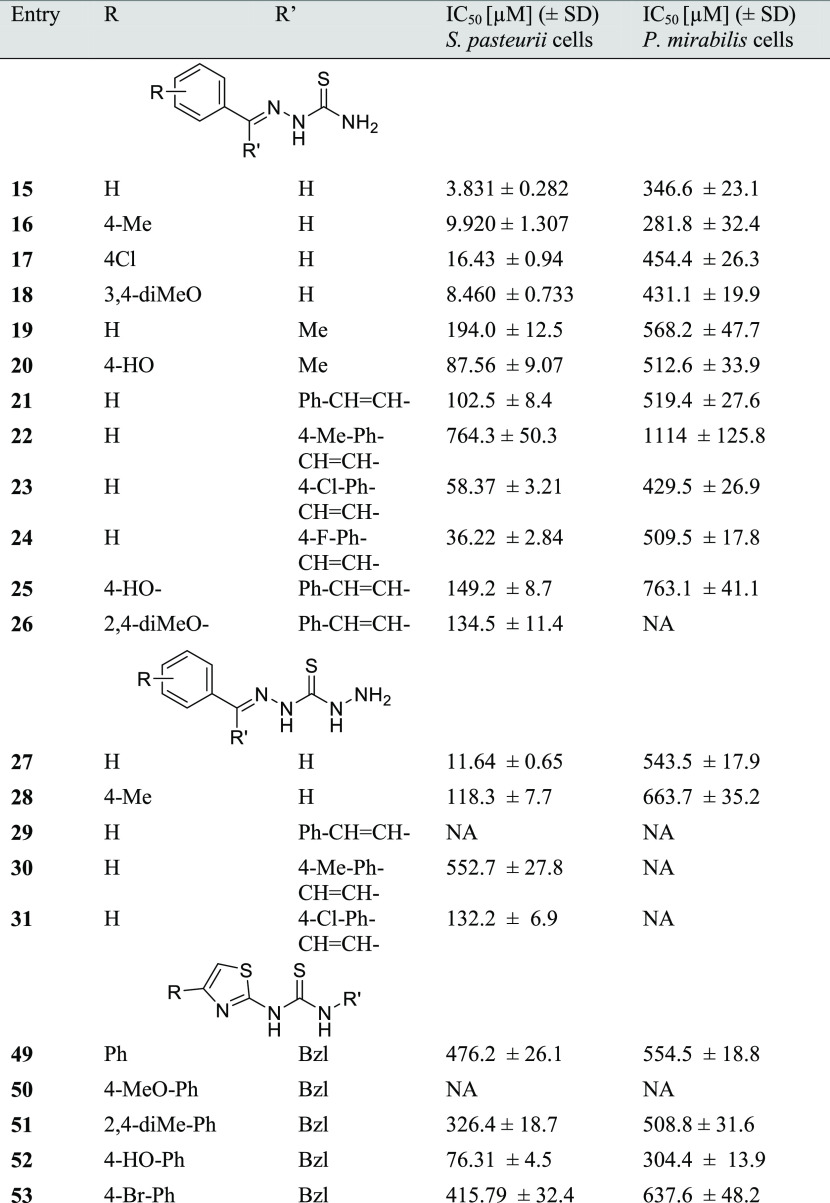
Urea Hydrolysis Inhibition in Live Cells of *S. pasteurii* CCM 2056 and *P. mirabilis* PCM 543

In the whole-cell assays, the influence of structure on the effectiveness of compounds (meaning diffusibility into the bacterial cell and interaction with target enzyme) was seen more clearly than during the assays using purified bacterial enzymes. This may indicate that the influence of implemented structural modifications is not limited to the sole process of EI complex formation. In most cases, methoxylation of the phenyl rings of the compound structure resulted in lower activity of the inhibitors compared to their nonmethoxylated counterparts. Such pairs of compounds were proposed among thiosemicarbazones (**15** and **18**, **21** and **26**) as well as thiazolyl thiourea derivatives (**49** and **50**). On the other hand, the presence of a hydroxyl substitution in phenyl rings tended to lower the IC_50_ values of the inhibitors in most cases. This favorable tendency was observed in both the Gram-positive and Gram-negative ureolytic bacterial strains, although the potency increase was much more significant for *S. pasteurii* cells. The most pronounced example was compound **52**, whose inhibitory activity against *S. pasteurii* cells decreased over 6-fold compared to its nonhydroxylated analogue **49**. Compound **52** was in fact the most active among the thiazolyl thiourea derivatives when applied to whole microbial cells. Compound **25**, being significantly less active against both strains than **21**, seemed to be the only exception to this rule. The relatively large structural characteristic of this pair of compounds was most likely an obstacle that could not be overcome by increasing the polarity by hydroxylation.

In Gram-positive *S. pasteurii* cells, the linear thiosemicarbazone derivatives (compounds **15**–**18**) were the most effective ureolysis inhibitors, with IC_50_ = 3.8 ± 0.2 for phenylthiosemicarbazone **15**. For *P. mirabilis*, it has been well established that designing small molecules to successfully affect Gram-negative species is a complex task. In these microorganisms, the asymmetrical outer membrane constitutes a highly efficient permeation barrier, and the efflux system further supports cell detoxification.^47^ Therefore, the observed ureolysis inhibitory effect of thiourea derivatives in *P. mirabilis* is noteworthy. The proposed compounds possess features that enable porin passage and Gram-negative cell accumulation, as they are moderately polar, rigid, and planar.^48^ It can be clearly seen, however, that the structure–activity pattern was flattened and most likely reflective of the uptake-efflux proportion in the studied strain. The inhibitor concentration needed to exert a half-reduction in *P. mirabilis* ureolysis was approximately 0.5 mM, with the exception of compound **22**, which was the least effective against both studied strains despite its favorable kinetic properties. The most promising effect was provided by thiosemicarbazone **16** (IC_50_ = 281.8 ± 32.4 μM) and thiazolyl thiourea derivative **52** (IC_50_ = 304.4 ± 13.9 μM). The overall potency of the proposed series of bacterial urease inhibitors exceeded the effectiveness of acetohydroxamate (AHA), approved for UTI treatment. In our earlier studies, we showed that 1 mM acetohydroxamate reduced whole-cell ureolysis in *P. mirabilis* PCM 543 to 42 ± 6%.^49^ Additionally, as we previously reported, the kinetic properties of AHA against purified enzymes from *S. pasteurii* and *P. mirabilis* showed *K*_*i*_ = 3.3 ± 0.4 and 5.7 ± 0.4 μM, respectively, an order of magnitude higher than we report now for the most efficient structures in the study.^50^

## 3 Conclusions

In the current paper, we verified a set of thiourea-based urease inhibitors as agents for the control of microbial ureolysis. All structures were inhibitory toward urease purified from *S. pasteurii*, with *K*_*i*_ values ranging from submicromolar to low-millimolar concentrations. Modeling studies performed for the most promising compounds confirmed appropriate structural features that allow fitting in the highly limited space of urease active site and provide coordination of nickel and hydrogen bond formation to exert the inhibitory effect. *In vitro* whole-cell studies in ureolytic microbial strains of *S. pasteurii* and *P. mirabilis* established our assumption on the utility of proposed scaffolds for the development of antivirulence compounds against urease-positive pathogens.

## 4 Experimental Section

### 4.1 Chemistry

Reagents were purchased from Aldrich and TCI Chemicals and were of analytical grade. Reactions were monitored by thin-layer chromatography (TLC) carried out on silica gel plates (Macherey Nagel SIL G UV254) and visualized in UV light. Purification of the compounds was performed by filtration or by column chromatography. Column chromatography was carried out on silica gel (Merck, 70–230 mesh) using the appropriate solvents. PE refers to petroleum ether 40–60 °C. ^1^H and ^13^C NMR spectra were recorded on a Bruker 400 MHz Avance spectrometer. 13C NMR spectra are fully proton-decoupled. Electrospray ionization (ESI) mass spectral analyses were performed on a mass spectrometer MSQ Surveyor, Finnigan, using direct sample injection in the positive or negative mode. High-resolution mass spectrometry (HRMS) spectra were registered using a 4800 MALDI-TOF mass spectrometer (Applied Biosystems, Foster City) in the positive reflection mode in the *m*/*z* range of 100–700. All synthesized compounds gave satisfactory NMR spectra. All tested compounds possessed purity > 95% based on HRMS. Data were processed using Compass Data Analysis software and MestReNova v.14.1.1.

#### 4.1.1 General Method for the Synthesis of Chalcones **9**–**14**, A

To a stirred solution of NaOH (2.20 g, 55 mmol) in EtOH/H_2_O 1:1 (250 mL), the corresponding acetophenone **5**, **6**, **8** (50 mmol) was added. The mixture was cooled to 0 °C followed by the slow addition of the aromatic aldehyde **1**–**3**, **7** (50 mmol) dissolved in EtOH (50 mL). The reaction mixture slowly reached room temperature and was stirred for 24 h. The solid formed was filtered, washed with EtOH/H_2_O 1:1, and dried over P_2_O_5_.

##### 4.1.1.1 (*E*)-Chalcone (**9**)^51^

Following the general method A, using benzaldehyde **1** and acetophenone **5**, compound **9** was obtained as a white solid in 81% yield. *R*_f_ = 0.63 (PE/AcOEt 9.5:0.5). ^1^H NMR (400 MHz, DMSO): δ 8.15–8.14 (m, 2H), 7.95–7.88 (m, 3H), 7.77–7.74 (m, 1H), 7.67–7.65 (m, 1H), 7.58 (m, 2H), 7.47–7.45 (m, 3H).^13^C NMR (101 MHz, DMSO): δ 189.7, 144.5, 138.1, 135.2, 133.6, 131.0, 129.4, 129.4, 129.3, 129.0, 122.6. NMR spectrum is in agreement with the data reported in the literature.

##### 4.1.1.2 (*E*)-1-Phenyl-3-(*p*-tolyl)prop-2-en-1-one (**10**)^52^

Following the general method A, using *p*-tolualdehyde **2** and acetophenone **5**, compound **10** was obtained as a white solid in 67% yield. *R*_f_ = 0.70 (PE/AcOEt 9:1). ^1^H NMR (400 MHz, DMSO): 8.16 (d, 2H, *J* = 7.6 Hz), 7.88 (d, 1H, *J* = 15.6 Hz), 7.78 (d, 2H, *J* = 8.0 Hz), 7.74–7.65 (m, 2H), 7.59–7.55 (m, 2H), 7.28 (d, 2H, *J* = 7.9 Hz), 2.35 (s, 3H). ^13^C NMR (101 MHz, DMSO): δ 190.0, 144.1, 140.3, 137.6, 133.0, 131.9, 129.5, 128.9, 128.7, 128.4, 121.0, 21.0. NMR spectrum is in agreement with the data reported in the literature.

##### 4.1.1.3 (*E*)-3-(4-Chlorophenyl)-1-phenylprop-2-en-1-one (**11**)^53^

Following the general method A, using 4-chlorobenzaldehyde **3** and acetophenone **5**, compound **11** was obtained as a white solid in 82% yield. *R*_f_ = 0.76 (PE/AcOEt 9:1) ^1^H NMR (400 MHz, DMSO): δ 8.16 (d, 2H, *J* = 6.9 Hz), 7.99–7.93 (m, 3H), 7.75–7.65 (m, 2H), 7.60–7.52 (m, 4H). ^13^C NMR (101 MHz, DMSO): δ = 189.0, 142.5, 137.4, 135.1, 133.6, 133.2, 130.5, 128.9, 128.7, 128.5, 122.7. NMR spectrum is in agreement with the data reported in the literature.

##### 4.1.1.4 (*E*)-3-(4-Fluorophenyl)-1-phenylprop-2-en-1-one (**12**)^53^

Following the general method A, using 4-fluorobenzaldehyde **7** and acetophenone **5**, compound **12** was obtained as a white solid in 67% (1.94 g). *R*_f_ = 0.72 (PE/AcOEt 9:1). ^1^H NMR (400 MHz, DMSO): δ 8.15 (d, 2H, *J* = 7.3 Hz), 7.98–7.88 (m, 3H), 7.76–7.66 (m, 2H), 7.60–7.56 (m, 2H, *J* = 6.9 Hz), 7.33–7.29 (m, 2H, *J* = 8.6 Hz). ^13^C NMR (101 MHz, DMSO): δ = 189.0, 164.4 and 162.4 (^1^*J*_C-F_ = 249.5 Hz), 142.7, 137.5, 133.1, 131.3 and 131.3 (^4^*J*_C-F_ = 3.4 Hz), 131.2, 131.2 and 131.2 (^3^*J*_C-F_ = 8.3 Hz), 128.7 (2c), 128.5 (2c), 121.9 and 121.9 (^5^*J*_C-F_ = 2.4 Hz), 116.0 and 115.8 (^2^*J*_C-F_ = 21.9 Hz). NMR spectrum is in agreement with the data reported in the literature.

##### 4.1.1.5 (*E*)-1-(4-Hydroxyphenyl)-3-phenylprop-2-en-1-one (**13**)^53^

Following the general method A, using benzaldehyde **1** and 4-hydroxyacetophenone **6**, compound **13** was obtained as a white solid in 20% yield. *R*_f_ = 0.25 (PE/AcOEt 9:1). ^1^H NMR (400 MHz, DMSO): δ 10.38 (s, 1H), 8.07 (d, 2H, *J* = 8.6 Hz), 7.94–7.82 (m, 3H), 7.72–7.64 (m, 1H), 7.46–7.44 (m, 3H), 6.91 (d, 2H, *J* = 8.6 Hz). ^13^C NMR (101 MHz, DMSO): δ 187.1, 162.3, 142.7, 134.8, 131.1, 130.3, 129.0, 128.8, 128.7, 122.1, 115.4. NMR spectrum is in agreement with the data reported in the literature.

##### 4.1.1.6 (*E*)-1-(2,4-Dimethoxyphenyl)-3-phenylprop-2-en-1-one (**14**)

Following the general method A, using benzaldehyde **1** and 2,4-dimethoxyacetophenone **8**, compound **13** was obtained as a white solid in 76% yield. *R*_f_ = 0.75 (PE/AcOEt 9:1). ^1^H NMR (400 MHz, DMSO): δ 7.74–7.70 (m, 2H), 7.62 (d, 1H, *J* = 7.5 Hz), 7.55–7.54 (m, 2H), 7.45–7.43 (m, 3H), 6.74–6.62 (m, 2H), 3.91 (s, 3H), 3.86 (s, 3H). ^13^C NMR (101 MHz, DMSO): δ 190.1, 164.5, 160.7, 141.9, 135.1, 132.6, 130.8, 129.5, 128.8, 128.5, 127.4, 121.6, 106.6, 98.9, 56.4, 56.0.

#### 4.1.2 General Method for the Synthesis of Thiosemicarbazone **15**–**20**, B

To a stirred solution of the aromatic aldehyde **1**–**4** or ketone **5**–**6** (1 mmol) in 95% ethanol (7 mL), thiosemicarbazide (91 mg, 1 mmol) was added followed by the addition of a few drops of acetic acid. The reaction mixture was warmed at 75 °C for 4 h and then water (7 mL) was added. The precipitate was filtered, washed with ethanol/water 1:1, and dried over P_2_O_5_.

#### 4.1.3 General Method for the Synthesis of Thiosemicarbazones **21**–**26**, C

To a stirred solution of the chalcone **9**–**14** (1 mmol) in 95% ethanol (25 mL), thiosemicarbazide (91 mg, 1 mmol) was added followed by the addition of a few drops of 6N HCl. The reaction mixture was stirred at room temperature for 24 h after which it was purified by column chromatography using a mixture of PE (40–60°C)/AcOEt as eluent.

##### 4.1.3.1 (*E*)-2-Benzylidenehydrazine-1-carbothioamide (**15**)^54^

Following the general method B, using benzaldehyde **1**, compound **15** was obtained as a white solid in 77% yield. *R*_f_ = 0.42 (PE/AcOEt 2:1). ^1^H NMR (400 MHz, DMSO): δ 11.41 (s, 1H), 8.18 (s, 1H), 8.05 (s, 1H), 7.98 (s, 1H), 7.81–7.77 (m, 2H), 7.39 (s, 3H). ^13^C NMR (101 MHz, DMSO): δ 127.8, 129.1, 134.7, 134.7, 142.7, 178.5. NMR spectrum is in agreement with the data reported in the literature. MS (ESI) calculated for C_8_H_10_N_3_S^+^ (M + H) 180.05, found 180.05. HRMS *m*/*z* calculated for C_8_H_10_N_3_S^+^ [M + H] 180.0590, found 180.0590.

##### 4.1.3.2 (*E*)-2-(4-Methylbenzylidene)hydrazine-1-carbothioamide (**16**)^54^

Following the general method B, using *p*-toluolaldehyde **2**, compound **16** was obtained as a white solid in 80% yield. *R*_f_ = 0.49 (PE/AcOEt 2:1). ^1^H NMR (400 MHz, DMSO): δ 11.35 (s, 1H), 8.13 (s, 1H) 8.01 (s, 1H), 7.92 (s, 1H), 7.68 (d, 2H, *J* = 6.6 Hz), 7.21 (d, 2H, *J* = 6.7 Hz), 2.32 (s, 3H). ^13^C NMR (101 MHz, DMSO): δ 175.3, 139.9, 137.1, 124.7, 18.8. NMR spectrum is in agreement with the data reported in the literature. MS (ESI) calculated for C_9_H_12_N_3_S^+^ (M + H) 194.07, found 194.00. HRMS *m*/*z* calculated for C_9_H_12_N_3_S^+^ [M + H] 194.0746, found 194.0745.

##### 4.1.3.3 (*E*)-2-(4-Chlorobenzylidene)hydrazine-1-carbothioamide (**17**)^55^

Following the general method B, using 4-chlorobenzaldehyde **3**, compound **17** was obtained as a white solid in 82% yield. *R*_f_ = 0,67 (PE/AcOEt 2:1). ^1^H NMR (400 MHz, DMSO): 11.46 (s, 1H), 8.06 (s, 1H), 8.03 (s, 1H), 7.84 (d, *J* = 7.9 Hz, 2H), 7.46 (d, 2H, *J* = 8.0 Hz). NMR (101 MHz, DMSO): δ 178.0, 140.8, 134.2, 133.1, 128.9, 128.6. NMR spectrum is in agreement with the data reported in the literature. MS (ESI) calculated for C_8_H_9_ClN_3_S^+^ (M + H) 214.02, found 214.02. HRMS *m*/*z* calculated for C_8_H_9_ClN_3_S^+^ [M + H] 214.0200, found 214.0202.

##### 4.1.3.4 (*E*)-2-(3,4-Dimethoxybenzylidene)hydrazine-1-carbothioamide (**18**)^55^

Following the general method B, using 3,4-dimethoxybenzaldehyde **4**, compound **18** was obtained as an off-white solid in 92% yield. *R*_f_ = 0.45 (PE/AcOEt 1:4). ^1^H NMR (400 MHz, DMSO): δ 11.30 (s, 1H), 8.14 (s, 1H), 8.00 (s, 1H), 7.97 (s, 1H), 7.51 (s, 1H), 7.13 (d, 1H, *J* = 8.0 Hz), 6.95 (d, 1H, *J* = 7.7 Hz). ^13^C NMR (101 MHz, DMSO): δ 178.0, 151.0, 149.5, 143.0, 127.3, 122.6, 111.6, 108.9, 56.1, 55.9. NMR spectrum is in agreement with the data reported in the literature. MS (ESI) calculated for C_10_H_14_N_3_O_2_S^+^ (M + H) 240.08 found 240.32. HRMS *m*/*z* calculated for C_10_H_14_N_3_O_2_S^+^ [M + H] 240.0801, found 240.0800.

##### 4.1.3.5 (*E*)-2-(1-Phenylethylidene)hydrazine-1-carbothioamide (**19**)^55^

Following the general method B, using acetophenone **5**, compound **19** was obtained as an off-white solid in 68% yield. *R*_f_ = 0.45 (PE/EA 2:1) ^1^H NMR (400 MHz, DMSO): δ 10.20 (s, 1H), 8.26 (s, 1H), 7.91 (s, 3H), 7.38 (s, 3H), 2.30 (s, 3H). ^13^C NMR (101 MHz, DMSO): δ 179.0, 148.4, 137.7, 129.5, 128.5, 126.7, 14.2. NMR spectrum is in agreement with the data reported in the literature. MS (ESI) calculated for C_9_H_12_N_3_S^+^ (M + H) 194.08 found 194.09. HRMS *m*/*z* calculated for C_9_H_12_N_3_S^+^ [M + H] 194.0746, found 194.0746.

##### 4.1.3.6 (*E*)-2-(1-(4-Hydroxyphenyl)ethylidene)hydrazine-1-carbothioamide (**20**)^56^

Following the general method B, using 4-hydroxyacetophenone **6**, compound **20** was obtained as a beige solid in 76% yield. *R*_f_ = 0.54 (PE/AcOEt 2:1). ^1^H NMR (400 MHz, DMSO): δ 10.05 (s, 1H), 9.71 (s, 1H), 8.15 (s, 1H), 7.79–7.75 (m, 3H), 6.75 (d, 2H, *J* = 7.6 Hz), 2.23 (s, 3H). ^13^C NMR (101 MHz, DMSO): δ 178.6, 158.9, 148.8, 128.7, 128.5, 115.3, 14.4. NMR spectrum is in agreement with the data reported in the literature. MS (ESI) calculated for C_9_H_12_N_3_OS^+^ (M + H) 210.08 found 210.14. HRMS *m*/*z* calculated for C_9_H_12_N_3_OS^+^ [M + H] 210.0696, found 210.0697.

##### 4.1.3.7 (*E*)-2-((*E*)-1,3-Diphenylallylidene)hydrazine-1-carbothioamide (**21**)^57^

Following the general method C using (*E*)-chalcone (**9**), compound **21** was obtained as an off-yellow solid in 66% yield. *R*_f_ = 0.73 (PE/AcOEt 8:2). ^1^H NMR (400 MHz, DMSO): δ 11.09 (s, 1H), 8.49 (s, 1H), 8.30 (s, 1H), 7.83–7.63 (m, 5H), 7.44–7.33 (m, 6H), 6.76 (d, 1H, *J* = 16.0 Hz). ^13^C NMR (101 MHz, DMSO): δ 178.9, 148.2, 139.6, 136.9, 135.8, 129.8, 129.2, 128.6, 128.3, 127.9, 126.9, 118.7. NMR spectrum is in agreement with the data reported in the literature. MS (ESI) calculated for C_16_H_15_N_3_S^+^ (M + H) 282.11 found 282.27. HRMS *m*/*z* calculated for C_16_H_16_N_3_S^+^ [M + H] 282.1059, found 282.1060.

##### 4.1.3.8 (*E*)-2-((*E*)-1-Phenyl-3-(*p*-tolyl)allylidene)hydrazine-1-carbothioamide (**22**)^58^

Following the general method C using chalcone (**10**), compound **22** was obtained as an off-yellow solid in 71%. *R*_f_ = 0.66 (PE/AcOEt 8:2). ^1^H NMR (400 MHz, DMSO): δ 11.08 (s, 1H), 8.60 (s, 1H), 8.29 (s, 1H), 7.79–7.75 (m, 1H), 7.64–7.59 (m, 4H), 7.44 (m, 2H), 7.33–7.31 (m, 1H), 7.21–7.14 (m, 2H), 6.72 (d, 1H, *J* = 16.0 Hz), 2.31 (s, 3H). ^13^C NMR (101 MHz, DMSO): δ 178.9, 150.7, 148.4, 139.7, 139.1, 136.9, 133.1, 129.7, 129.2, 128.2, 126.8, 117.7, 101.5, 21.0. NMR spectrum is in agreement with the data reported in the literature. MS (ESI) calculated for C_17_H_18_N_3_S^+^ (M + H) 296.12 found 296.10. HRMS *m*/*z* calculated for C_17_H_18_N_3_S^+^ [M + H] 296.1216, found 296.1215.

##### 4.1.3.9 (*E*)-2-((*E*)-3-(4-Chlorophenyl)-1-phenylallylidene)hydrazine-1-carbothioamide (**23**)^57^

Following the general method C using chalcone (**11**), compound **23** was obtained as an off-yellow solid in 74% yield. *R*_f_ = 0.54 (PE/AcOEt 9:1). ^1^H NMR (400 MHz, DMSO): δ 11.14 (s, 1H), 8.33 (s, 1H), 7.83–7.74 (m, 3H), 7.62–7.61 (m, 2H), 7.48–7.34 (m, 5H), 6.76 (d, 1H, *J* = 16.0 Hz). ^13^C NMR (101 MHz, DMSO): δ 178.9, 147.8, 138.1, 136.7, 134.8, 133.7, 129.6, 129.2, 128.9, 128.6, 128.3, 119.5. NMR spectrum is in agreement with the data reported in the literature. MS (ESI) calculated for C_16_H_14_ClN_3_S^+^ (M + H) 316.07 found 316.10. HRMS *m*/*z* calculated for C_16_H_15_ClN_3_S^+^ [M + H] 316.0670, found 316.0671.

##### 4.1.3.10 (*E*)-2-((*E*)-3-(4-Fluorophenyl)-1-phenylallylidene)hydrazine-1-carbothioamide (**24**)

Following the general method C using chalcone (**12**), compound **24** was obtained as an off-yellow solid in 58% yield. *R*_f_ = 0.86 (PE/AcOEt 8:2). ^1^H NMR (400 MHz, DMSO): δ 11.07 (s, 1H), 8.60 (s, 1H), 8.30 (s, 1H), 7.79–7.76 (m, 3H), 7.63–7.62 (m, 2H), 7.53–7.35 (m, 3H), 7.26–7.13 (m, 2H), 6.77 (d, 1H, *J* = 16.1 Hz). ^13^C NMR (101 MHz, DMSO): δ 178.9, 162.7 (d, *J* = 247.3 Hz), 148.0, 138.3, 136.8, 132.5, 130.1 (d, *J* = 8.3 Hz), 129.2, 129.0, 128.3, 118.6, 115.6 (d, *J* = 21.6 Hz). MS (ESI) calculated for C_16_H_15_FN_3_S^+^ (M + H) 300.10 found 300.05. HRMS *m*/*z* calculated for C_16_H_15_FN_3_S^+^ [M + H] 300.0965, found 300.0964.

##### 4.1.3.11 (*E*)-2-((*E*)-1-(4-Hydroxyphenyl)-3-phenylallylidene)hydrazine-1-carbothioamide (**25**)

Following the general method C using chalcone (**13**), compound **25** was obtained as an off-yellow solid in 53% yield. *R*_f_ = 0.70 (PE/AcOEt 8:2). ^1^H NMR (400 MHz, DMSO): δ 10.86 (s, 1H), 9.74 (s, 1H), 8.52 (s, 1H), 8.21 (s, 1H), 7.72 (d, 2H, *J* = 7.7 Hz), 7.49–7.46 (m, 2H), 7.40–7.30 (m, 3H), 7.16–7.10 (m, 1H), 7.02–7.00 (m, 1H), 6.83–6.78 (m, 2H). ^13^C NMR (101 MHz, DMSO): δ 178.6, 158.5, 148.5, 139.5, 135.9, 130.6, 128.9, 128.7, 127.9, 126.9, 119.1, 115.1. MS (ESI) calculated for C_16_H_15_N_3_OS^+^ (M + H) 298.10 found 298.11. HRMS *m*/*z* calculated for C_16_H_16_N_3_OS^+^ [M + H] 298.1009, found 298.1009.

##### 4.1.3.12 (*E*)-2-((*E*)-1-(2,4-Dimethoxyphenyl)-3-phenylallylidene)hydrazine-1-carbothioamide (**26**)

Following the general method C chalcone (**14**), compound **26** was obtained as an off-yellow solid in 70% yield. *R*_f_ = 0.57 (PE/AcOEt 8:2). ^1^H NMR (400 MHz, DMSO): δ 11.20 (s, 1H), 8.54 (s, 1H), 8.03 (s, 1H), 7.46–7.44 (m, 2H), 7.35–7.29 (m, 3H), 7.13–7.01 (m, 2H), 6.81 (s, 1H), 6.77–6.75 (m, 1H), 6.41 (d, 1H, *J* = 16.2 Hz), 3.87 (s, 3H), 3.78 (s, 3H). ^13^C NMR (101 MHz, DMSO): δ 177.6, 162.1, 157.5, 148.3, 135.8, 130.5, 128.8, 128.6, 126.8, 110.5, 106.4, 99.4, 55.9, 55.4. MS (ESI) calculated for C_18_H_19_N_3_O_2_S^+^ (M + H) 342.13 found 342.16. HRMS *m*/*z* calculated for C_18_H_20_N_3_O_2_S^+^ [M + H] 342.1271, found 342.1272.

#### 4.1.4 General Method for the Synthesis of Thiocarbohydrazones **27**–**31**, D

To a stirred solution of the aromatic aldehyde **1**–**2** or chalcone **9**–**11** (1 mmol) in a mixture of 95% ethanol (30 mL) and water (6 mL), thiocarbohydrazide (133 mg, 1 mmol) was added followed by the addition of a few drops of acetic acid (in the case of aromatic aldehydes **1**–**2**) or concentrated HCl (in the case of chalcones **9**–**11**), and the reaction mixture was heated to 75 °C for 4 h. Then, it was left to stand at room temperature for 24 h. The precipitate was filtered, washed with ethanol/water 1:1, and dried over P_2_O_5_.

##### 4.1.4.1 (*E*)-*N*′-Benzylidenehydrazinecarbothiohydrazide (**27**)^59^

Following the general method D for aromatic aldehydes, using benzaldehyde **1**, compound **27** was obtained as a white solid in 75% yield. *R*_f_ = 0.73 (CH_2_Cl_2_/MeOH 9.5:0.5). ^1^H NMR (400 MHz, DMSO): δ 11.41 (s, 1H), 9.80 (s, 1H), 8.01 (s, 1H), 7.85–7.80 (m, 2H), 7.42–7.35 (m, 3H), 4.87 (s, 2H). ^13^C NMR (101 MHz, DMSO): δ 176.0, 141.9, 134.1, 129.8, 128.8, 127.3. NMR spectrum is in agreement with the data reported in the literature. MS (ESI) calculated for C_8_H_11_N_4_S^+^ (M + H) 195.07 found 195.08. HRMS *m*/*z* calculated for C_8_H_11_N_4_S^+^ [M + H] 195.0699, found 195.0699.

##### 4.1.4.2 (*E*)-*N*′-(4-Methylbenzylidene)hydrazinecarbothiohydrazide (**28**)^60^

Following the general method D for aromatic aldehydes, using *p*-tolualdehyde **2**, compound **28** was obtained as an off-white solid in 80%. *R*_f_ = 0.48 (PE/AcOEt). ^1^H NMR (400 MHz, DMSO): δ 11.35 (s, 1H), 9.73 (s, 1H), 7.97 (s, 1H), 7.71 (d, 2H, J = 9.8 Hz), 7.20 (d, 2H, J = 8.7 Hz), 4.85 (s, 2H), 2.32 (s, 3H). ^13^C NMR (101 MHz, DMSO): δ 175.8, 142.1, 139.4, 131.5, 129.2, 127.3, 21.0. NMR spectrum is in agreement with the data reported in the literature. MS (ESI) calculated for C_9_H_13_N_4_S^+^ (M + H) 209.10 found 209.22. HRMS *m*/*z* calculated for C_9_H_13_N_4_S^+^ [M + H] 209.0855, found 209.0857.

##### 4.1.4.3 *N*′-((1*E*,2*E*)-1,3-Diphenylallylidene)hydrazinecarbothiohydrazide (**29**)^43^

Following the general method D for chalcones, using chalcone (**9**), compound **29** was obtained as a beige solid in 55% yield. *R*_f_ = 0.87 (1-BuOH/AcOH/H_2_O 4:1:1). ^1^H NMR (400 MHz, DMSO): δ 11.05 (s, 1H), 9.58 (s, 1H), 7.79–7.65 (m, 4H), 7.45–7.32 (m, 7H), 6.77 (d, 1H, *J* = 16.0 Hz), 4.98–4.96 (m, 2H). ^13^C NMR (101 MHz, DMSO): δ 176.0, 148.1, 139.3, 136.8, 135.8, 129.7, 129.2, 128.9, 128.6, 128.2, 127.9, 118.6. MS (ESI) calculated for C_16_H_16_N_4_S^+^ (M + H) 297.11 found 297.10. HRMS *m*/*z* calculated for C_16_H_17_N_4_S^+^ [M + H] 297.1168, found 297.1167.

##### 4.1.4.4 *N*′-((1*E*,2*E*)-1-Phenyl-3-(*p*-tolyl)allylidene)hydrazinecarbothiohydrazide (**30**)

Following the general method D for chalcones, using chalcone (**10**), compound **30** was obtained as a beige solid in 59%. *R*_f_ = 0.86 (1-BuOH/AcOH/H_2_O 4:1:1). ^1^H NMR (400 MHz, DMSO): δ 11.03 (s, 1H), 9.54 (s, 1H), 7.73 (d, 1H, *J* = 16.0 Hz), 7.63–7.60 (m, 4H), 7.44–7.43 (m, 2H), 7.35–7.31 (m, 1H), 7.22–7.16 (m, 2H), 6.73 (d, 1H, *J* = 16.0 Hz), 4.93–4.91 (m, 2H), 2.32 (s, 3H). ^13^C NMR (101 MHz, DMSO): δ 176.0, 148.3, 139.4, 139.0, 136.9, 133.1, 129.2, 128.9, 128.2, 127.9, 126.7, 117.6, 21.0. MS (ESI) calculated for C_17_H_19_N_4_S^+^ (M + H) 311.13 found 311.05. HRMS *m*/*z* calculated for C_17_H_19_N_4_S^+^ [M + H] 311.1325, found 311.1323.

##### 4.1.4.5 *N*′-((1*E*,2*E*)-3-(4-Chlorophenyl)-1-phenylallylidene)hydrazinecarbothiohydrazide (**31**)

Following the general method D for chalcones, using chalcone (**11**), compound **31** was obtained as an off-orange solid in 80% yield. *R*_f_ = 0.91 (1-BuOH/AcOH/H_2_O 4:1:1). ^1^H NMR (400 MHz, DMSO): δ 11.07 (s, 1H), 9.60 (s, 1H), 7.77–7.75 (m, 2H), 7.65–7.63 (m, 3H), 7.47–7.40 (m, 4H), 7.33–7.32 (m, 1H), 6.78–6.76 (m, 1H), 4.94–4.92 (m, 2H). ^13^C NMR (101 MHz, DMSO): δ 176.4, 148.1, 138.2, 137.2, 135.8, 134.1, 130.1, 129.8, 129.5, 129.1, 128.7, 119.9. MS (ESI) calculated for C_16_H_16_ClN_4_S^+^ (M + H) 331.08 found 331.11. HRMS *m*/*z* calculated for C_16_H_16_ClN_4_S^+^ [M + H] 331.0779, found 331.0780.

#### 4.1.5 General Method for the Synthesis of H-Pyrazole-carbothioamides **32**–**36**, E

In a round-bottom flask, NaOH (100 mg, 2.50 mmol) is dissolved in absolute EtOH (2.5 mL) followed by the addition of the appropriate chalcone **9**–**12**, **14**, and thiosemicarbazide (91 mg, 1.00 mmol). The mixture was sonicated at room temperature for 1 h. The content was poured into ice water, left to stand for 24 h, and the solid was filtered, washed with a mixture of EtOH/H_2_O 1:1, and left to stand over P_2_O_5_.

##### 4.1.5.1 3,5-Diphenyl-4,5-dihydro-1*H*-pyrazole-1-carbothioamide (**32**)^61^

Following the general method E using chalcone (**9**), compound **32** was obtained as a white solid in 67% yield. *R*_f_ = 0.38 (PE/AcOEt 9:1). ^1^H NMR (400 MHz, DMSO): δ 8.02 (s, 1H), 7.89–7.87 (m, 3H), 7.46–7.44 (m, 3H), 7.33–7.29 (m, 2H), 7.24–7.20 (m, 1H), 7.13 (d, 2H, *J* = 7.7 Hz), 5.93 (dd, 1H, *J* = 11.4, 3.2 Hz), 3.91 (dd, 1H, *J* = 18.0, 11.5 Hz), 3.14 (dd, 1H, *J* = 18.0, 3.3 Hz). ^13^C NMR (101 MHz, DMSO): δ 176.1, 154.9, 143.0, 130.9, 130.6, 128.7, 128.5, 127.1, 126.9, 125.3, 62.9, 42.4. NMR spectrum is in agreement with the data reported in the literature. MS (ESI) calculated for C_16_H_16_N_3_S^+^ (M + H) 282.11 found 282.22. HRMS *m*/*z* calculated for C_16_H_16_N_3_S^+^ [M + H] 282.1059, found 282.1060.

##### 4.1.5.2 3-Phenyl-5-(*p*-tolyl)-4,5-dihydro-1*H*-pyrazole-1-carbothioamide (**33**)^57^

Following the general method E using chalcone (**10**), compound **33** was obtained as a white solid in 42% yield. *R*_f_ = 0.24 (PE/AcOEt 8:2). ^1^H NMR (400 MHz, DMSO): δ 7.97–7.94 (m, 4H), 7.46 (s, 3H), 7.06 (d, 4H, *J* = 12.4 Hz), 5.87 (d, 1H, *J* = 11.8 Hz), 3.88 (dd, 1H, *J* = 17.3, 11.8 Hz), 3.10 (d, 1H, *J* = 20.8 Hz), 2.25 (s, 3H). ^13^C NMR (101 MHz, DMSO): δ 176.1, 154.9, 140.1, 136.0, 130.9, 130.6, 129.0, 128.7, 127.1, 125.3, 62.6, 42.4, 20.7. NMR spectrum is in agreement with the data reported in the literature. MS (ESI) calculated for C_17_H_18_N_3_S^+^ (M + H) 296.12 found 296.14.

HRMS *m*/*z* calculated for C_17_H_18_N_3_S^+^ [M + H] 296.1216, found 296.1216.

##### 4.1.5.3 5-(4-Chlorophenyl)-3-phenyl-4,5-dihydro-1*H*-pyrazole-1-carbothioamide (**34**)^57^

Following the general method E using chalcone (**11**), compound **34** was obtained as a white solid in 62% yield. *R*_f_ = 0.23 (PE/AcOEt 8:2). ^1^H NMR (400 MHz, DMSO): δ 8.07 (s, 1H), 7.94–7.86 (m, 3H), 7.46–7.44 (m, 3H), 7.38–7.36 (m, 2H), 7.16–7.14 (m, 2H), 5.92 (dd, 1H, *J* = 11.5, 3.4 Hz), 3.91 (dd, 1H, *J* = 18.1, 11.5 Hz), 3.15 (dd, 1H, *J* = 18.1, 3.5 Hz). ^13^C NMR (101 MHz, DMSO): δ 176.1, 154.9, 142.0, 131.4, 130.8, 130.6, 128.7, 128.5, 127.4, 127.1, 62.3, 42.0. NMR spectrum is in agreement with the data reported in the literature. MS (ESI) calculated for C_16_H_15_ClN_3_S^+^ (M + H) 316.07 found 316.04. HRMS *m*/*z* calculated for C_16_H_15_ClN_3_S^+^ [M + H] 316.0670, found 316.0669.

##### 4.1.5.4 5-(4-Fluorophenyl)-3-phenyl-4,5-dihydro-1*H*-pyrazole-1-carbothioamide (**35**)

Following the general method E using chalcone (**12**), compound **35** was obtained as a off-yellow solid in 48% yield. *R*_f_ = 0.22 (PE/AcOEt 8:2). ^1^H NMR (400 MHz, DMSO): δ 8.05 (s, 1H), 7.89–7.87 (m, 3H), 7.47–7.45 (m, 3H), 7.19–7.11 (m, 4H), 5.93 (dd, 1H, *J* = 11.4, 3.4 Hz), 3.90 (dd, 1H, *J* = 18.1, 11.5 Hz), 3.16 (dd, 1H, *J* = 18.1, 3.5 Hz). ^13^C NMR (101 MHz, DMSO): δ 176.1, 161.1 (d, *J* = 242.3 Hz), 154.9, 139.2, 130.8, 130.6, 128.7, 127.4 (*d*, *J* = 8.3 Hz), 127.1, 115.2 (d, *J* = 21.5 Hz), 62.2, 42.3. MS (ESI) calculated for C_16_H_15_FN_3_S^+^ (M + H) 300.10 found 300.05. HRMS *m*/*z* calculated for C_16_H_15_FN_3_S^+^ [M + H] 300.0965, found 300.0964.

##### 4.1.5.5 3-(2,4-Dimethoxyphenyl)-5-phenyl-4,5-dihydro-1*H*-pyrazole-1-carbothioamide (**36**)

Following the general method E using chalcone (**14**), compound **36** was obtained as a white solid in 40% yield. *R*_f_ = 0.47 (Pe/AcOEt 7:3). ^1^H NMR (400 MHz, DMSO): δ 8.04–8.02 (m, 1H), 7.88 (s, 1H), 7.71 (s, 1H), 7.32–7.28 (m, 2H), 7.22–7.19 (m, 1H), 7.12–7.10 (m, 2H), 6.61–6.59 (m, 2H), 5.83 (dd, 1H, *J* = 11.2, 3.0 Hz), 3.90 (dd, 1H, *J* = 18.7, 11.5 Hz), 3.12 (dd, 1H, *J* = 18.5, 3.1 Hz). ^13^C NMR (101 MHz, DMSO): δ 175.6, 162.7, 159.7, 154.2, 143.3, 130.3, 128.4, 126.8, 125.2, 112.1, 106.4, 98.5, 62.4, 55.7, 55.5, 45.8. MS (ESI) calculated for C_18_H_20_N_3_O_2_S^+^ (M + H) 342.13 found 342.13. HRMS *m*/*z* calculated for C_18_H_20_N_3_O_2_S^+^ [M + H] 342.1271, found 342.1271.

#### 4.1.6 General Method for the Synthesis of Dihydropyrimidine Thiones **37**–**41**, F

In a round-bottom flask, NaOH (68 mg, 1.70 mmol) is dissolved in absolute EtOH (3.4 mL) followed by the addition of the appropriate chalcone **9**–**12**, **14**, and thiourea (129 mg, 1.70 mmol). The mixture was sonicated at room temperature for 1 h. The content was poured into ice water, left to stand for 24 h, and the solid was filtered, washed with H_2_O and then with a mixture of EtOH/H_2_O 1:1, and left to stand over P_2_O_5_.

##### 4.1.6.1 4,6-Diphenyl-3,4-dihydropyrimidine-2(1*H*)-thione (**37**)^61^

Following the general method F using chalcone (**9**), compound **37** was obtained as an off-yellow solid in 97% yield. *R*_f_ = 0.71 (PE/AcOEt 7:3). ^1^H NMR (400 MHz, CDCl_3_): δ 7.87 (s, 1H), 7.44–7.32 (m, 10H), 7.27–7.25 (m, 1H), 5.29–5.25 (m, 1H), 5.20–5.17 (m, 1H). ^13^C NMR (101 MHz, CDCl_3_): δ 175.1, 142.4, 134.1, 133.3, 129.6, 129.2, 129.1, 128.7, 127.0, 125.4, 100.8, 57.2. NMR spectrum is in agreement with the data reported in the literature. MS (ESI) calculated for C_16_H_15_N_2_S^+^ (M + H) 267.10 found 267.13. HRMS *m*/*z* calculated for C_16_H_15_N_2_S^+^ [M + H] 267.0950, found 267.0951.

##### 4.1.6.2 6-Phenyl-4-(*p*-tolyl)-3,4-dihydropyrimidine-2(1*H*)-thione (**38**)^62^

Following the general method F using chalcone (**10**), compound **38** was obtained as a beige solid in 72% yield. *R*_f_ = 0.62 (PE/AcOEt 8:2). ^1^H NMR (400 MHz, DMSO): δ 9.79 (s, 1H), 9.06–9.04 (m, 1H), 7.52–7.50 (m, 2H), 7.37–7.35 (m, 3H), 7.22–7.20 (m, 4H), 5.36–5.34 (m, 1H), 5.08–5.06 (m, 1H), 2.29 (s, 3H). ^13^C NMR (101 MHz, DMSO): δ 175.0, 141.2, 136.8, 134.3, 133.4, 129.2, 128.8, 128.4, 126.4, 125.9, 101.3, 54.4, 20.7. NMR spectrum is in agreement with the data reported in the literature. MS (ESI) calculated for C_17_H_17_N_2_S^+^ (M + H) 281.11 found 281.17. HRMS *m*/*z* calculated for C_17_H_17_N_2_S^+^ [M + H] 281.1107, found 281.1109.

##### 4.1.6.3 4-(4-Chlorophenyl)-6-phenyl-3,4-dihydropyrimidine-2(1*H*)-thione (**39**)^62^

Following the general method F using chalcone (**11**), compound **39** was obtained as a beige solid in 45% yield. *R*_f_ = 0.56 (PE/AcOEt 8:2). ^1^H NMR (400 MHz, DMSO): δ 9.89 (s, 1H), 9.13–9.11 (m, 1H), 7.50–7.46 (m, 4H), 7.36–7.34 (m, 5H), 5.39–5.37 (m, 1H), 5.15–5.13 (m, 1H). ^13^C NMR (101 MHz, DMSO): δ 175.2, 143.0, 134.6, 133.2, 132.1, 128.9, 128.7, 128.4, 128.2, 125.9, 100.7, 53.8. NMR spectrum is in agreement with the data reported in the literature. MS (ESI) calculated for C_16_H_14_ClN_2_S^+^ (M + H) 301.06 found 301.02. HRMS *m*/*z* calculated for C_16_H_14_ClN_2_S^+^ [M + H] 301.0561, found 301.0560.

##### 4.1.6.4 4-(4-Fluorophenyl)-6-phenyl-3,4-dihydropyrimidine-2(1*H*)-thione (**40**)

Following the general method F using chalcone (**12**), compound **40** was obtained as an off-orange solid in 64% yield. *R*_f_ = 0.57 (PE/AcOEt 8:2). ^1^H NMR (400 MHz, DMSO): δ 9.87 (s, 1H), 9.12–9.10 (m, 1H), 7.52–7.50 (m, 2H), 7.38–7.36 (m, 5H), 7.25–7.23 (m, 2H), 5.39–5.37 (m, 1H), 5.15–5.13 (m, 1H). ^13^C NMR (101 MHz, DMSO): δ 175.1, 161.5 (d, *J* = 243.5 Hz), 140.3, 140.3, 134.5, 133.2, 128.7 (d, *J* = 37.6 Hz), 128.4, 128.4, 125.9, 115.4 (d, *J* = 21.4 Hz), 100.9, 53.8. MS (ESI) calculated for C_16_H_14_FN_2_S^+^ (M + H) 285.09 found 285.13. HRMS *m*/*z* calculated for C_16_H_14_FN_2_S^+^ [M + H] 285.0856, found 285.0858.

##### 4.1.6.5 6-(2,4-Dimethoxyphenyl)-4-phenyl-3,4-dihydropyrimidine-2(1*H*)-thione (**41**)

Following the general method F using chalcone (**14**), compound **41** was obtained as an off-orange solid in 96% yield. *R*_f_ = 0.76 (PE/AcOEt 8:2). ^1^H NMR (400 MHz, DMSO): δ 9.30 (s, 1H), 8.94–8.92 (m, 1H), 7.42–7.35 (m, 4H), 7.32–7.28 (m, 1H), 7.15 (d, 1H, *J* = 8.4 Hz), 6.58–6.57 (m, 1H), 6.52–6.49 (m, 1H), 5.08–5.06 (m, 1H), 5.05–5.03 (m, 1H), 3.79 (s, 3H), 3.77 (s, 3H). ^13^C NMR (101 MHz, DMSO): δ 174.0, 161.2, 158.1, 144.3, 132.7, 130.3, 128.6, 127.5, 126.6, 115.6, 104.8, 101.6, 98.7, 55.7, 55.3, 54.8. MS (ESI) calculated for C_18_H_19_N_2_O_2_S^+^ (M + H) 327.12 found 327.00. HRMS *m*/*z* calculated for C_18_H_19_N_2_O_2_S^+^ [M + H] 327.1162, found 327.1160.

#### 4.1.7 General Method for the Synthesis of Aminothiazoles **44**–**47**, G

The appropriate acetophenone **5**, **6**, **8**, **42**, **43** (0.02 mol), thiourea (3.04 g, 0.04 mol), and I_2_ (5.08 g, 0.02 mol) are heated together at 100 °C overnight. The reaction mixture is diluted with AcOEt (300 mL) and H_2_O (20 mL) and neutralized with aq. NH_3_ 25% to pH = 9–10. The mixture is transferred to a separatory funnel, and the organic layer is collected, washed with H_2_O (20 mL), dried over Na_2_SO_4_, and concentrated *in vacuo*. The crude material was purified by column chromatography using CH_2_Cl_2_/MeOH 9.5:0.5 as an eluent.

##### 4.1.7.1 4-Phenylthiazol-2-amine (**44**)^63^

Following the general method G, using acetophenone (**5**), thiazole **44** was obtained as a yellowish solid in 87% yield (3.06 g). *R*_f_ = 0.65 (CH_2_Cl_2_/MeOH 9.5:0.5). ^1^H NMR (400 MHz, DMSO): δ 7.78 (d, *J* = 7.9 Hz, 2H), 7.35 (t, *J* = 7.5 Hz, 2H), 7.25 (t, *J* = 7.3 Hz, 1H), 7.03 (s, 2H), 6.98 (s, 1H). ^13^C NMR (101 MHz, DMSO): δ 168.2, 149.9, 134.9, 128.4, 127.2, 125.5, 101.5. MS (ESI) *m*/*z* calculated for C_9_H_9_N_2_S^+^ [M + H] 177.24 found 177.24.

##### 4.1.7.2 4-(4-Methoxyphenyl)thiazol-2-amine (**45**)^63^

Following the general method G, using 4′-methoxyacetophenone (**42**), thiazole **45** was obtained as a yellowish solid in 65% yield (2.70 g). *R*_f_ = 0.56 (CH_2_Cl_2_/MeOH 9.5:0.5). ^1^H NMR (400 MHz, DMSO): δ 7.73 (d, *J* = 7.6 Hz, 2H), 7.00 (s, 2H), 6.92 (d, *J* = 7.8 Hz, 2H), 6.80 (s, 1H), 3.76 (s, 3H). ^13^C NMR (101 MHz, DMSO): δ 168.1, 158.5, 149.7, 127.9, 126.9, 113.8, 99.34, 55.07. MS (ESI) *m*/*z* calculated for C_10_H_11_N_2_OS^+^ [M + H] 207.06, found 207.07.

##### 4.1.7.3 4-(2,4-Dimethoxyphenyl)thiazol-2-amine (**46**)^63^

Following the general method G, using 2,4-dimethoxyacetophenone (**8**), thiazole **46** was obtained as a pale orange solid in 47% yield (2.21 g). *R*_f_ = 0.53 (CH_2_Cl_2_/MeOH 9.5:0.5). ^1^H NMR (400 MHz, CDCl_3_): δ 7.96 (d, *J* = 8.5 Hz, 1H), 7.04 (s, 1H), 6.55 (d, *J* = 2.3 Hz, 1H), 6.52 (t, *J* = 2.9, 1H), 5.08 (s, 2H), 3.89 (s, 3H), 3.83 (s, 3H). ^13^C NMR (101 MHz, CDCl_3_): δ 165.6, 160.2, 158.1, 146.9, 130.8, 117.0, 105.7, 104.6, 98.93, 55.54, 55.51. MS (ESI) *m*/*z* calculated for C_11_H_13_N_2_O_2_S^+^ [M + H] 237.12, found 237.13.

##### 4.1.7.4 4-(2-Aminothiazol-4-yl)phenol (**47**)^63^

Following the general method G, using 4-hydoxyacetophenone (**6**), thiazole **47** was obtained as an off-white solid in 86% yield (3.29 g). *R*_f_ = 0.32 (CH_2_Cl_2_/MeOH 9.5:0.5). ^1^H NMR (400 MHz, DMSO): δ 9.44 (s, 1H), 7.61 (d, *J* = 7.6 Hz, 2H), 6.94 (s, 2H), 6.76 (d, *J* = 7.9, 2H), 6.70 (s, 1H). ^13^C NMR (101 MHz, DMSO): δ 168.0, 156.8, 150.1, 126.9, 126.4, 115.2, 98.46. MS (ESI) *m*/*z* calculated for C_9_H_9_N_2_OS^+^ [M + H] 193.06, found 193.05.

##### 4.1.7.5 4-(4-Bromophenyl)thiazol-2-amine (**48**)^64^

A solution of 4-bromophenacyl bromide **43** (0.02 mol) and thiourea (3.04 g, 0.04 mol) in absolute EtOH (70 mL) is refluxed overnight. The reaction mixture is concentrated *in vacuo*, AcOEt (300 mL) and H_2_O (20 mL) were added, and the mixture is neutralized with aq. NH_3_ 25% to pH = 9–10. The organic layer was collected, washed with H_2_O (20 mL), dried over Na_2_SO_4_, and concentrated *in vacuo*. The crude material is purified by column chromatography using CH_2_Cl_2_/MeOH 9.5:0.5 as eluent, giving thiazole **48** as a light orange solid in 89% yield (4.55 g). *R*_f_ = 0.69 (CH_2_Cl_2_/MeOH 9.5:0.5). ^1^H NMR (400 MHz, DMSO): δ 7.73 (d, *J* = 8.5 Hz, 2H), 7.55 (d, *J* = 8.5 Hz, 2H), 7.08 (s, 2H), 7.07 (s, 1H). ^13^C NMR (101 MHz, DMSO): δ 168.3, 148.6, 134.1, 131.3, 127.5, 120.1, 102.4. MS (ESI) *m*/*z* calculated for C_9_H_8_BrN_2_S^+^ [M + H] 255.06, found 255.07

#### 4.1.8 General Method for the Synthesis of Thiazolyl Thioureas **49**–**53**, H

To a stirred solution of thiazoles **44**–**48** (0.5 mmol) in dry DMF (2 mL) benzyl isothiocyanate (0.20 mL, 1.5 mmol) was added and the reaction mixture was left to stand at 70 °C for 2 days. Then, the reaction mixture was partitioned between CH_2_Cl_2_ and H_2_O, the organic phase was dried over Na_2_SO_4_, and the residue was purified by column chromatography using an appropriate mixture of CH_2_Cl_2_/MeOH as an eluent to give thiazolyl thioureas **49**–**53**.

##### 4.1.8.1 1-Benzyl-3-(4-phenylthiazol-2-yl)thiourea (**49**)

Following the general method H using thiazole **44**, thiazolyl thiourea **49** was obtained as a white solid in 63% yield. *R*_f_ = 0.70 (CH_2_Cl_2_/MeOH 9.9:0.1). ^1^H NMR (400 MHz, DMSO): δ 11.77 (s, 1H), 10.29–9.95 (m, 1H), 7.67 (d, *J* = 7.0 Hz, 2H), 7.53 (s, 1H), 7.48–7.38 (m, 4H), 7.38–7.24 (m, 4H), 4.79 (d, *J* = 4.57 Hz, 2H). ^13^C NMR (101 MHz, DMSO): δ 177.7, 161.2, 148.5, 137.5, 133.5, 128.6, 127.8, 127.5, 125.5, 106.7, 48.06. MS (ESI) *m*/*z* calculated for C_17_H_16_N_3_S_2_^+^ [M + H] 326.10, found 326.15. HRMS *m*/*z* calculated for C_17_H_16_N_3_S_2_^+^ [M + H] 326.0780, found 326.0781.

##### 4.1.8.2 1-Benzyl-3-(4-(4-methoxyphenyl)thiazol-2-yl)thiourea (**50**)

Following the general method H using thiazole **45**, thiazolyl thiourea **50** was obtained as a white solid in 94% yield. *R*_f_ = 0.71 (CH_2_Cl_2_/MeOH 9.9:0.1). ^1^H NMR (400 MHz, DMSO): δ 11.69 (s, 1H), 10.28–10.05 (m, 1H), 7.61 (d, *J* = 6.6 Hz, 2H), 7.49–7.38 (m, 5H), 7.34 (s, 1H), 6.90 (d, *J* = 7.0 Hz, 2H) 4.83–4.76 (m, 2H), 3.78 (s, 3H). ^13^C NMR (101 MHz, DMSO): δ 177.7, 161.0, 159.0, 148.4, 137.5, 128.6, 127.8, 127.4, 126.8, 126.4, 114.0, 104.6, 55.10, 48.01. MS (ESI) *m*/*z* calculated for C_18_H_18_N_3_OS_2_^+^ [M + H] 356.09, found 356.08. HRMS *m*/*z* calculated for C_18_H_18_N_3_OS_2_^+^ [M + H] 356.0886, found 356.0886.

##### 4.1.8.3 1-Benzyl-3-(4-(2,4-dimethoxyphenyl)thiazol-2-yl)thiourea (**51**)

Following the general method H using thiazole **46**, thiazolyl thiourea **51** was obtained as a light-yellow solid in 81% yield. *R*_f_ = 0.71 (CH_2_Cl_2_/MeOH 9.9:0.1). ^1^H NMR (400 MHz, DMSO): δ 11.68 (s, 1H), 10.45–10.23 (m, 1H), 7.47 (d, *J* = 7.0 Hz, 1H), 7.46–7.39 (m, 5H), 7.38 (s, 1H), 6.63 (s, 1H), 6.42 (d, *J* = 6.9 Hz, 1H), 4.84–4.76 (m, 2H), 3.87 (s, 3H), 3.79 (s, 3H). ^13^C NMR (101 MHz, DMSO): δ 177.7, 160.0, 159.6, 157.7, 144.6, 137.5, 129.4, 128.6, 127.9, 127.5, 115.0, 108.0, 105.2, 98.55, 55.51, 55.22, 48.12. MS (ESI) *m*/*z* calculated for C_19_H_20_N_3_O_2_S_2_^+^ [M + H] 386.11, found 386.47. HRMS *m*/*z* calculated for C_19_H_20_N_3_O_2_S_2_^+^ [M + H] 386.0991, found 386.0992.

##### 4.1.8.4 1-Benzyl-3-(4-(4-hydroxyphenyl)thiazol-2-yl)thiourea (**52**)

Following the general method H using thiazole **47**, thiazolyl thiourea **51** was obtained as a light-yellow solid in 82% yield. *R*_f_ = 0.29 (CH_2_Cl_2_/MeOH 9.9:0.1). ^1^H NMR (400 MHz, DMSO): δ 11.71 (s, 1H), 10.46–10.20 (m, 1H), 9.57 (s, 1H), 7.53–7.31 (m, 7H), 7.25 (s, 1H), 6.72 (d, *J* = 6.84 Hz, 2H), 4.83–4.74 (m, 2H). ^13^C NMR (101 MHz, DMSO): δ 177.6, 161.0, 157.3, 148.8, 137.5, 128.6, 127.9, 127.5, 126.9, 124.8, 115.4, 103.6, 48.15. MS (ESI) *m*/*z* calculated for C_17_H_16_N_3_OS_2_^+^ [M + H] 342.08, found 341.78. HRMS *m*/*z* calculated for C_17_H_16_N_3_OS_2_^+^ [M + H] 342.0729, found 342.0727.

##### 4.1.8.5 1-Benzyl-3-(4-(4-bromophenyl)thiazol-2-yl)thiourea (**53**)

Following the general method H using thiazole **48**, thiazolyl thiourea **53** was obtained as a white solid in 90% yield. *R*_f_ = 0.69 (CH_2_Cl_2_/MeOH 9.9:0.1). ^1^H NMR (400 MHz, DMSO): δ 11.76 (s, 1H), 10.10–9.61 (m, 1H), 7.64 (d, *J* = 6.7 Hz, 2H), 7.59 (s, 1H), 7.54 (d, *J* = 7.1 Hz, 2H), 7.48–7.30 (m, 5H), 4.84–4.73 (m, 2H). ^13^C NMR (101 MHz, DMSO): δ 177.7, 161.3, 147.3, 137.6, 132.9, 131.5, 128.6, 127.8, 127.5, 120.9, 107.6, 47.92. MS (ESI) *m*/*z* calculated for C_17_H_15_BrN_3_S_2_^+^ [M + H] 404.00, found 404.03. HRMS *m*/*z* calculated for C_17_H_15_BrN_3_S_2_^+^ [M + H] calculated for 403.9885, found 403.9883.

### 4.2 Molecular Modeling

The crystal structure of *S. pasteurii* urease measured with 1.50 Å resolution, (PDB id 5G4H) was used as the starting point for the calculations.^44^

Modeling studies were performed using BIOVIA Discovery Studio 4.1. The structure was prepared for the calculations in the following steps: (a) the hydrogen atoms were automatically added assuming a pH of 7.0, (b) the protonation of amino acid residues that form active sites was manually checked and adjusted, and (c) the partial charges of all atoms were assigned using the Momany–Rone algorithm. Minimization of the inhibitor–enzyme complex was done using the CHARMM forcefield with Smart Minimizer algorithm. Minimization was performed up to an energy change of 0.0 or an RMS gradient of 0.1. Residues that did not form the active site cleft were frozen. No implicit solvent model was applied. The nonbond radius was set to 14 Å.

### 4.3 Enzyme Purification

The *S. pasteurii* CCM 2056 cells were used as the source of a native enzyme. The culture medium was made of 20 g/L yeast extract. One day after the inoculation, the medium was further supplemented with 1 mM NiCl_2_ and 1% urea. After 2 more days of growth, the cells were centrifuged and disintegrated via sonication. Enzyme purification from the cell extract was conducted using a five-step chromatographic method as previously reported.^50^

### 4.4 Enzyme Inhibition Studies

Inhibition of the purified urease was measured using the indophenol assay for ammonia concentration.^65^ The kinetic assays in 3 mM phosphate buffer contained the purified enzyme, studied thiourea analogues, and a range of urea concentrations up to saturating 30 mM. Total progress curves were analyzed with the addition of the enzyme at the reaction start. Assays were terminated with the sequential addition of 100 μL of M1 (0.955 μM sodium nitroprusside, 0.53 M phenol) and M2 (0.625 M sodium hydroxide, 47.1 mM sodium hypochlorite) solution to 50 μL of reaction mixture sample. After 15 min of color development, absorbance at 650 nm was measured. IC_50_ value was calculated using GraphPad Prism 5 software for data obtained at 30 mM urea. In order to verify the inhibition mode as well as calculate the dissociation constant for EI complex, Lineweaver–Burke curves were plotted (1/*v* = *f*(1/[*S*])). For noncompetitive or mixed inhibition mode, *K*_*i*_ was calculated from plots of the LB curves slope as a function of inhibitor concentration.

### 4.5 Whole Cells Ureolysis

*Proteus mirabilis* PCM 543 was cultured as described for *S. pasteurii* except for nickel ions—0.3 mM NiCl_2_ was used in the growth medium. Cells harvested by centrifugation were resuspended in 10 mM PBS and used for urease activity measurements. Assays were conducted in 10 mM PBS containing 30 mM urea and different concentrations of urease inhibitors and ammonia quantified by indophenol assay.

## Supporting Information Available

^1^H, ^13^C NMR, and ESI MS spectra and kinetic characteristics of compounds (PDF)
