# Supplementary material for: Exploration of Thiourea-Based Scaffolds for the Construction of Bacterial Ureases Inhibitors
Source: ACS Omega. 2023 Jul 28;8(31):28783–96. doi: 10.1021/acsomega.3c03702 (PMC10413841; doi:10.1021/acsomega.3c03702)
Supplement: Supplementary file 1 — ao3c03702_si_001.pdf [file ao3c03702_si_001.pdf]

Supporting Information for Publication

Exploration of Thiourea-Based Scaffolds for the Construction of Bacterial Ureases Inhibitors

Wojciech Tabor,[a] Aikaterini Katsogiannou,[b] Danai Karta,[b] Evgenia Andrianopoulou,[b] Łukasz Berlicki,[a] Stamatia Vassiliou,[b]* Agnieszka Grabowiecka [a]*

[a] Department of Bioorganic Chemistry, Faculty of Chemistry, Wrocław University of Science and Technology, Wybrzeże Wyspiańskiego 27, 50-370 Wrocław, Poland

[b] Laboratory of Organic Chemistry, Department of Chemistry, University of Athens, Panepistimiopolis, Zografou, 15771 Athens, Greece

* corresponding authors: Agnieszka Grabowiecka agnieszka.grabowiecka@pwr.edu.pl Stamatia Vassiliou svassiliou@chem.uoa.gr

Table of contents

Figure SI_1 – 9	^{1}H NMR for compounds **15-23**
Figure SI_10 – 12	^{1}H, ^{13}C NMR, ESI MS for compound **24**
Figure SI_13 – 15	^{1}H, ^{13}C NMR, ESI MS for compound **25**
Figure SI_16 – 18	^{1}H, ^{13}C NMR, ESI MS for compound **26**
Figure SI_19 – 20	^{1}H NMR for compounds **27-28**
Figure SI_21 – 23	^{1}H, ^{13}C NMR, ESI MS for compound **29**
Figure SI_24 – 26	^{1}H, ^{13}C NMR, ESI MS for compound **30**
Figure SI_27– 29	^{1}H, ^{13}C NMR, ESI MS for compound **31**
Figure SI_30 – 32	^{1}H NMR for compounds **32-34**
Figure SI_33 – 35	^{1}H, ^{13}C NMR, ESI MS for compound **35**
Figure SI_36 – 38	^{1}H, ^{13}C NMR, ESI MS for compound **36**
Figure SI_39 – 41	^{1}H NMR for compounds **37-39**
Figure SI_42 – 44	^{1}H, ^{13}C NMR, ESI MS for compound **40**
Figure SI_45 – 47	^{1}H, ^{13}C NMR, ESI MS for compound **41**
Figure SI_48 – 50	^{1}H, ^{13}C NMR, ESI MS for compound **49**
Figure SI_51 – 53	^{1}H, ^{13}C NMR, ESI MS for compound **50**
Figure SI_54 – 56	^{1}H, ^{13}C NMR, ESI MS for compound **51**
Figure SI_57 – 59	^{1}H, ^{13}C NMR, ESI MS for compound **52**
Figure SI_60 – 62	^{1}H, ^{13}C NMR, ESI MS for compound **53**
Table SI_1	Kinetic characteristics of compounds

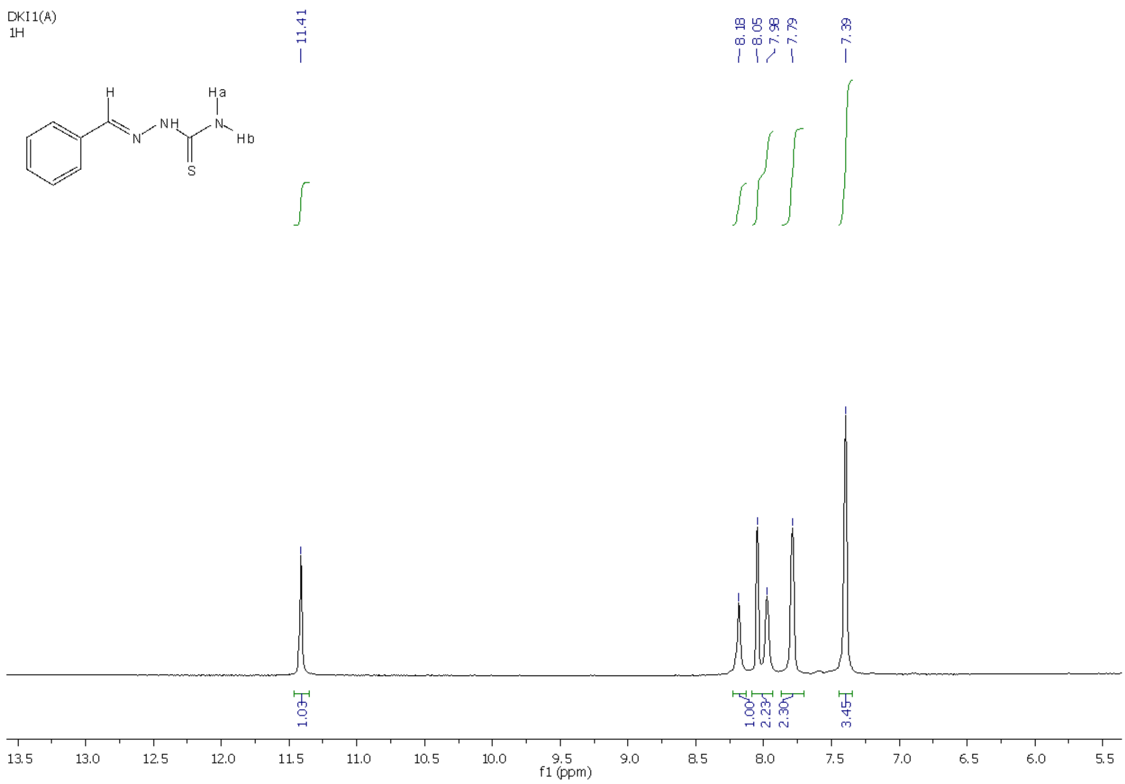

Figure SI_1. ^{1}H NMR of compound **15** ((*E*)-2-benzylidenehydrazine-1-carbothioamide) in DMSO

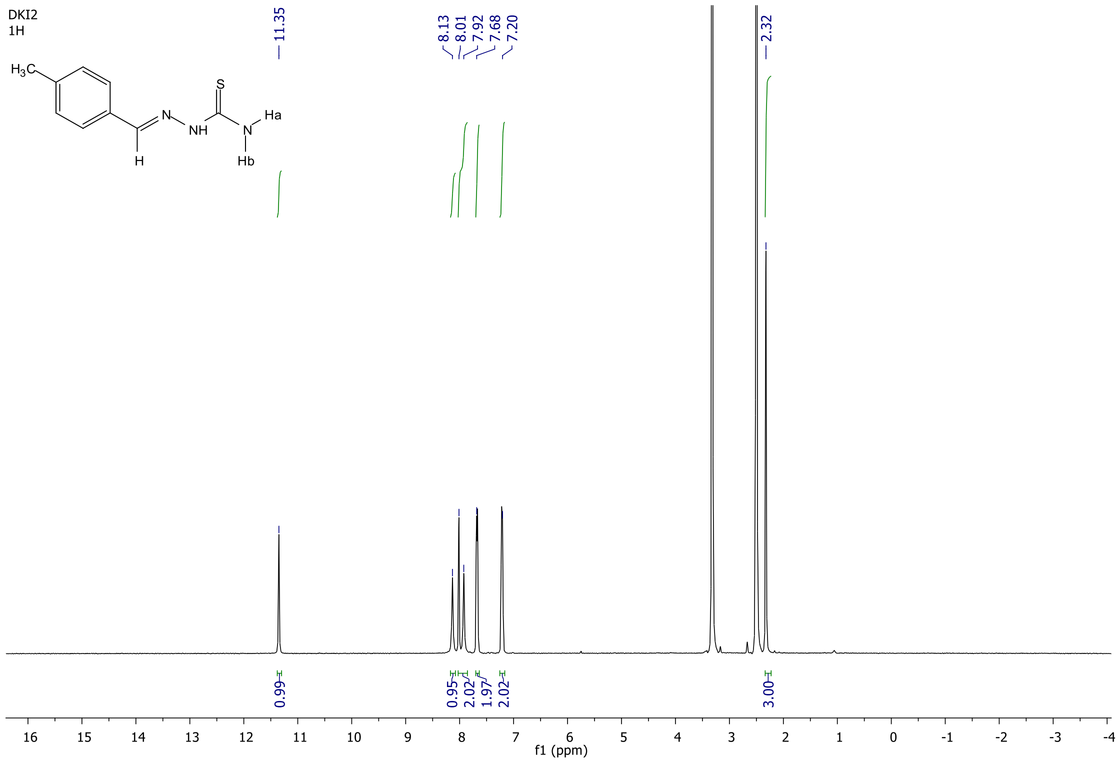

Figure SI_2. ^{1}H NMR of compound **16** ((*E*)-2-(4-methylbenzylidene)hydrazine-1-carbothioamide) in DMSO

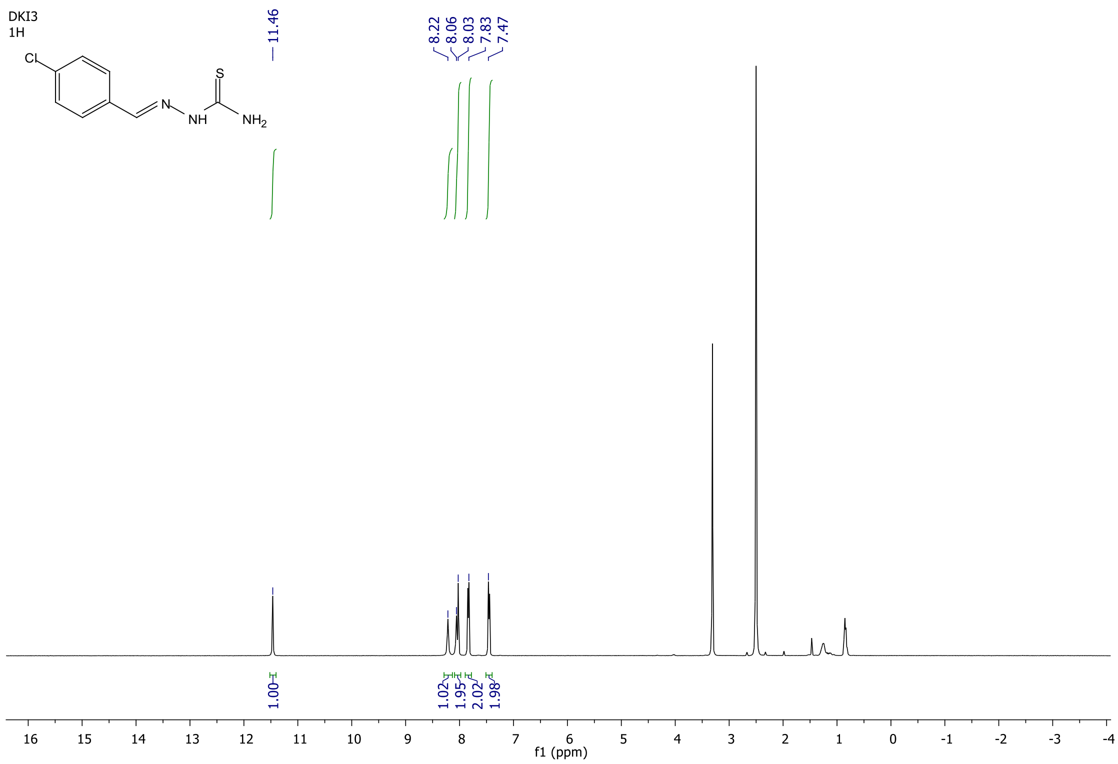

Figure SI_3. ^{1}H NMR of compound **17** ((*E*)-2-(4-chlorobenzylidene)hydrazine-1-carbothioamide) in DMSO

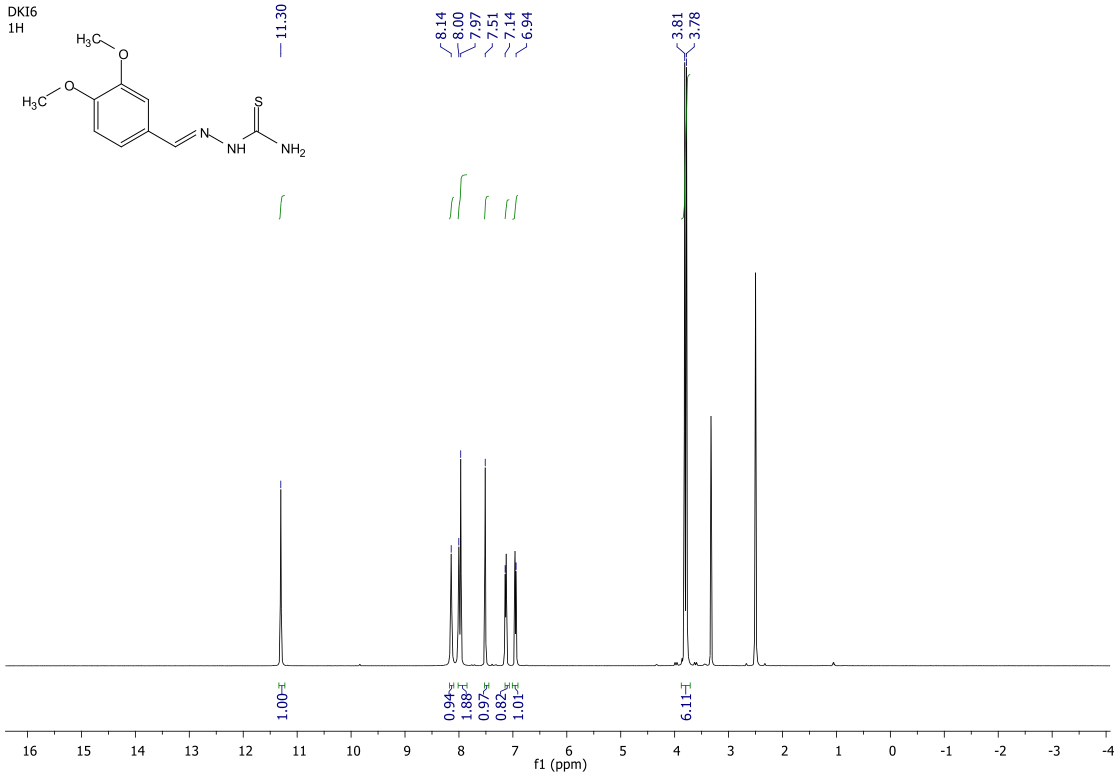

Figure SI_4. ^{1}H NMR of compound **18** ((*E*)-2-(3,4-dimethoxybenzylidene)hydrazine-1-carbothioamide) in DMSO

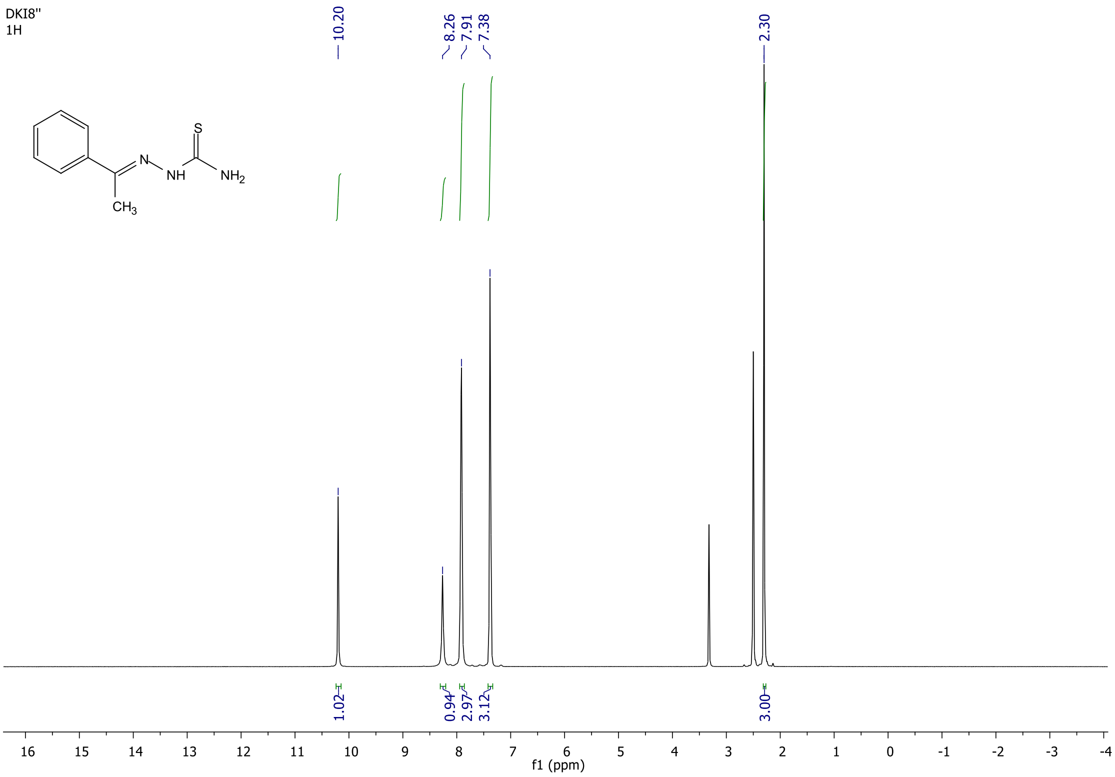

Figure SI_5. ^{1}H NMR of compound **19** ((*E*)-2-(1-phenylethylidene)hydrazine-1-carbothioamide) in DMSO

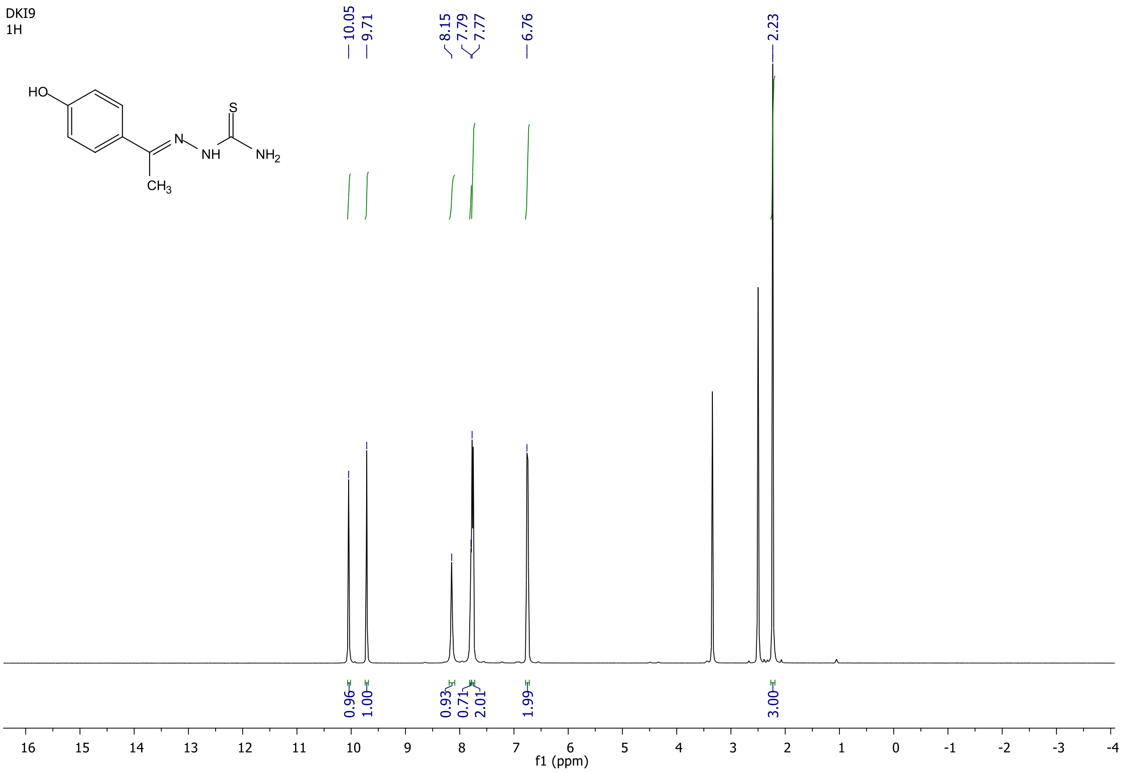

Figure SI_6. ^{1}H NMR of compound **20** ((*E*)-2-(1-(4-hydroxyphenyl)ethylidene)hydrazine-1-carbothioamide) in DMSO

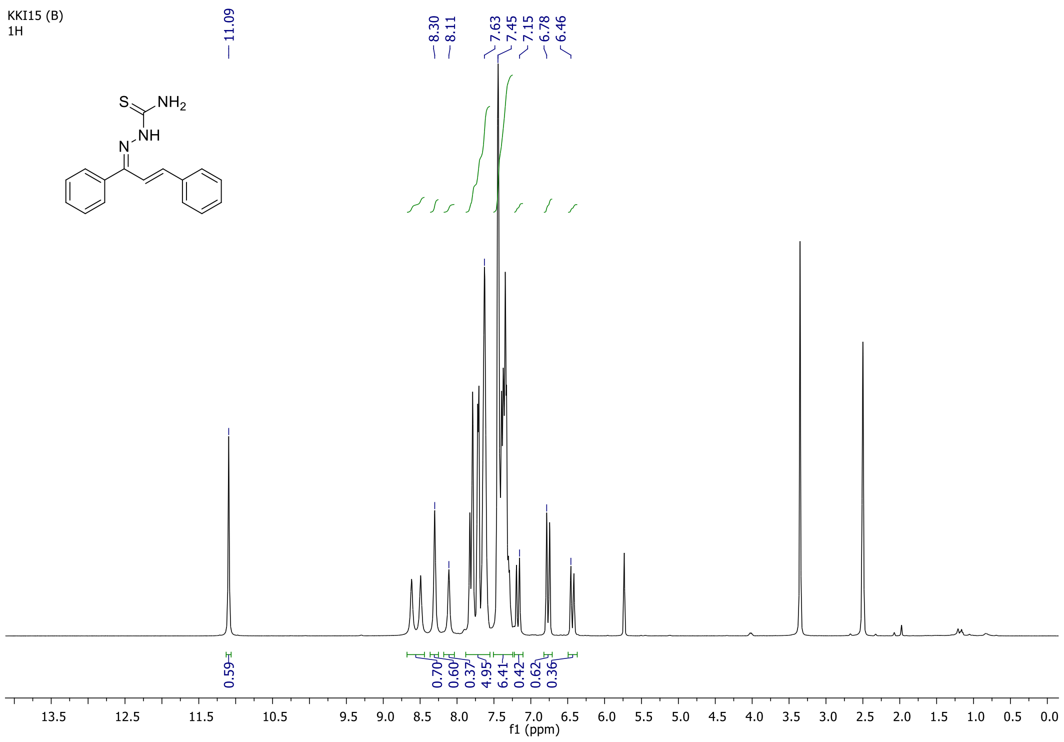

Figure SI_7. ^{1}H NMR of compound **21** ((*E*)-2-((*E*)-1,3-diphenylallylidene)hydrazine-1-carbothioamide) in DMSO

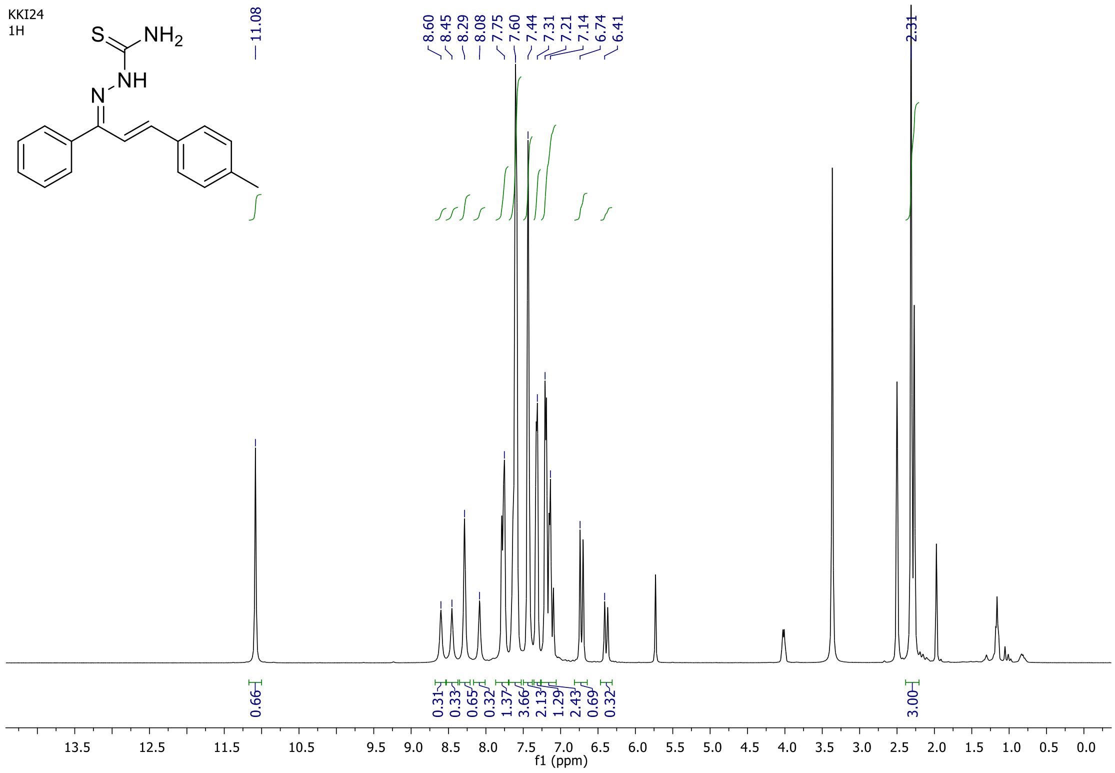

Figure SI_8. ^{1}H NMR of compound **22** ((*E*)-2-((*E*)-1-phenyl-3-(p-tolyl)allylidene)hydrazine-1-carbothioamide) in DMSO

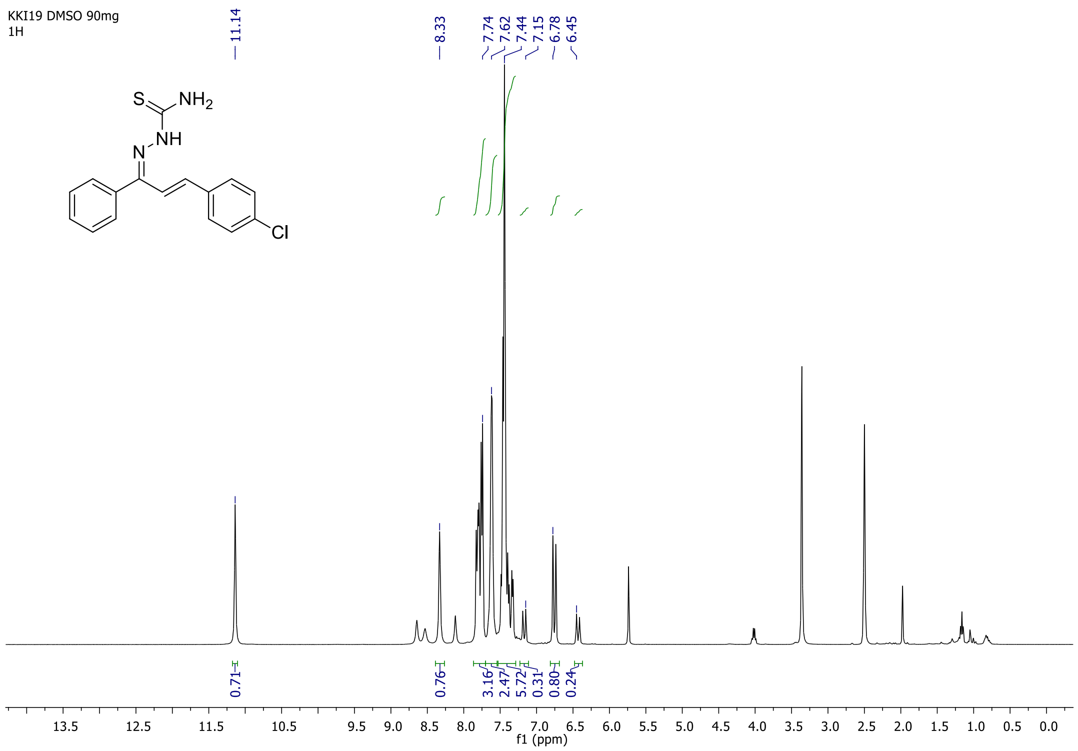

Figure SI_9. ^{1}H NMR of compound **23** ((*E*)-2-((*E*)-3-(4-chlorophenyl)-1-phenylallylidene)hydrazine-1-carbothioamide) in DMSO

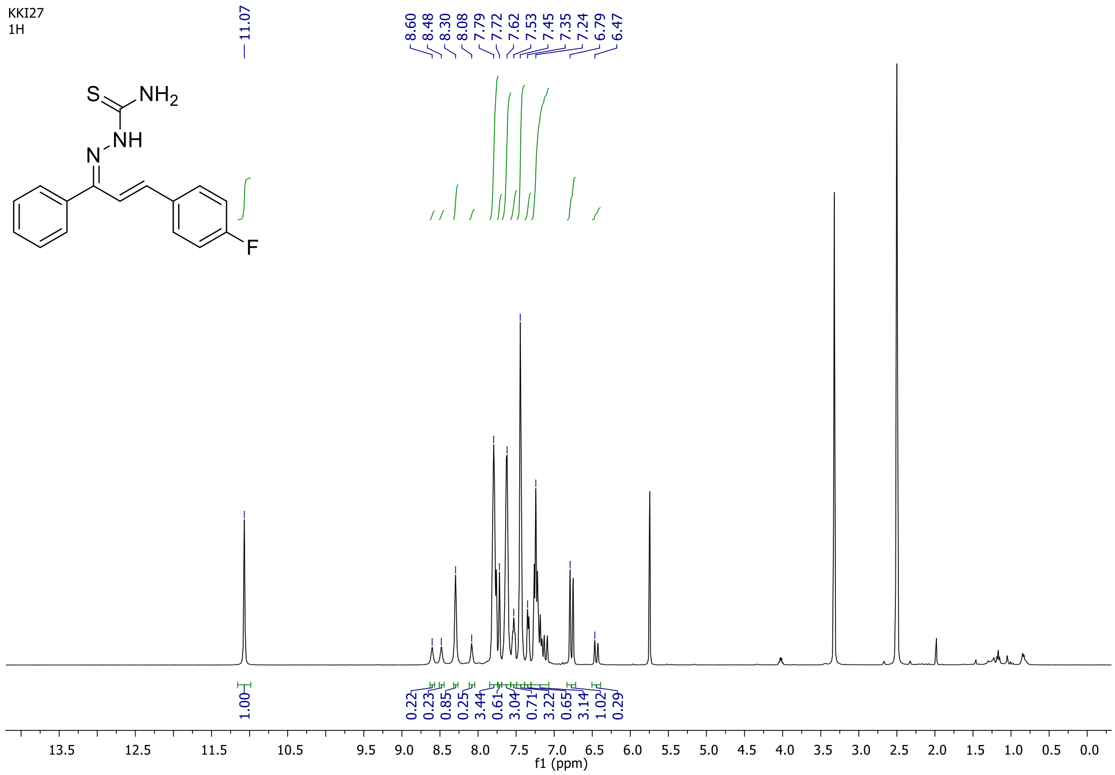

Figure SI_10. ^{1}H NMR of compound **24** ((*E*)-2-((*E*)-3-(4-fluorophenyl)-1-phenylallylidene)hydrazine-1-carbothioamide) in DMSO

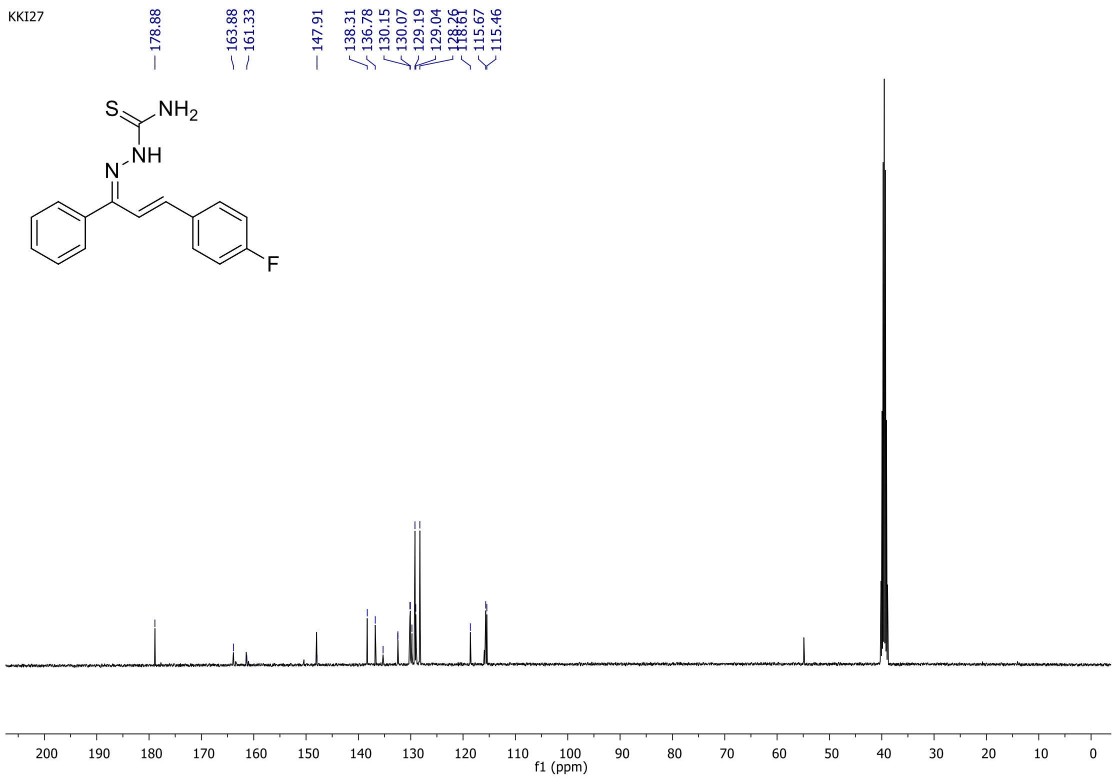

Figure SI_11. ^{13}C NMR of compound **24** ((*E*)-2-((*E*)-3-(4-fluorophenyl)-1-phenylallylidene)hydrazine-1-carbothioamide) in DMSO

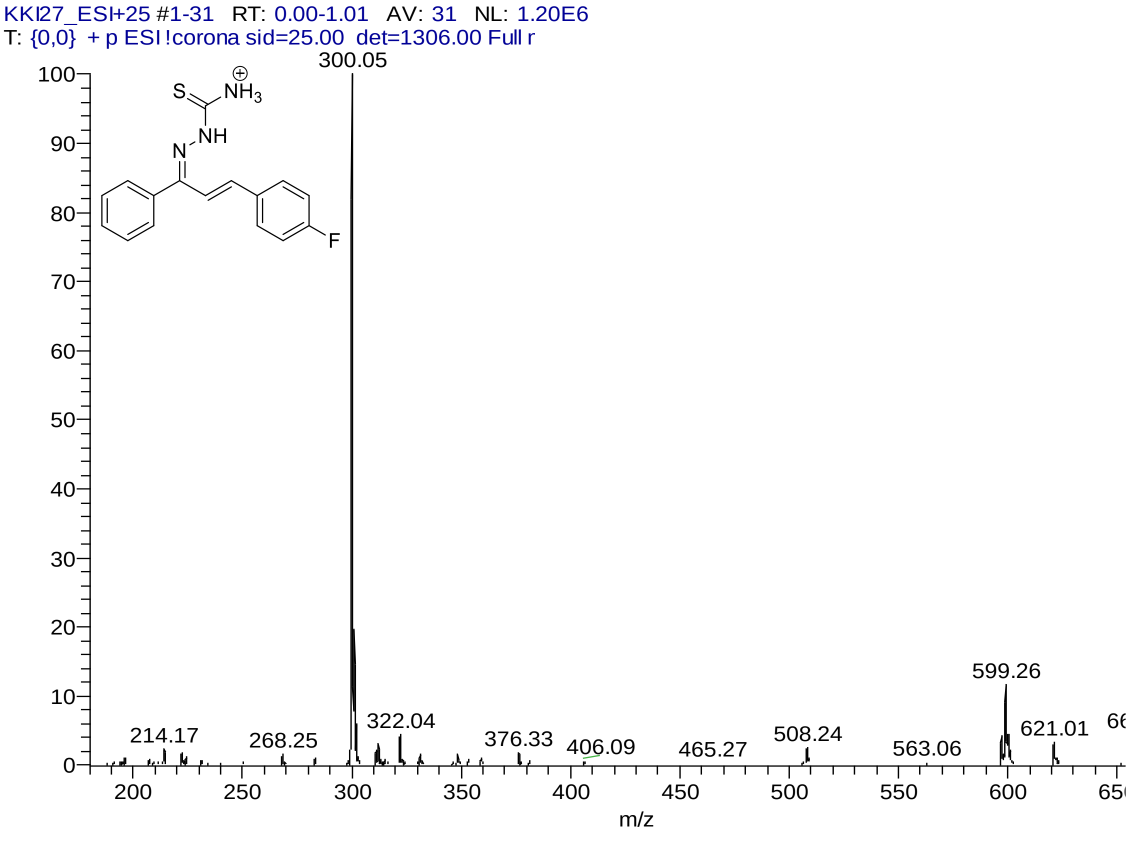

Figure SI_12. ESI MS of compound **24** ((*E*)-2-((*E*)-3-(4-fluorophenyl)-1-phenylallylidene)hydrazine-1-carbothioamide)

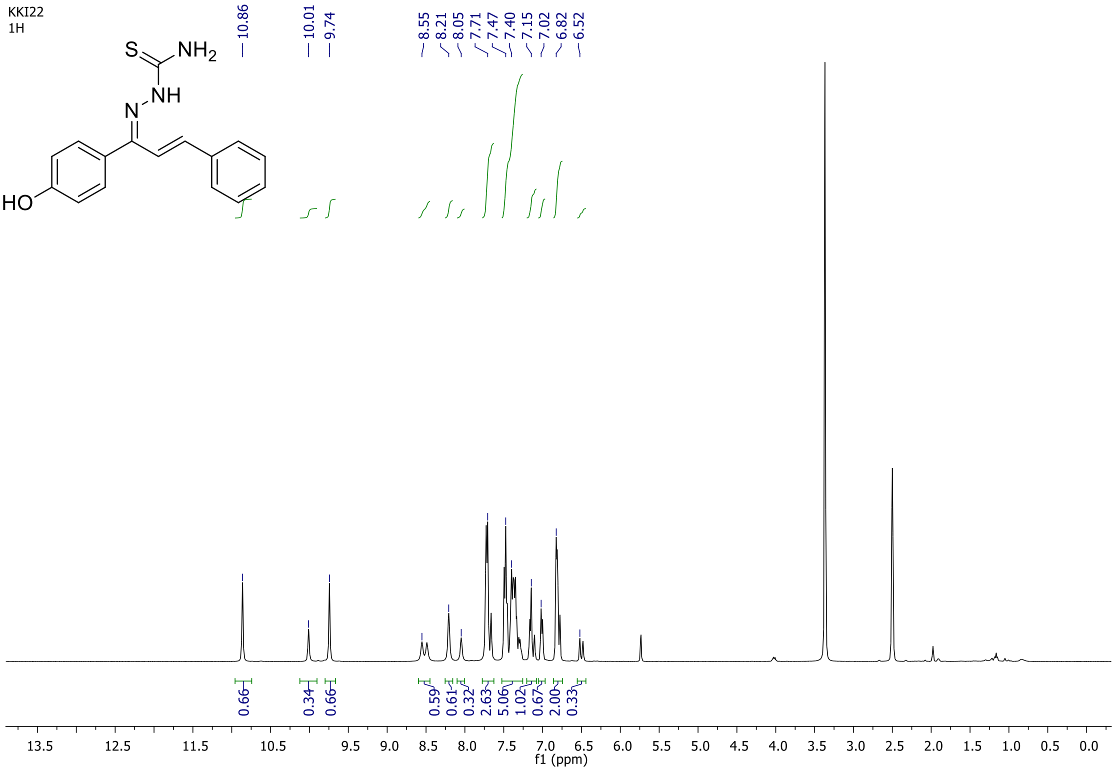

Figure SI_13. ^{1}H NMR of compound **25** ((*E*)-2-((*E*)-1-(4-hydroxyphenyl)-3-phenylallylidene)hydrazine-1-carbothioamide) in DMSO

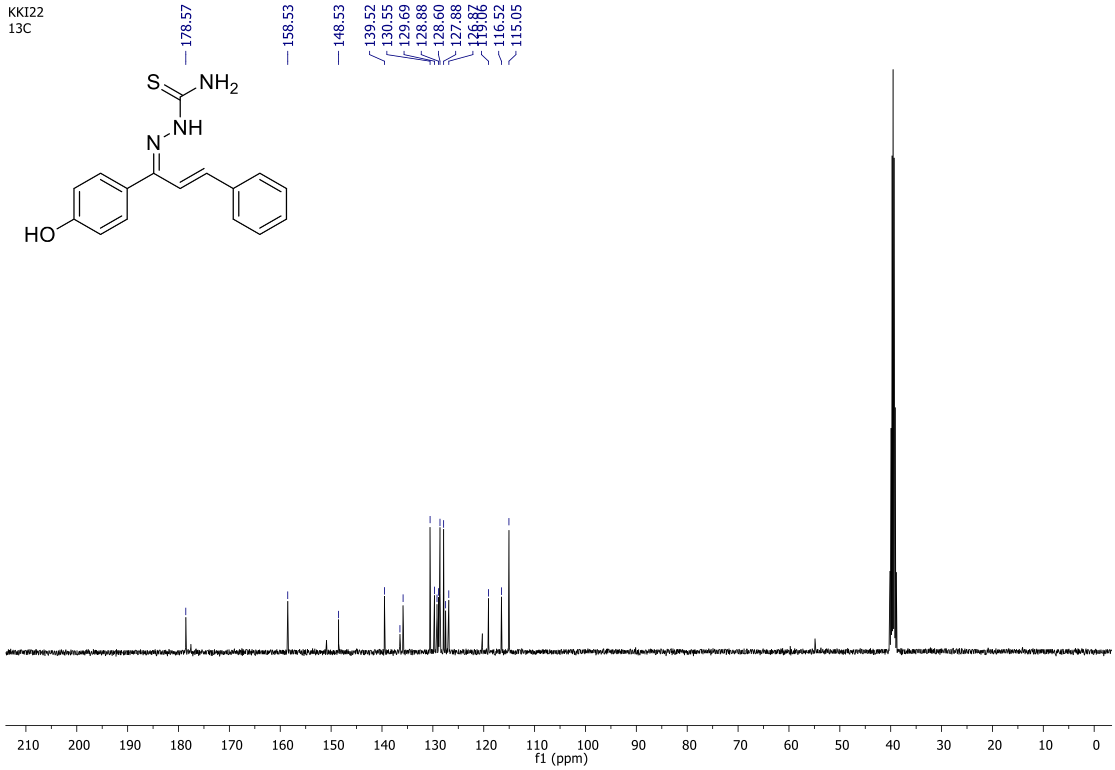

Figure SI_14. ^{13}C NMR of compound **25** ((*E*)-2-((*E*)-1-(4-hydroxyphenyl)-3-phenylallylidene)hydrazine-1-carbothioamide) in DMSO

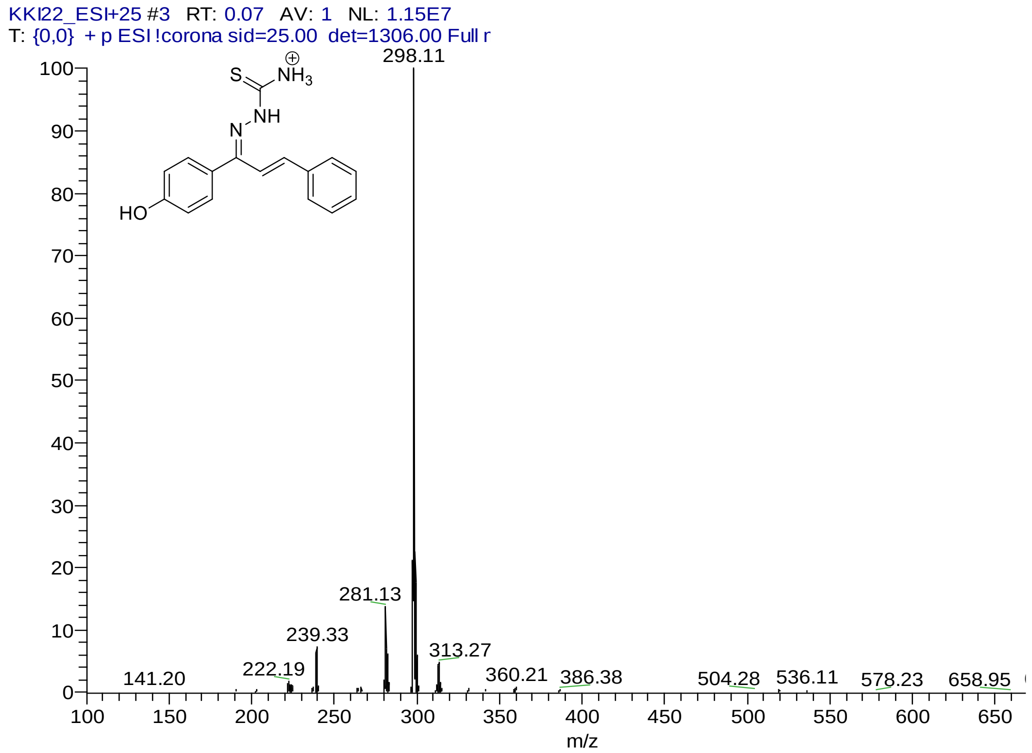

Figure SI_15. ESI MS of compound **25** ((*E*)-2-((*E*)-1-(4-hydroxyphenyl)-3-phenylallylidene)hydrazine-1-carbothioamide)

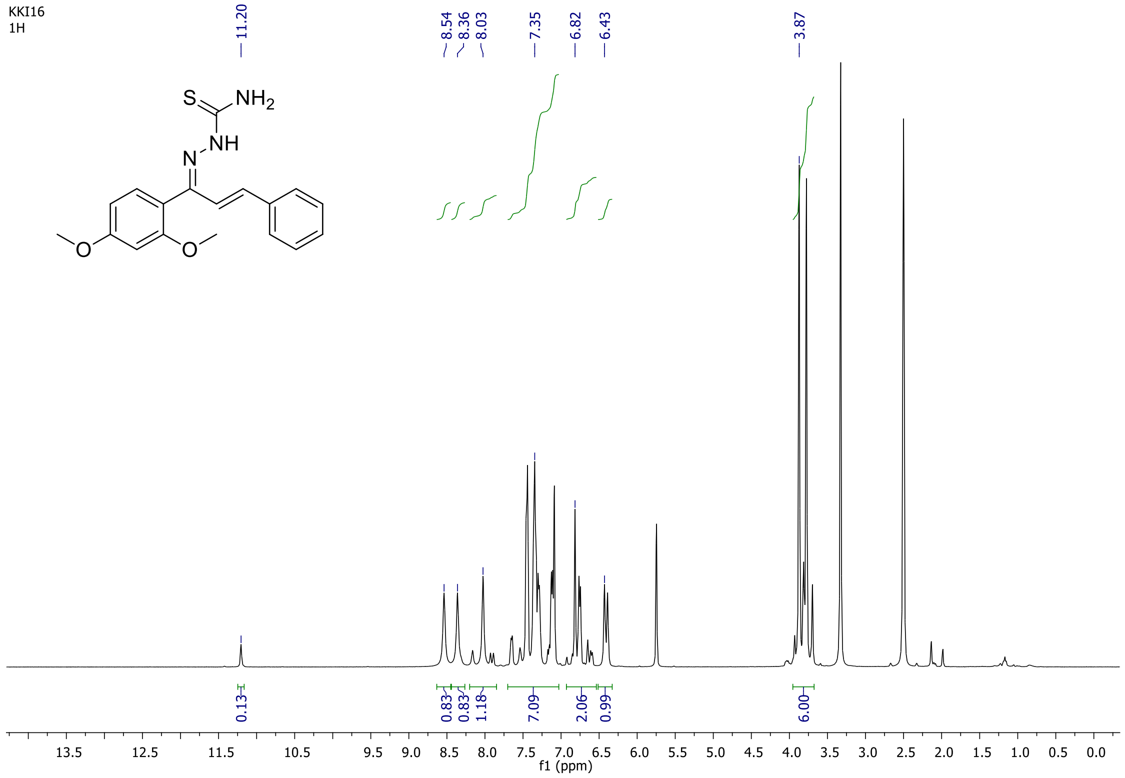

Figure SI_16. ^{1}H NMR of compound **26** ((*E*)-2-((*E*)-1-(2,4-dimethoxyphenyl)-3-phenylallylidene)hydrazine-1-carbothioamide) in DMSO

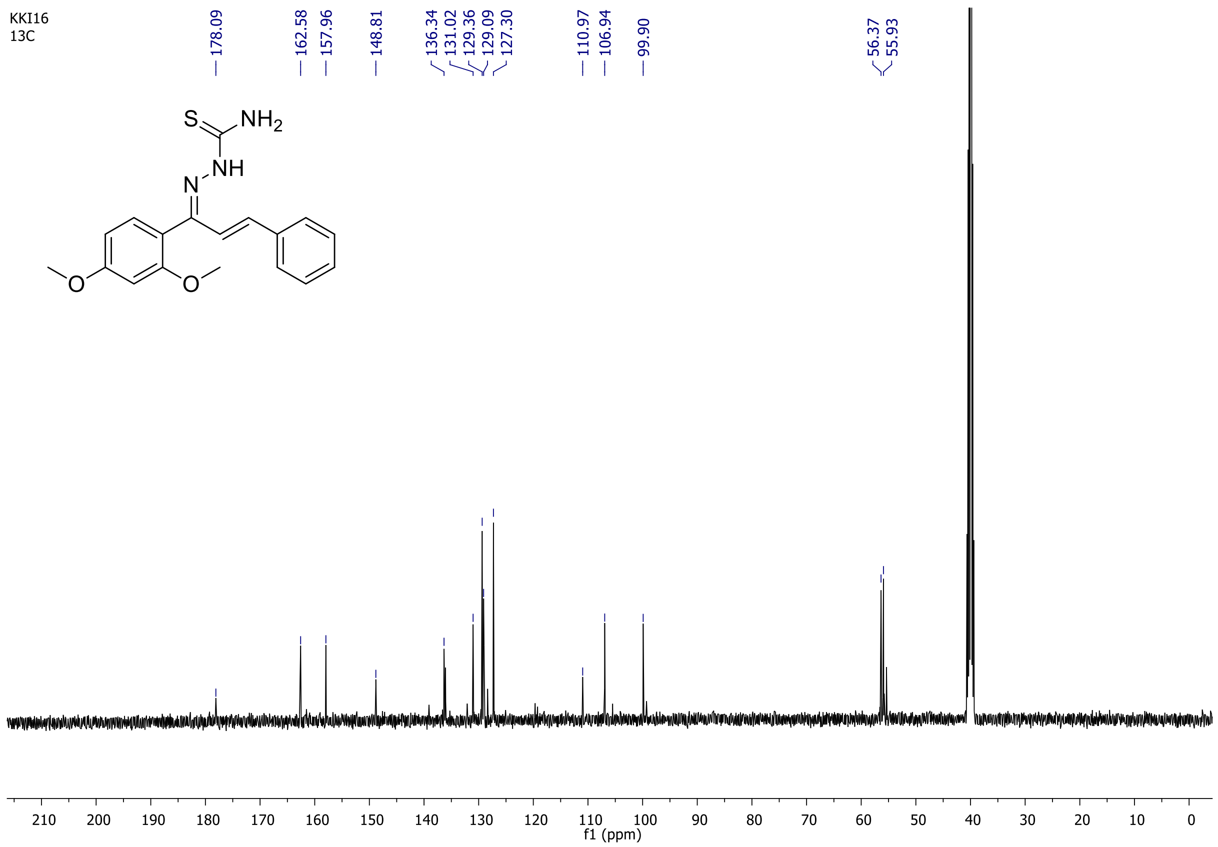

Figure SI_17. ^{13}C NMR of compound **26** ((*E*)-2-((*E*)-1-(2,4-dimethoxyphenyl)-3-phenylallylidene)hydrazine-1-carbothioamide) in DMSO

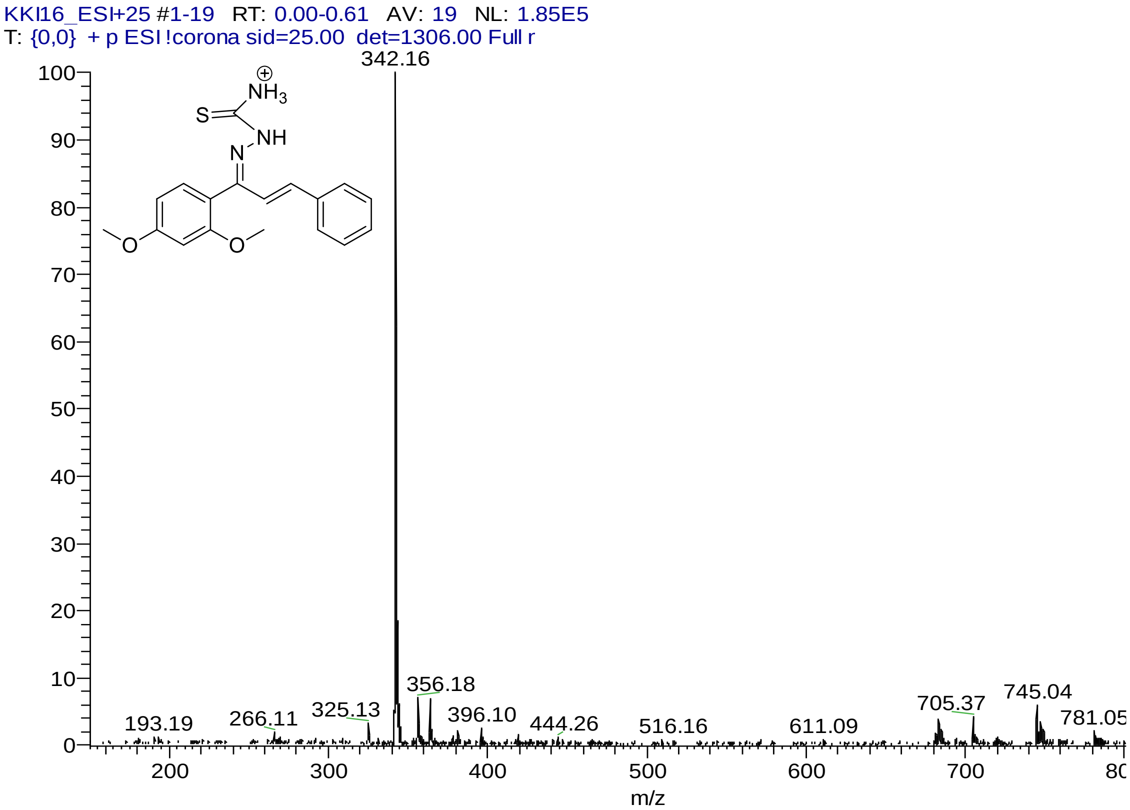

Figure SI_18. ESI MS of compound **26** ((*E*)-2-((*E*)-1-(2,4-dimethoxyphenyl)-3-phenylallylidene)hydrazine-1-carbothioamide)

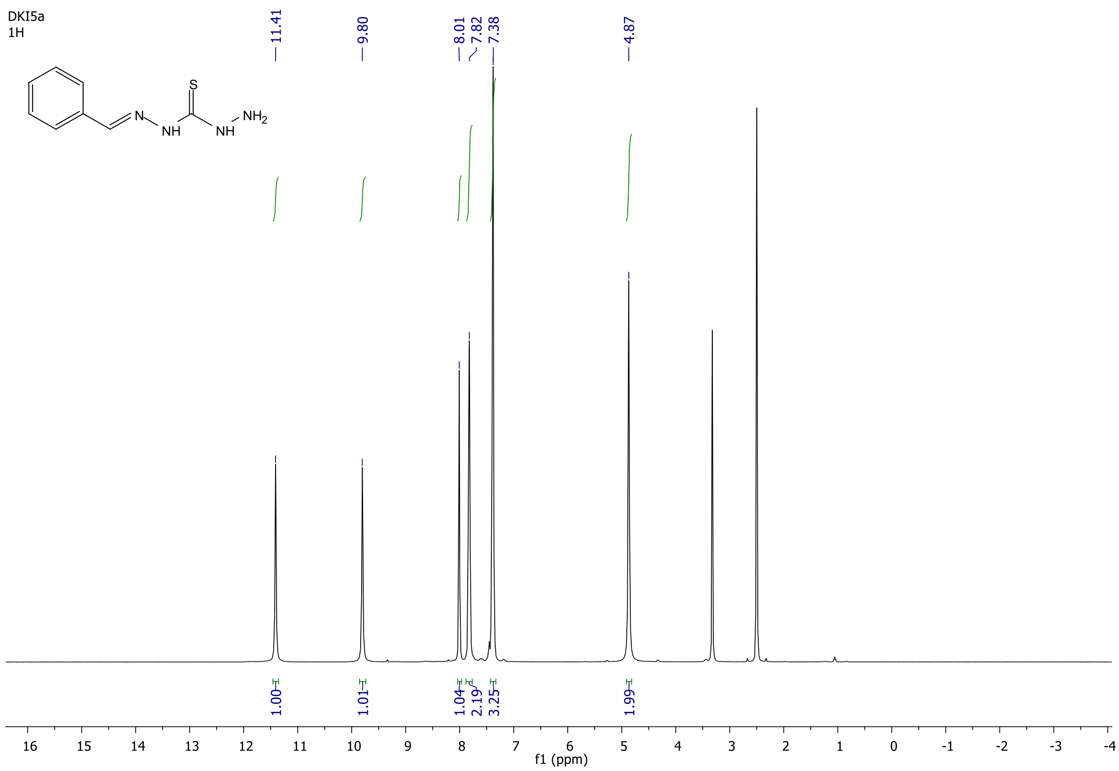

Figure SI_19. ^{1}H NMR of compound **27** ((*E*)-*N'*-benzylidenehydrazinecarbothiohydrazide) in DMSO

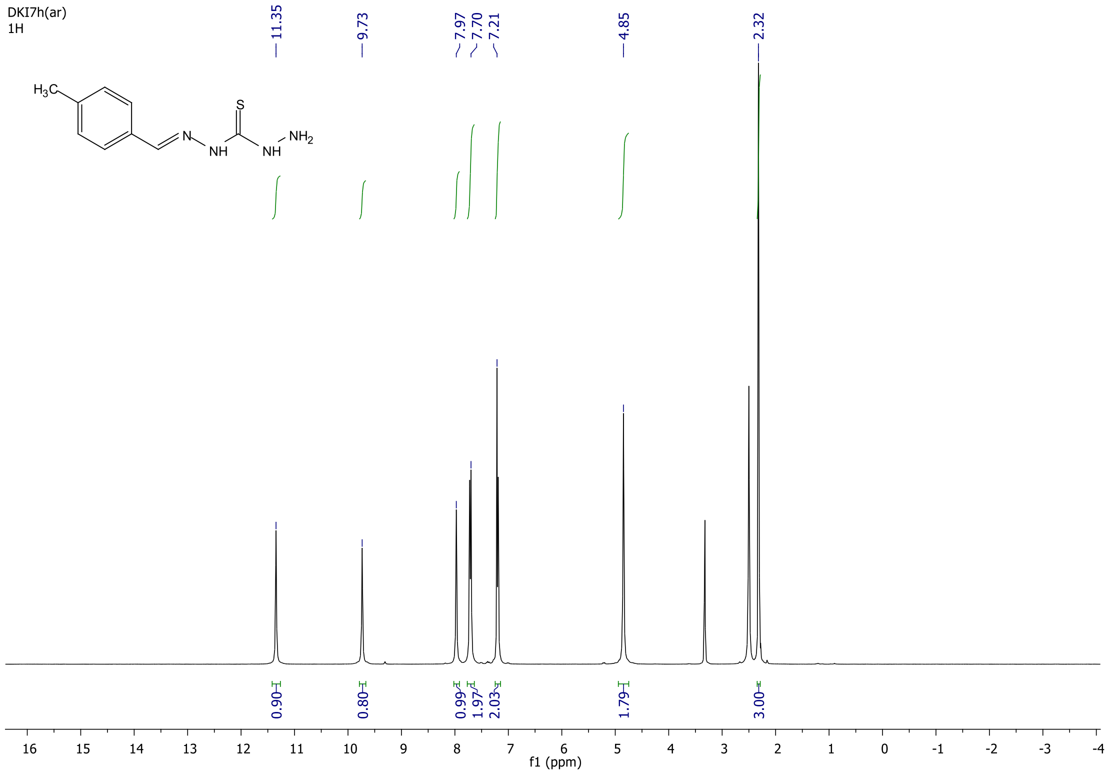

Figure SI_20. ^{1}H NMR of compound **28** ((*E*)-*N'*-(4-methylbenzylidene)hydrazinecarbothiohydrazide) in DMSO

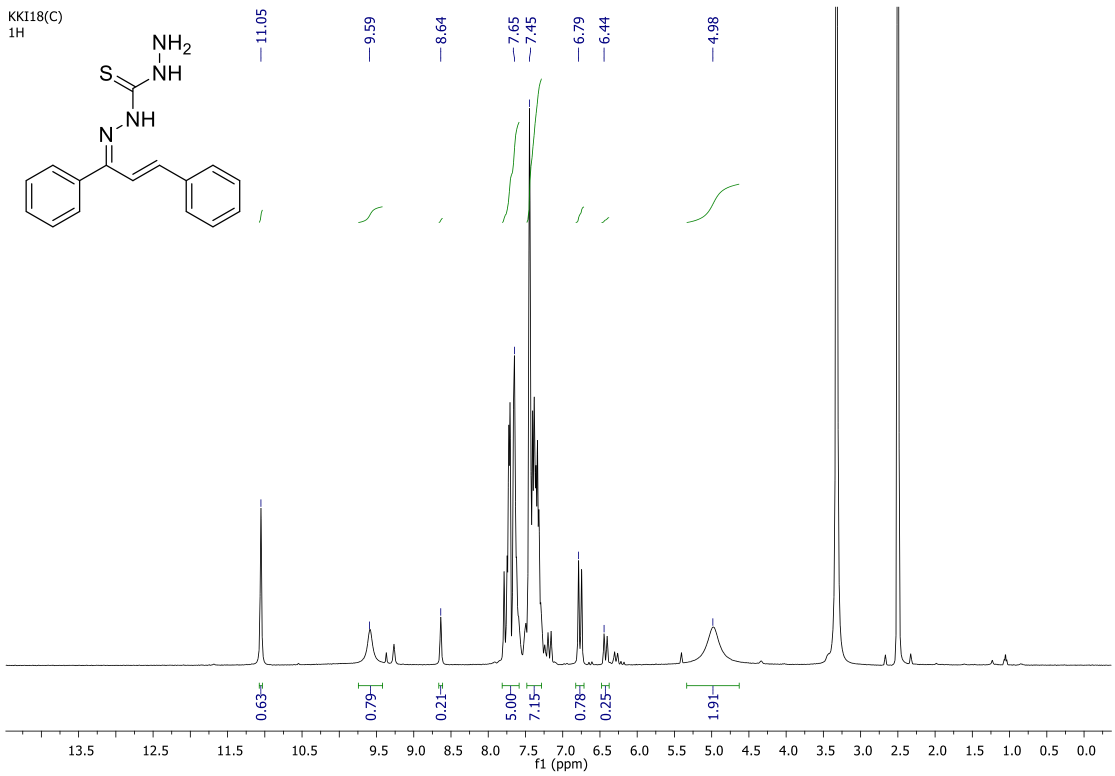

Figure SI_21. ^{1}H NMR of compound **29** (*N'*-((1*E*,2*E*)-1,3-diphenylallylidene)hydrazinecarbothiohydrazide) in DMSO

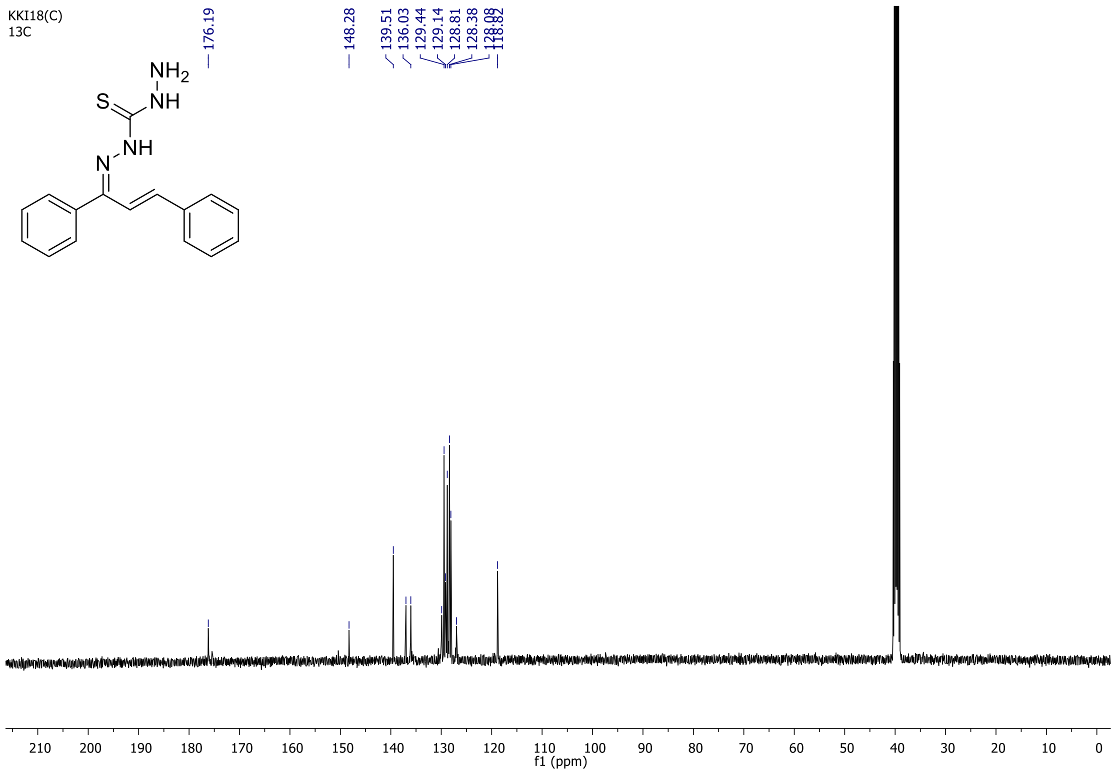

Figure SI_22. ^{13}C NMR of compound **29** (*N'*-((1*E*,2*E*)-1,3-diphenylallylidene)hydrazinecarbothiohydrazide) in DMSO

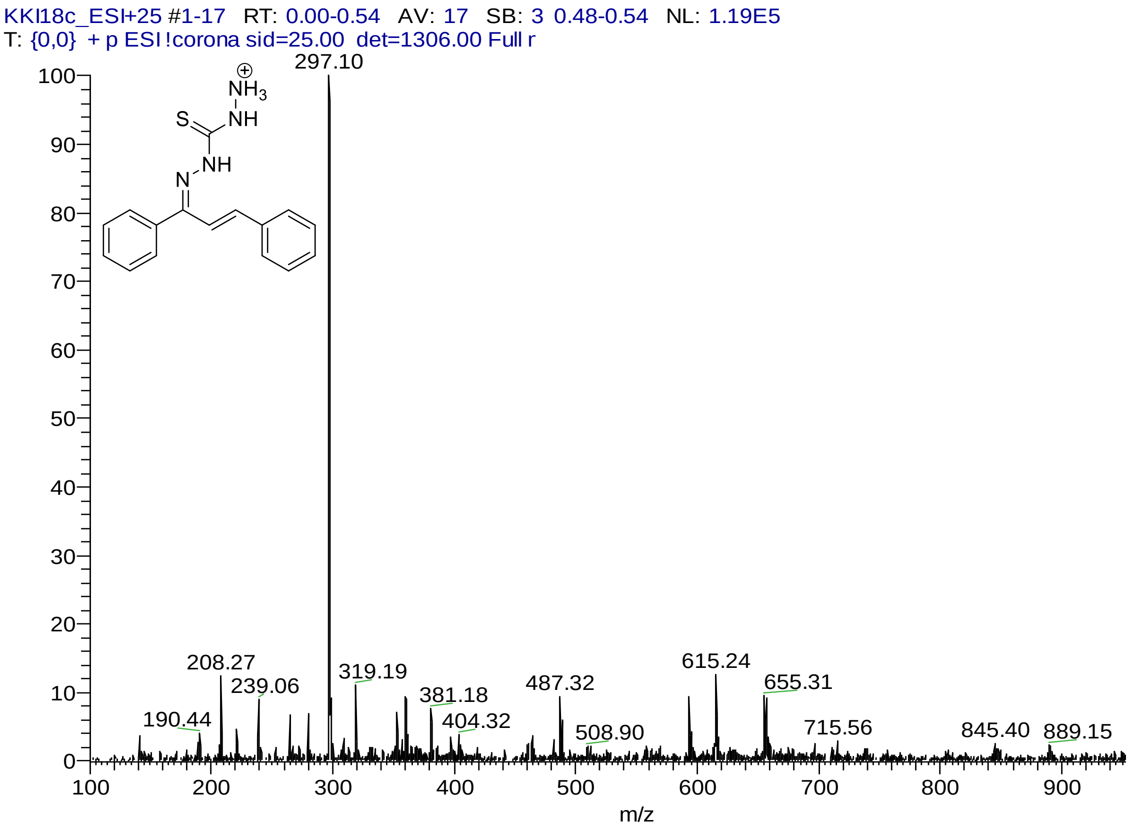

Figure SI_23. ESI MS of compound **29** (*N'*-((1*E*,2*E*)-1,3-diphenylallylidene)hydrazinecarbothiohydrazide)

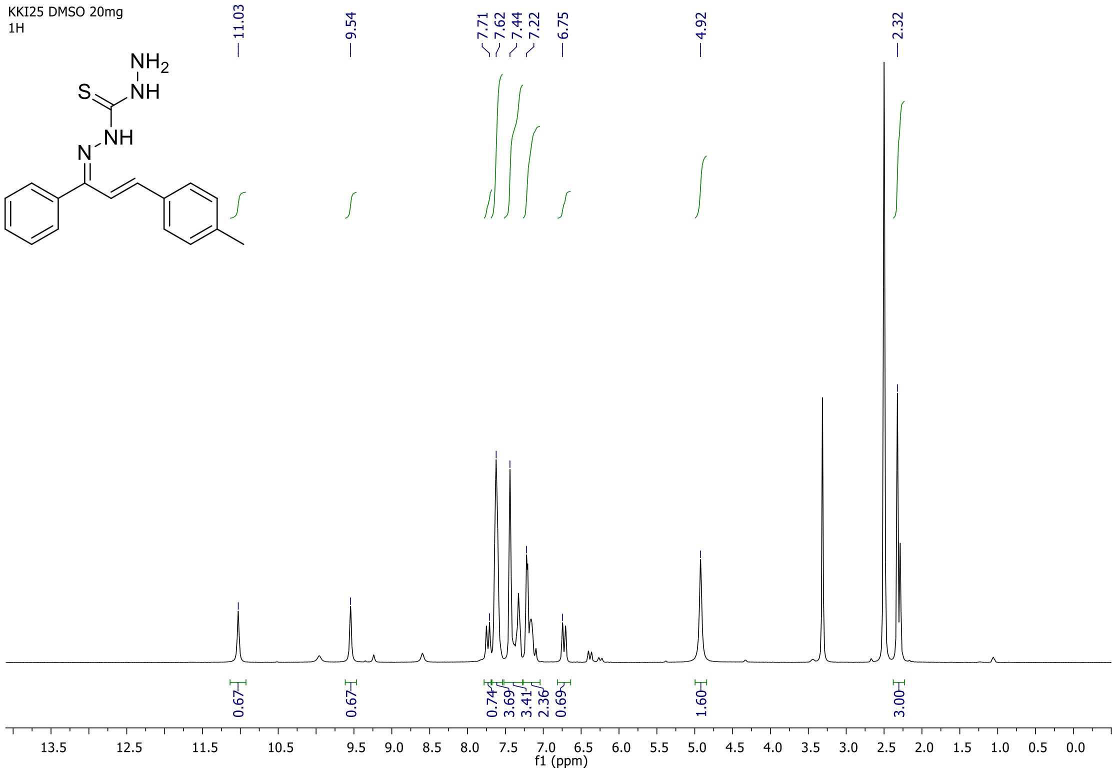

Figure SI_24. ^{1}H NMR of compound **30** (*N'*-((1*E*,2*E*)-1-phenyl-3-(p-tolyl)allylidene)hydrazinecarbothiohydrazide) in DMSO

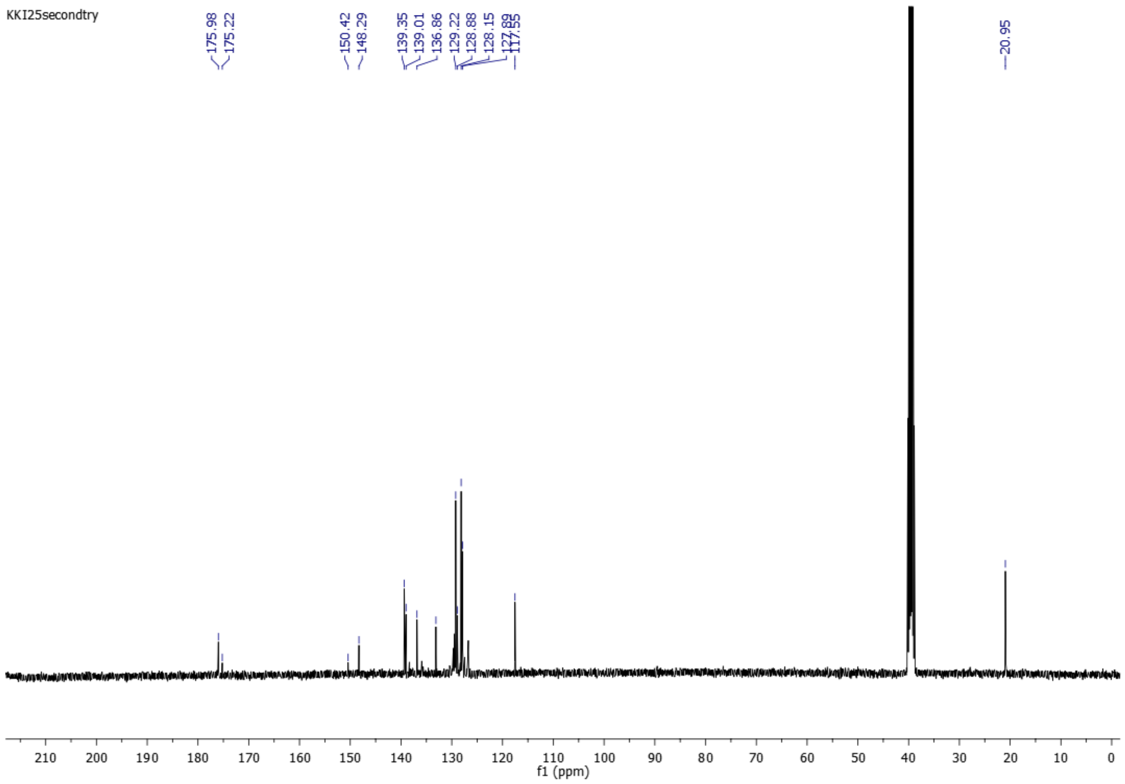

Figure SI_25. ^{13}C NMR of compound **30** (*N'*-((1*E*,2*E*)-1-phenyl-3-(p-tolyl)allylidene)hydrazinecarbothiohydrazide) in DMSO

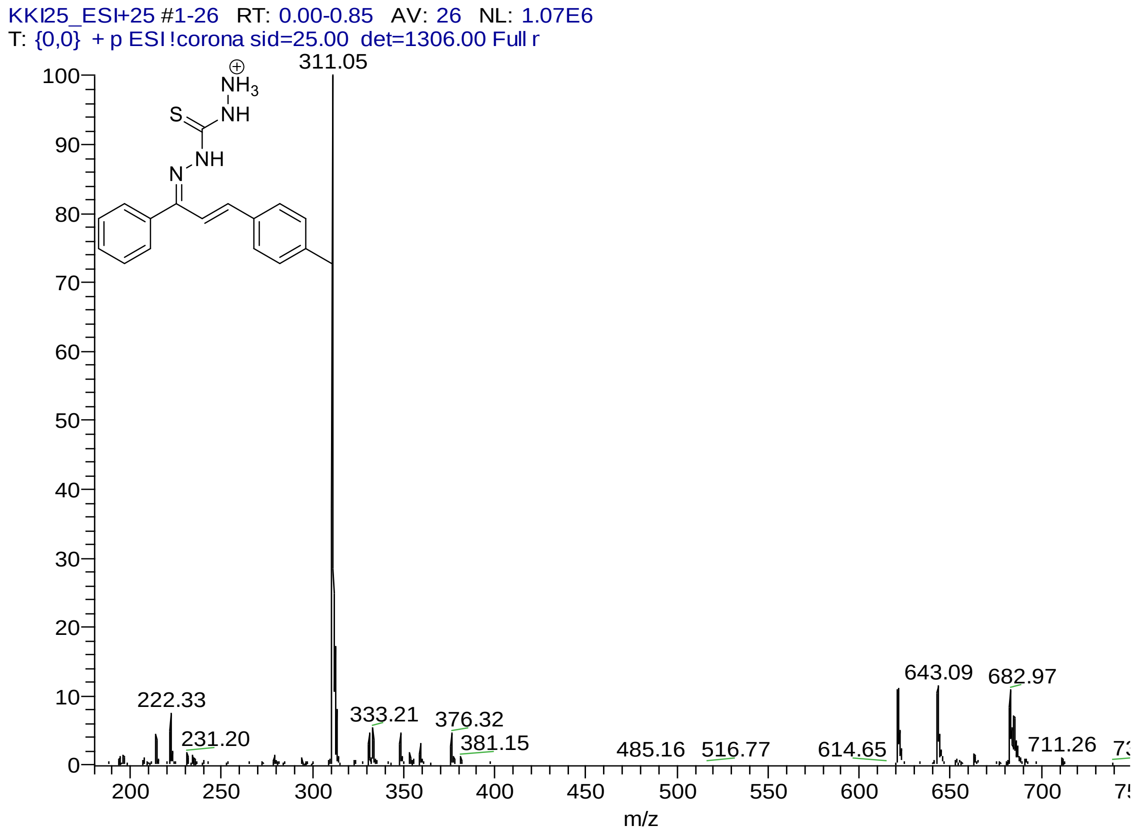

Figure SI_26. ESI MS of compound **30** (*N'*-((1*E*,2*E*)-1-phenyl-3-(p-tolyl)allylidene)hydrazinecarbothiohydrazide)

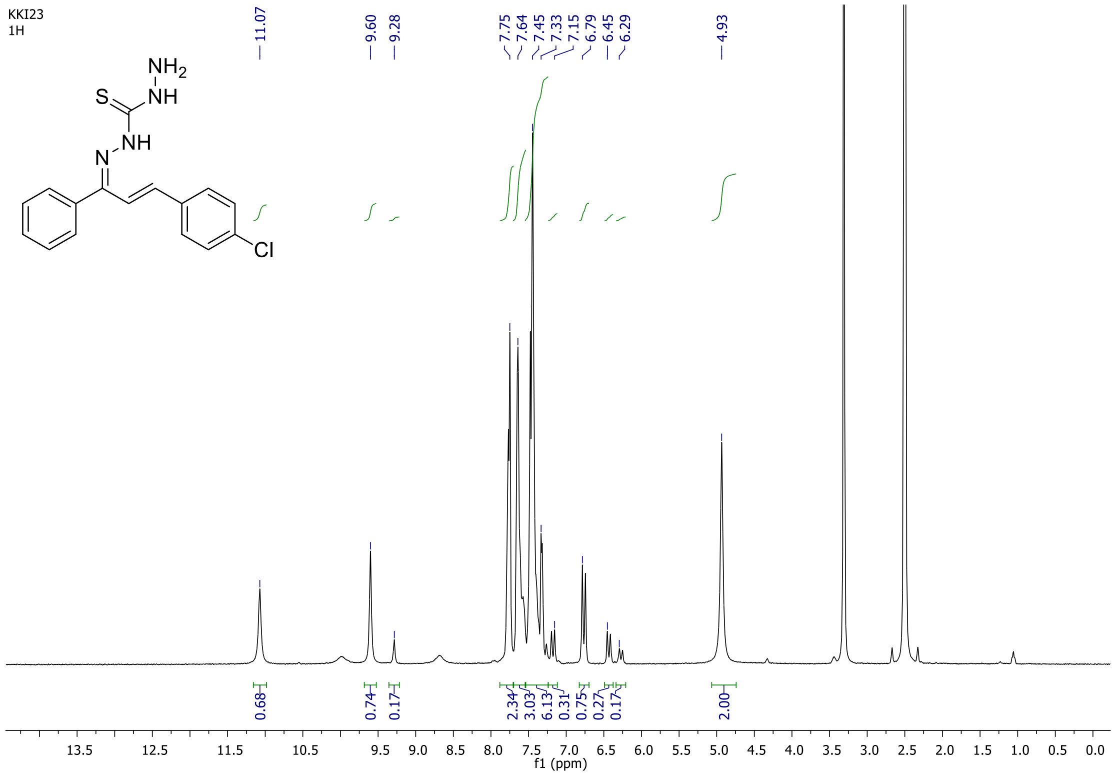

Figure SI_27. ^{1}H NMR of compound **31** (*N'*-((1*E*,2*E*)-3-(4-chlorophenyl)-1-phenylallylidene)hydrazinecarbothiohydrazide) in DMSO

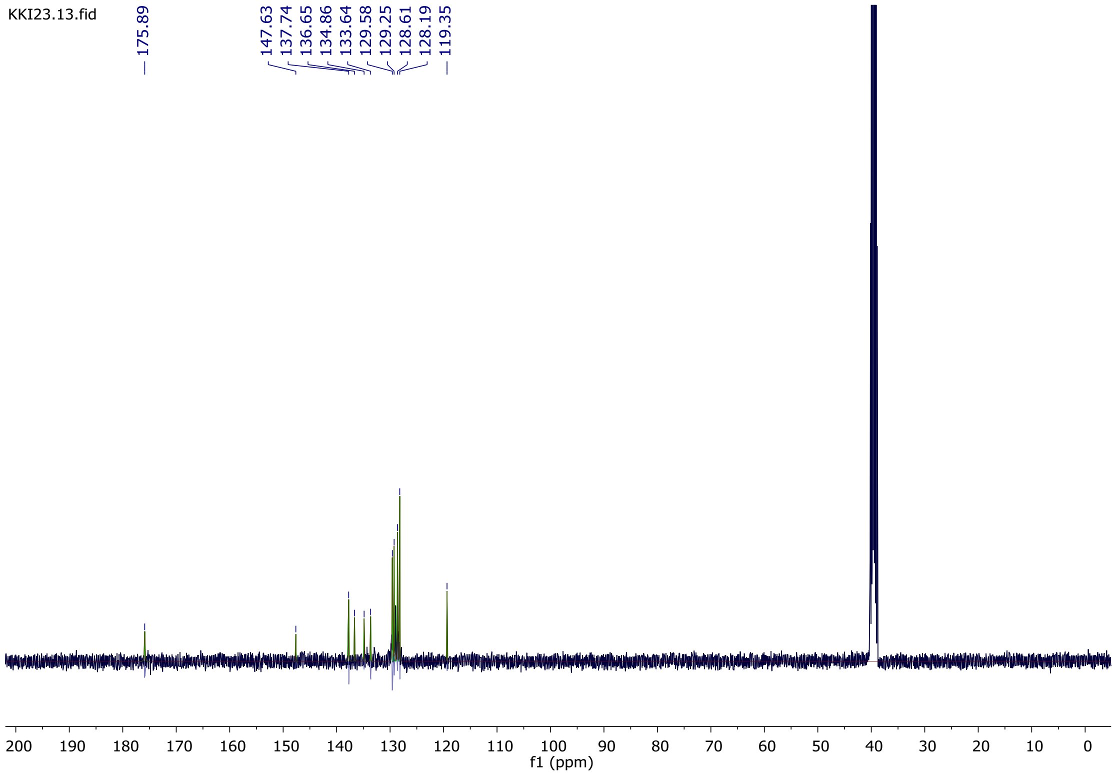

Figure SI_28. ^{13}C NMR of compound **31** (*N'*-((1*E*,2*E*)-3-(4-chlorophenyl)-1-phenylallylidene)hydrazinecarbothiohydrazide) in DMSO

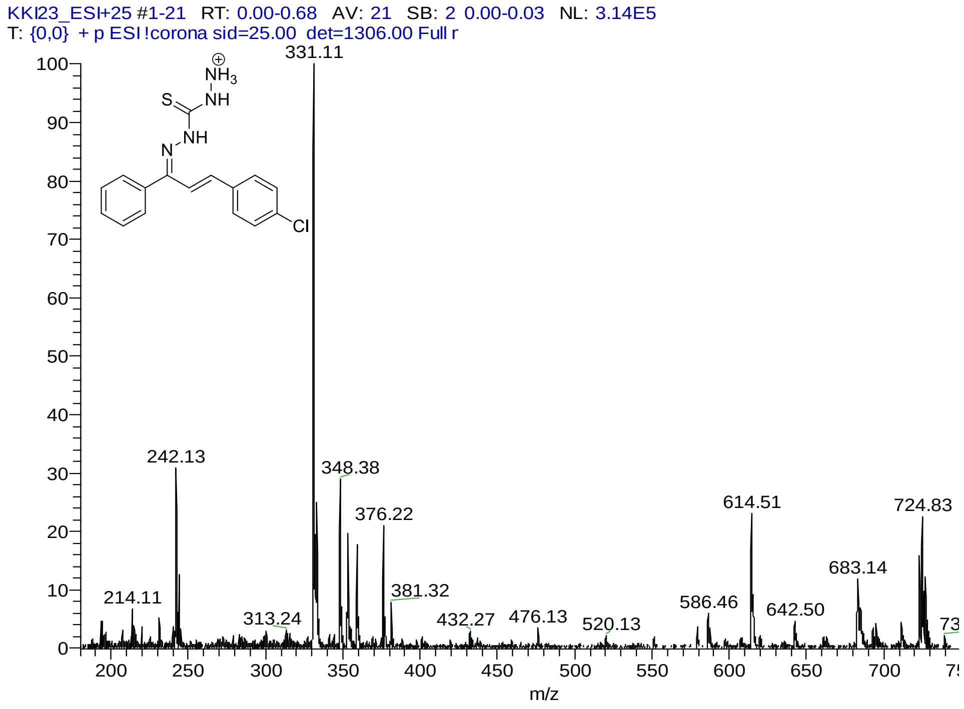

Figure SI_29. ESI MS of compound **31** (*N'*-((1*E*,2*E*)-3-(4-chlorophenyl)-1-phenylallylidene)hydrazinecarbothiohydrazide)

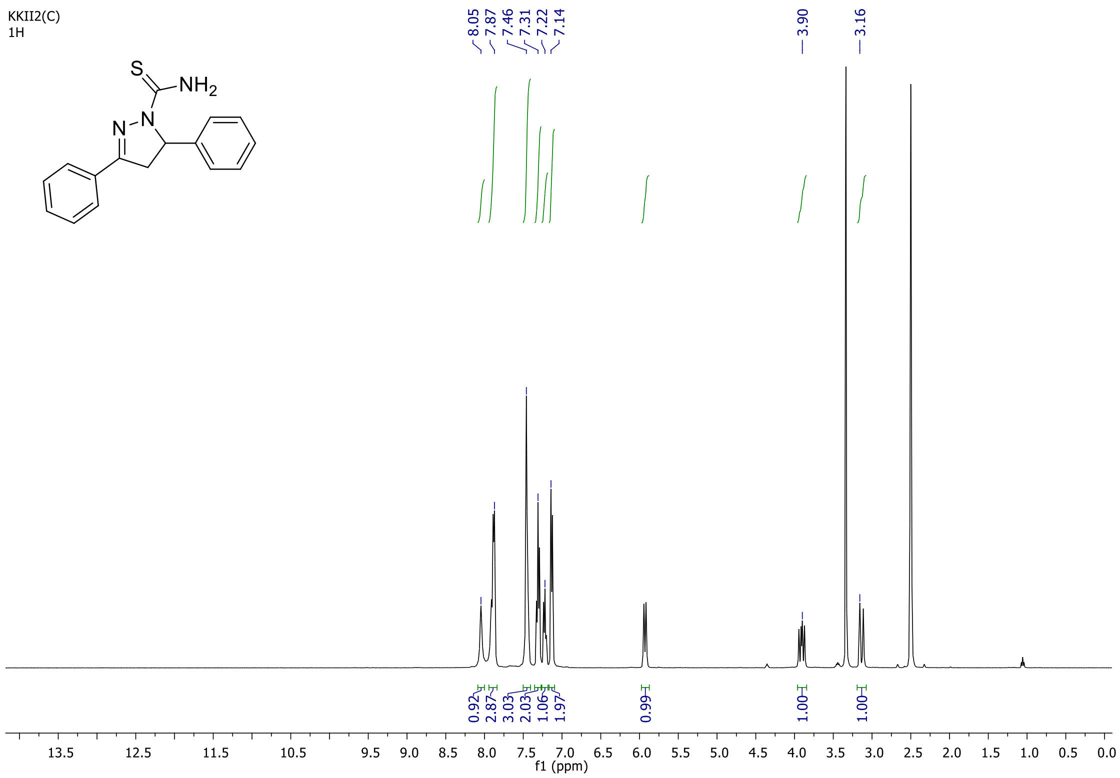

Figure SI_30. ^{1}H NMR of compound **32** (3,5-diphenyl-4,5-dihydro-1H-pyrazole-1-carbothioamide) in DMSO

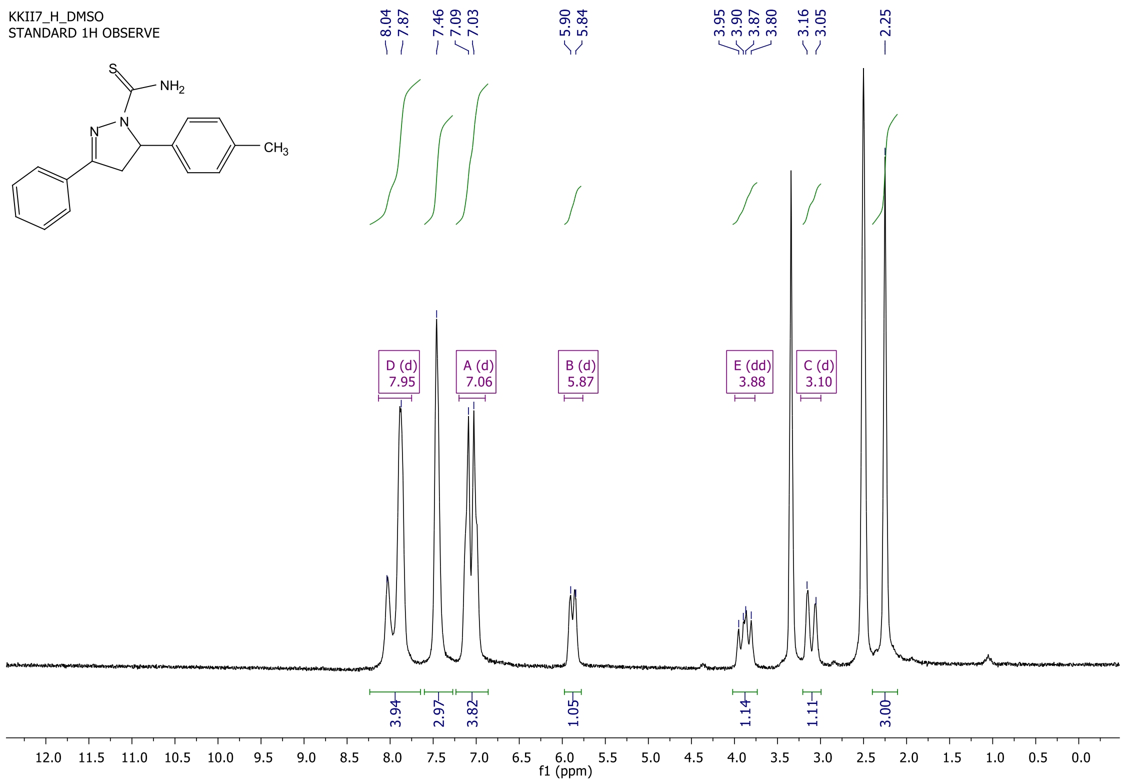

Figure SI_31. ^{1}H NMR of compound **33** (3-phenyl-5-(p-tolyl)-4,5-dihydro-1H-pyrazole-1-carbothioamide) in DMSO

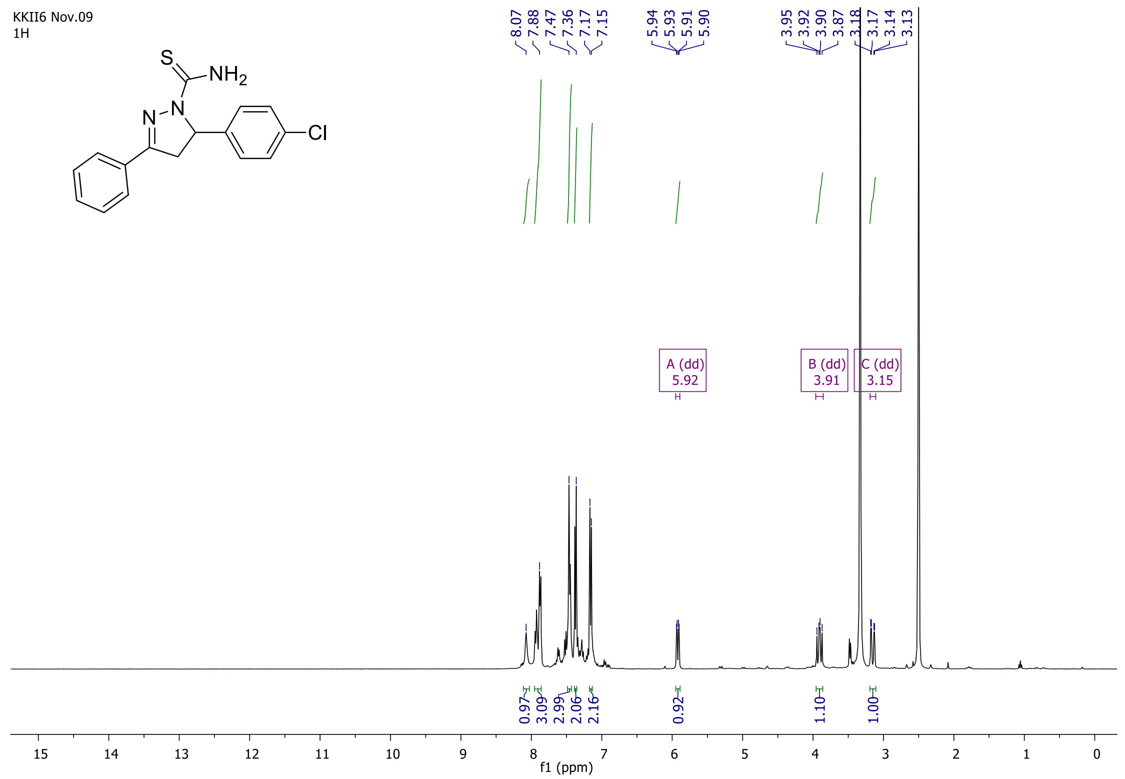

Figure SI_32. ^{1}H NMR of compound **34** (5-(4-chlorophenyl)-3-phenyl-4,5-dihydro-1H-pyrazole-1-carbothioamide) in DMSO

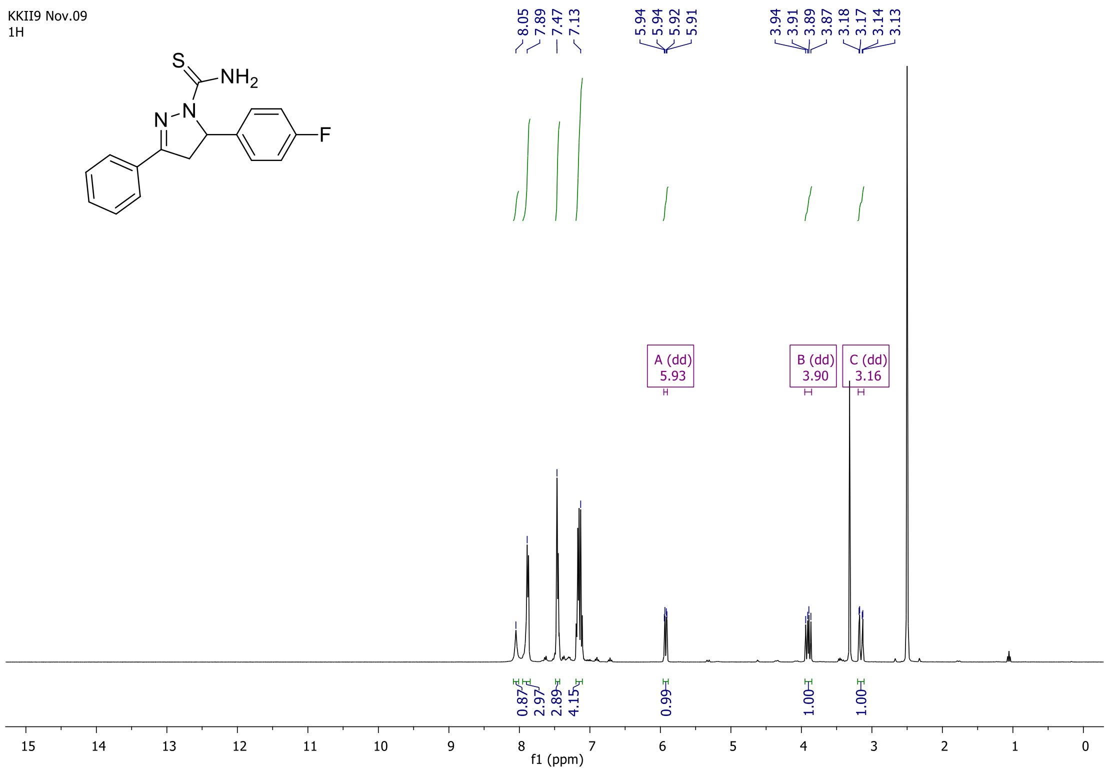

Figure SI_33. ^{1}H NMR of compound **35** (5-(4-fluorophenyl)-3-phenyl-4,5-dihydro-1H-pyrazole-1-carbothioamide) in DMSO

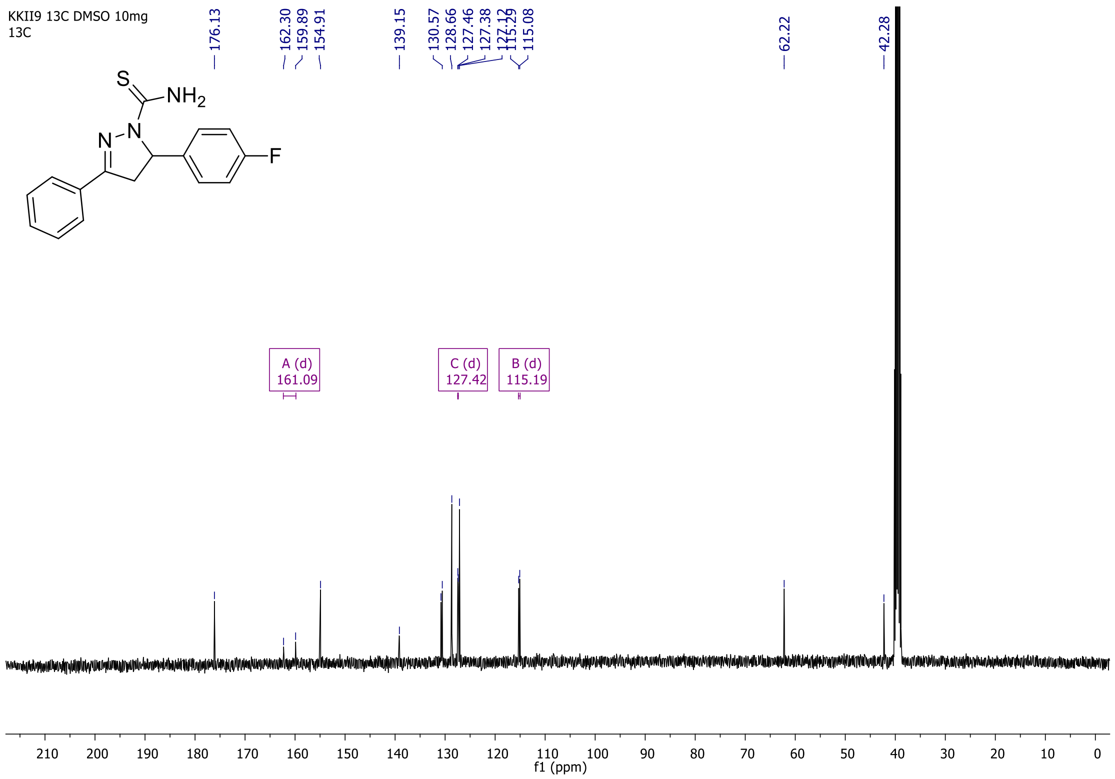

Figure SI_34. ^{13}C NMR of compound **35** (5-(4-fluorophenyl)-3-phenyl-4,5-dihydro-1H-pyrazole-1-carbothioamide) in DMSO

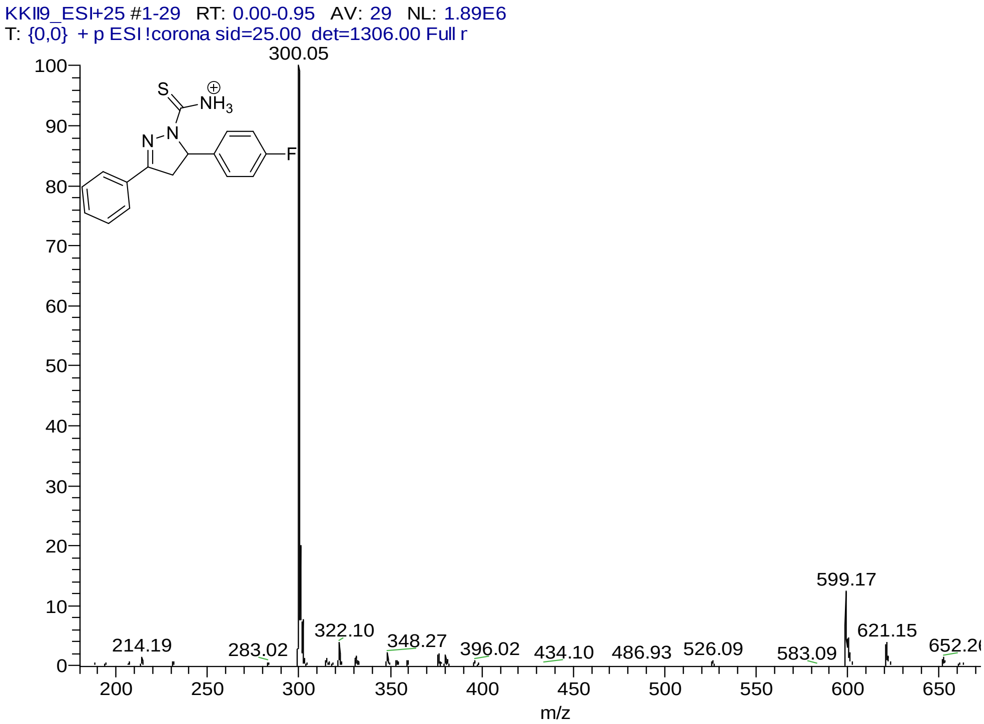

Figure SI_35. ESI MS of compound **35** (5-(4-fluorophenyl)-3-phenyl-4,5-dihydro-1H-pyrazole-1-carbothioamide)

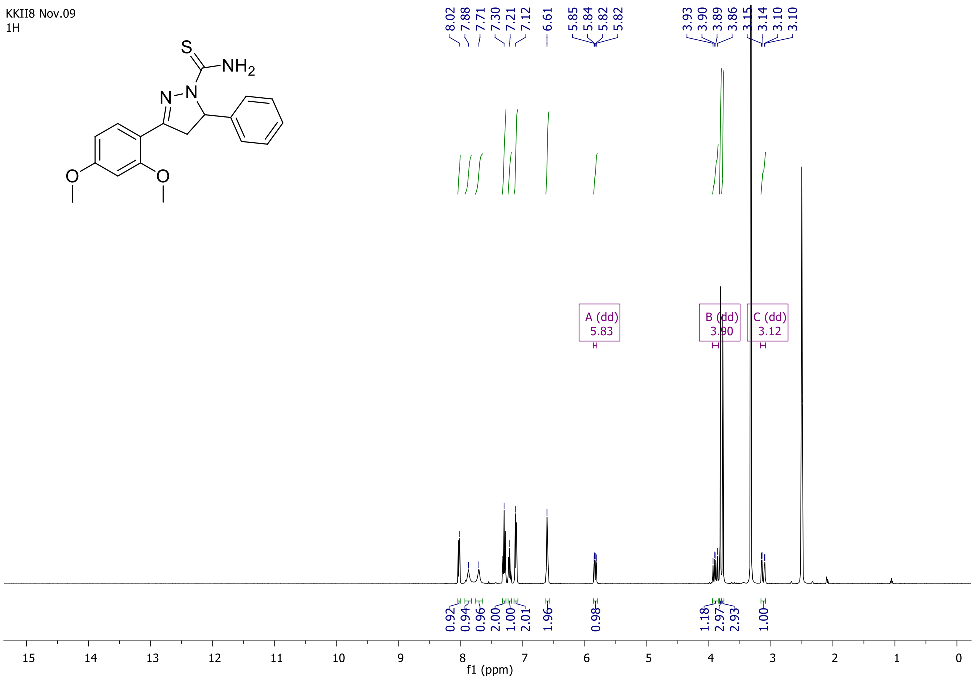

Figure SI_36. 1H NMR of compound **36** (3-(2,4-dimethoxyphenyl)-5-phenyl-4,5-dihydro-1H-pyrazole-1-carbothioamide) in DMSO

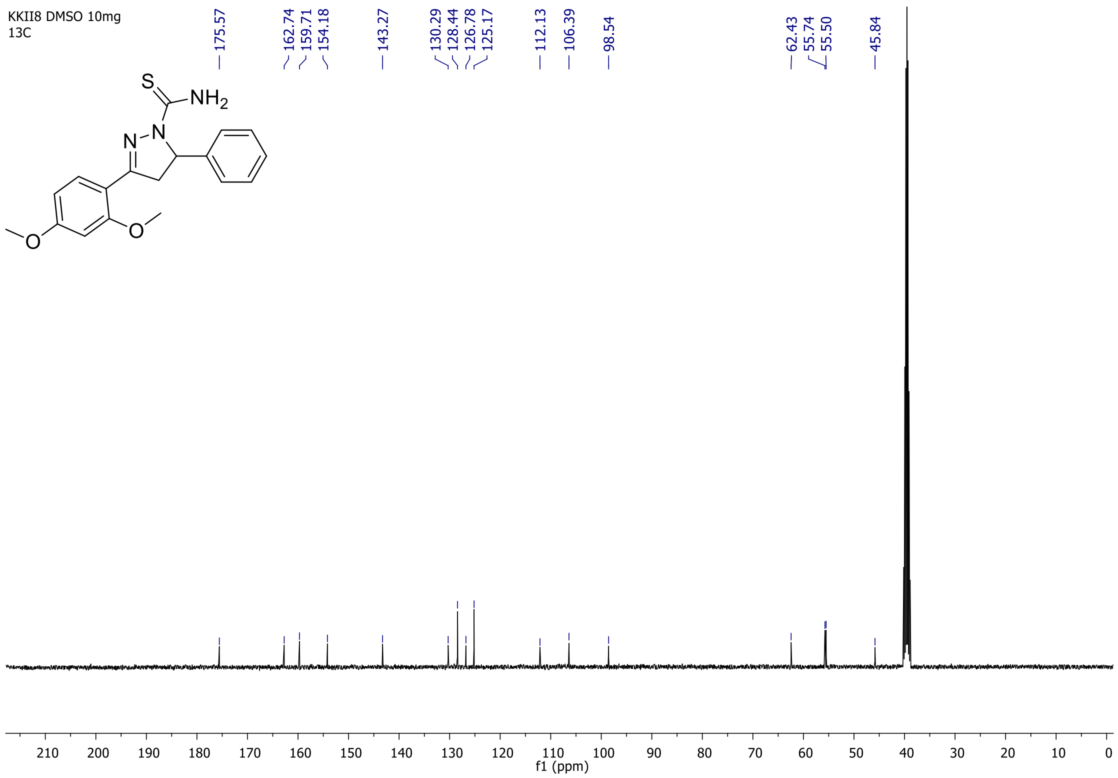

Figure SI_37. ^{13}C NMR of compound **36** (3-(2,4-dimethoxyphenyl)-5-phenyl-4,5-dihydro-1H-pyrazole-1-carbothioamide) in DMSO

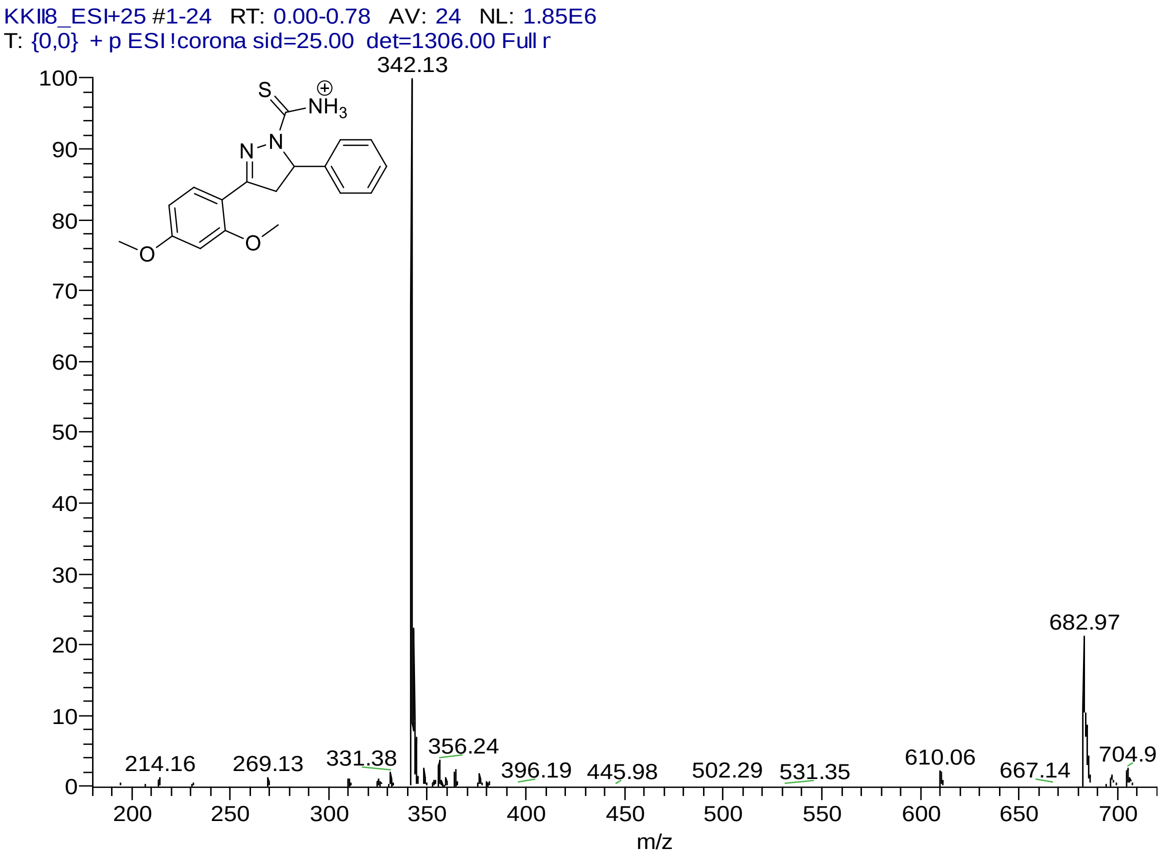

Figure SI_38. ESI MS of compound **36** (3-(2,4-dimethoxyphenyl)-5-phenyl-4,5-dihydro-1H-pyrazole-1-carbothioamide)

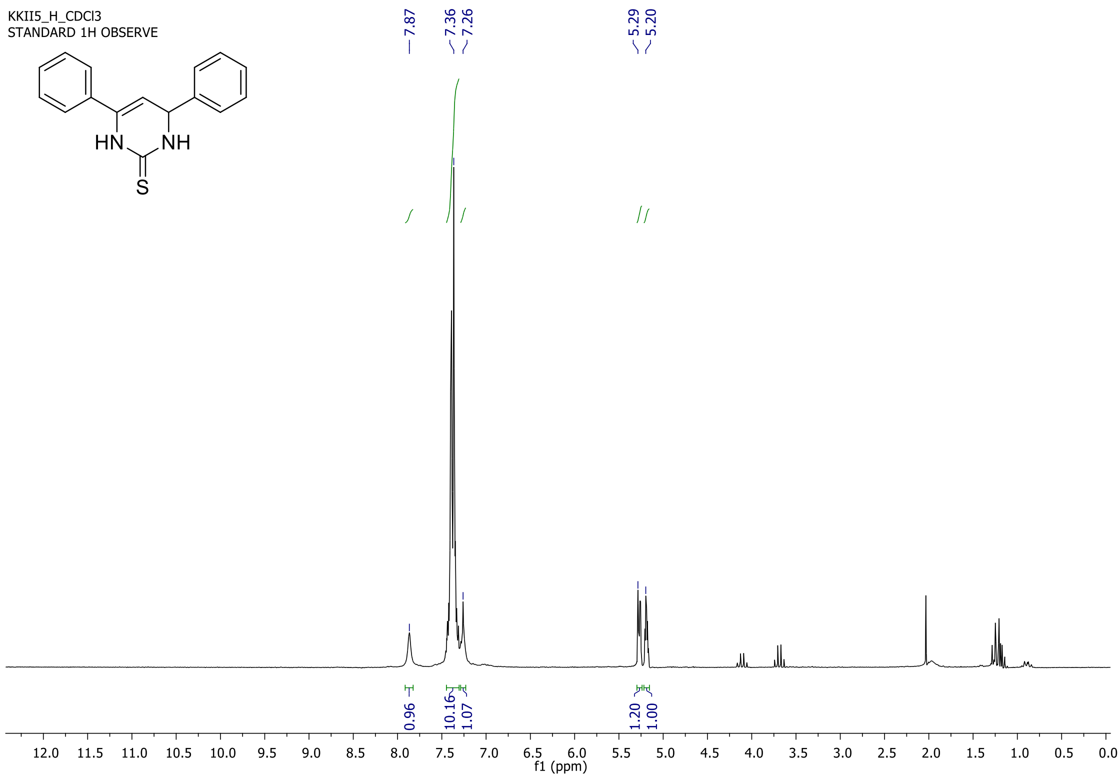

Figure SI_39. ^{1}H NMR of compound **37** (4,6-diphenyl-3,4-dihydropyrimidine-2(1H)-thione**)** in CDCl3

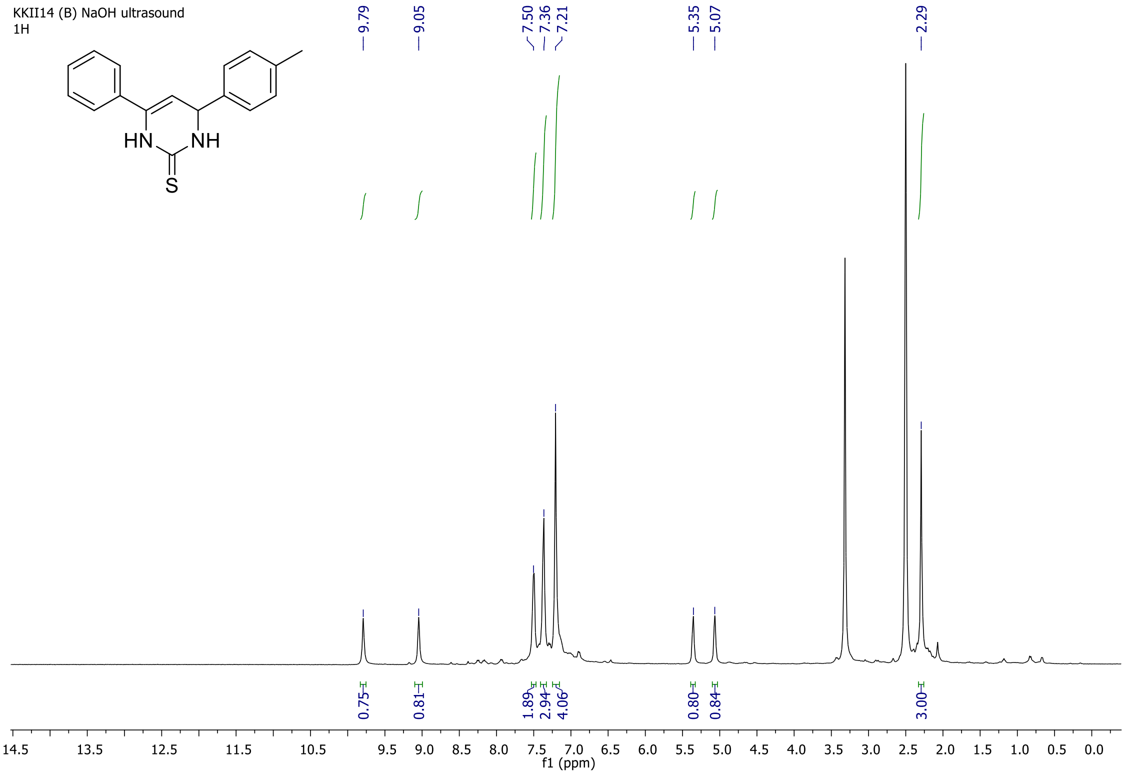

Figure SI_40. ^{1}H NMR of compound **38** (6-phenyl-4-(p-tolyl)-3,4-dihydropyrimidine-2(1H)-thione**)** in DMSO

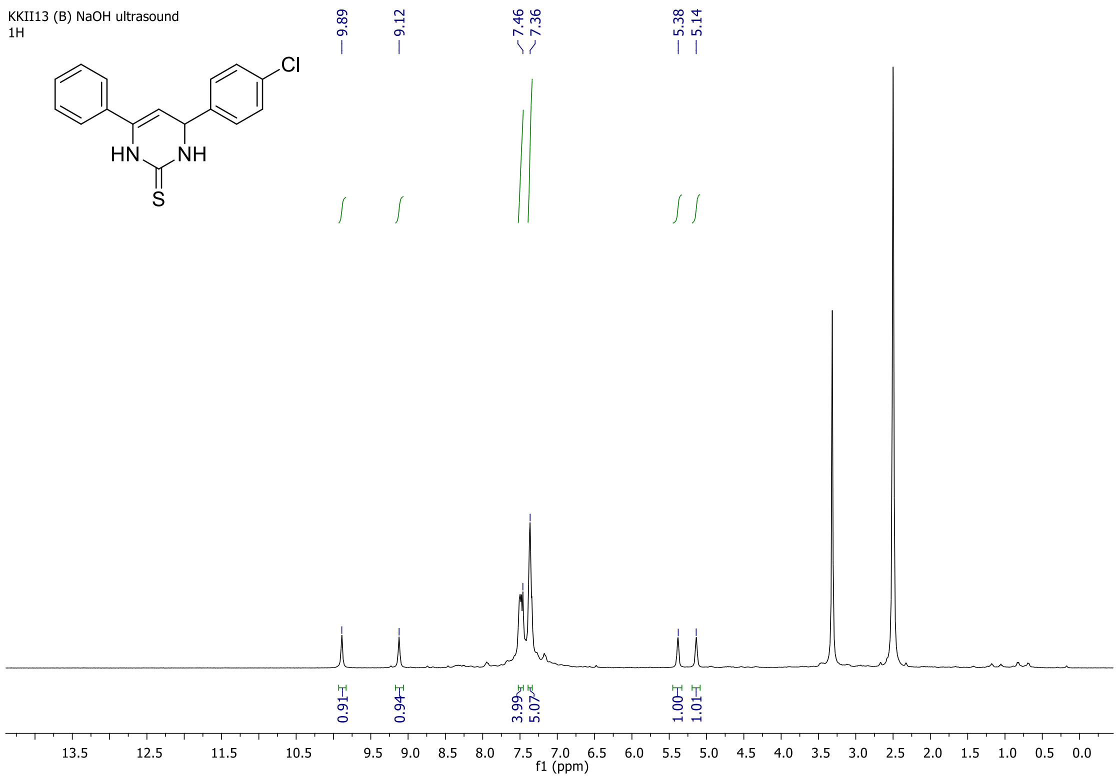

Figure SI_41. ^{1}H NMR of compound **39** (4-(4-chlorophenyl)-6-phenyl-3,4-dihydropyrimidine-2(1H)-thione) in DMSO

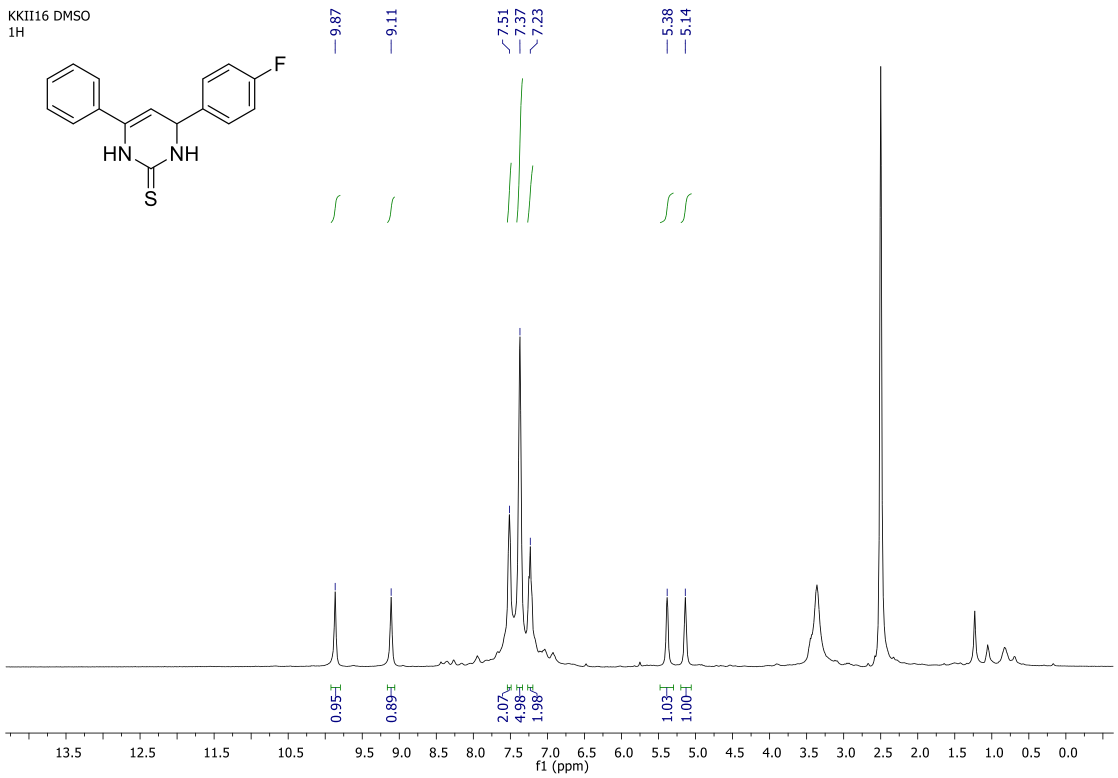

Figure SI_42. ^{1}H NMR of compound **40** (4-(4-fluorophenyl)-6-phenyl-3,4-dihydropyrimidine-2(1H)-thione) in DMSO

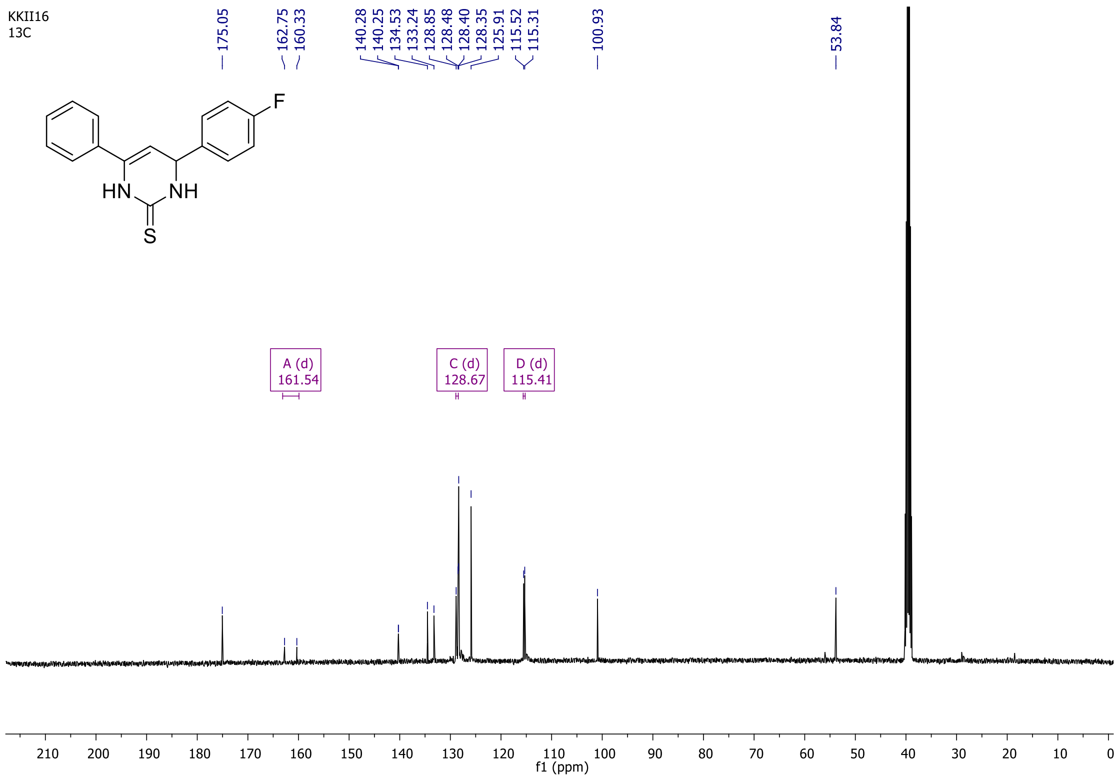

Figure SI_43. ^{13}C NMR of compound **40** (4-(4-fluorophenyl)-6-phenyl-3,4-dihydropyrimidine-2(1H)-thione) in DMSO

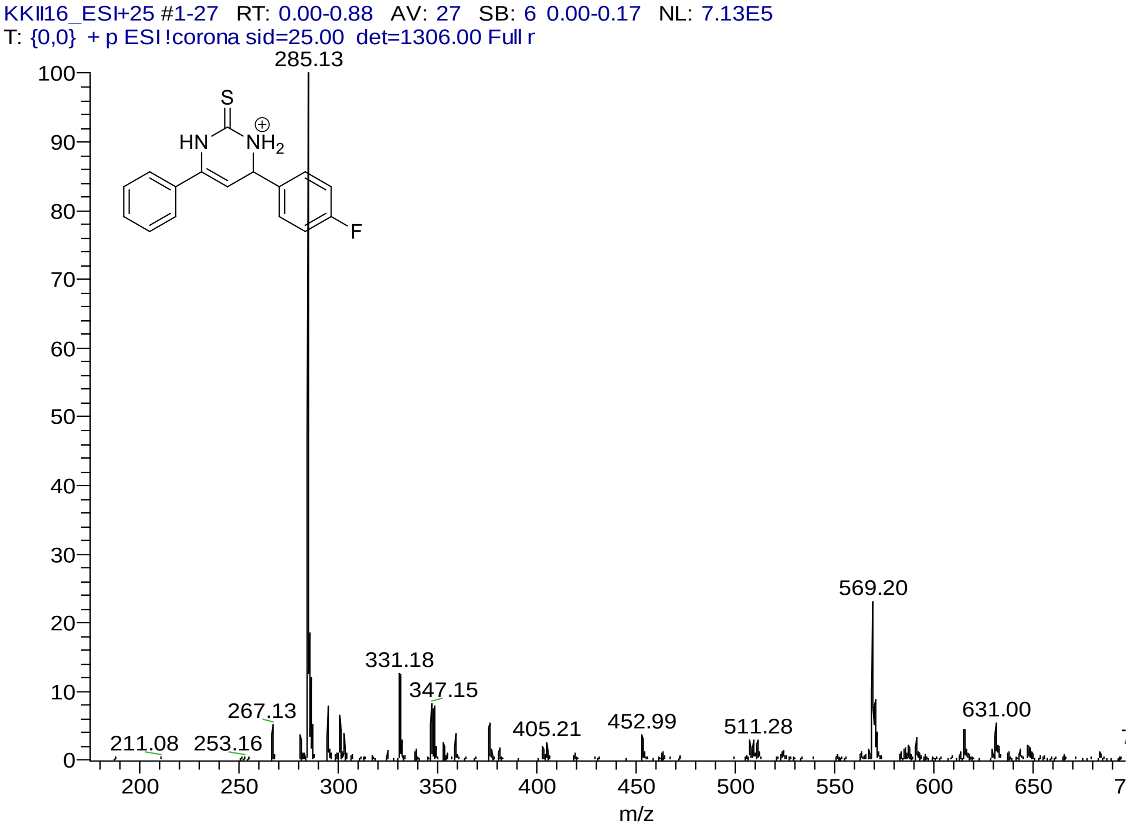

Figure SI_44. ESI MS of compound **40** (4-(4-fluorophenyl)-6-phenyl-3,4-dihydropyrimidine-2(1H)-thione)

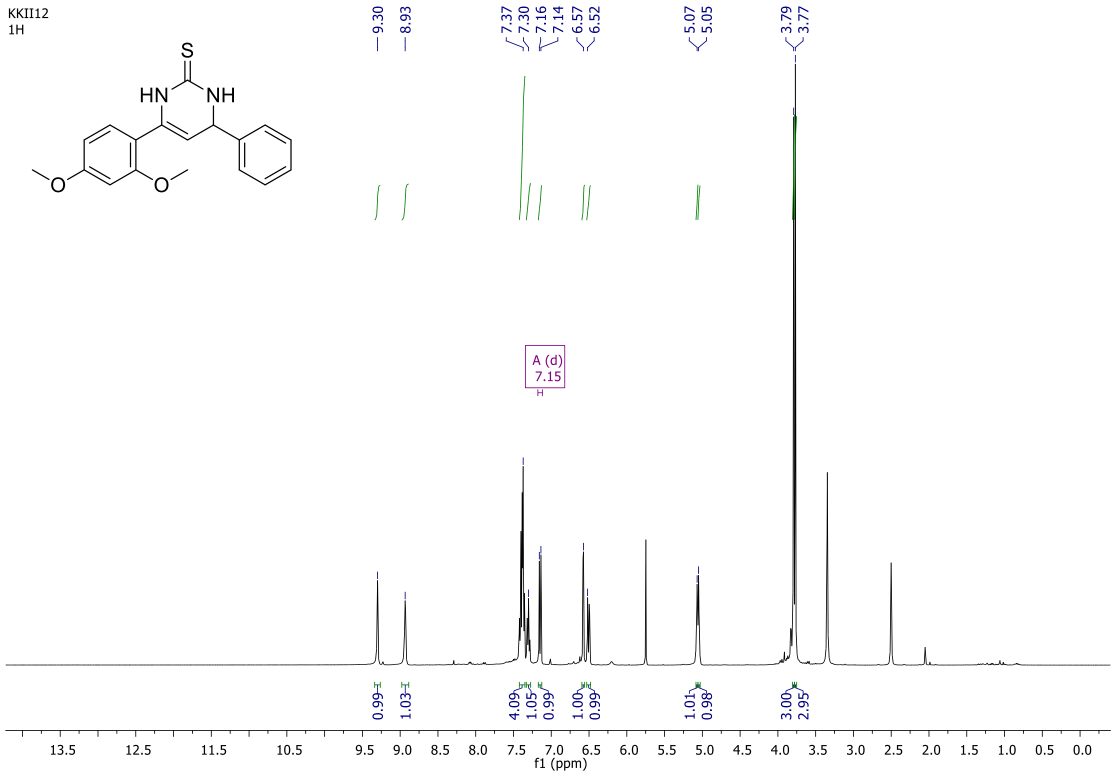

Figure SI_45. ^{1}H NMR of compound **41** (6-(2,4-dimethoxyphenyl)-4-phenyl-3,4-dihydropyrimidine-2(1H)-thione) in DMSO

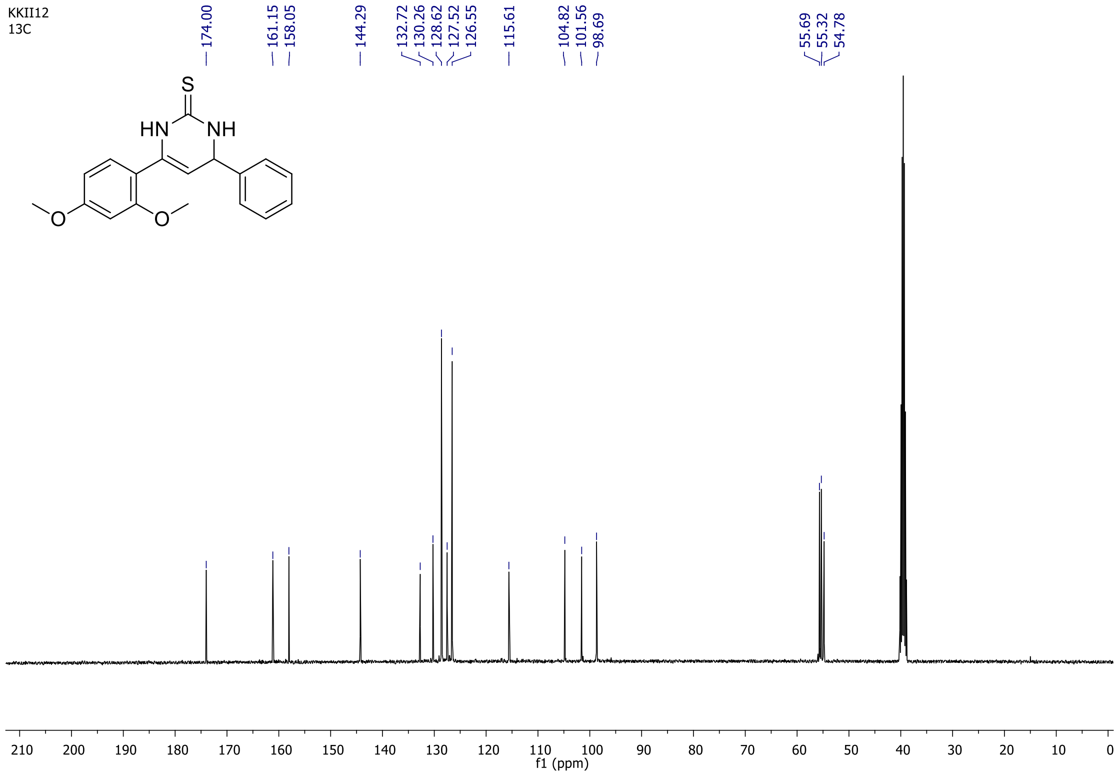

Figure SI_46. ^{13}C NMR of compound **41** (6-(2,4-dimethoxyphenyl)-4-phenyl-3,4-dihydropyrimidine-2(1H)-thione) in DMSO

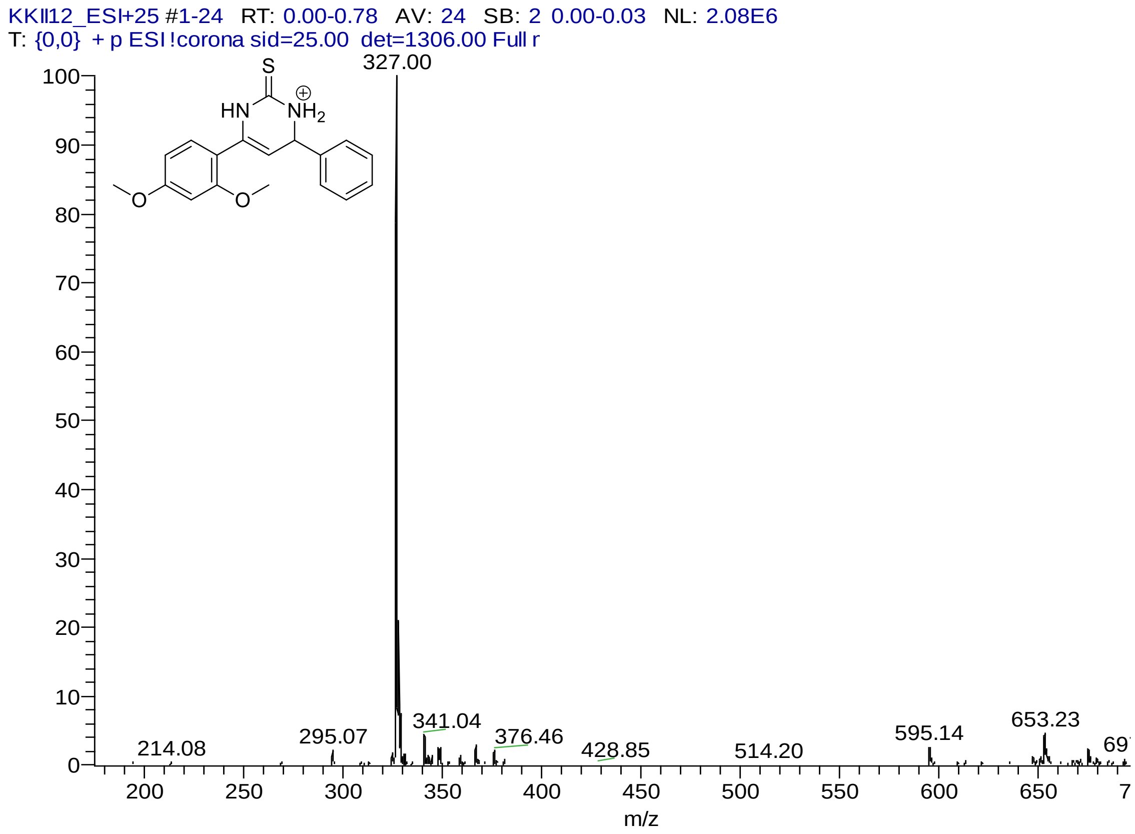

Figure SI_47. ESI MS of compound **41** (6-(2,4-dimethoxyphenyl)-4-phenyl-3,4-dihydropyrimidine-2(1H)-thione)

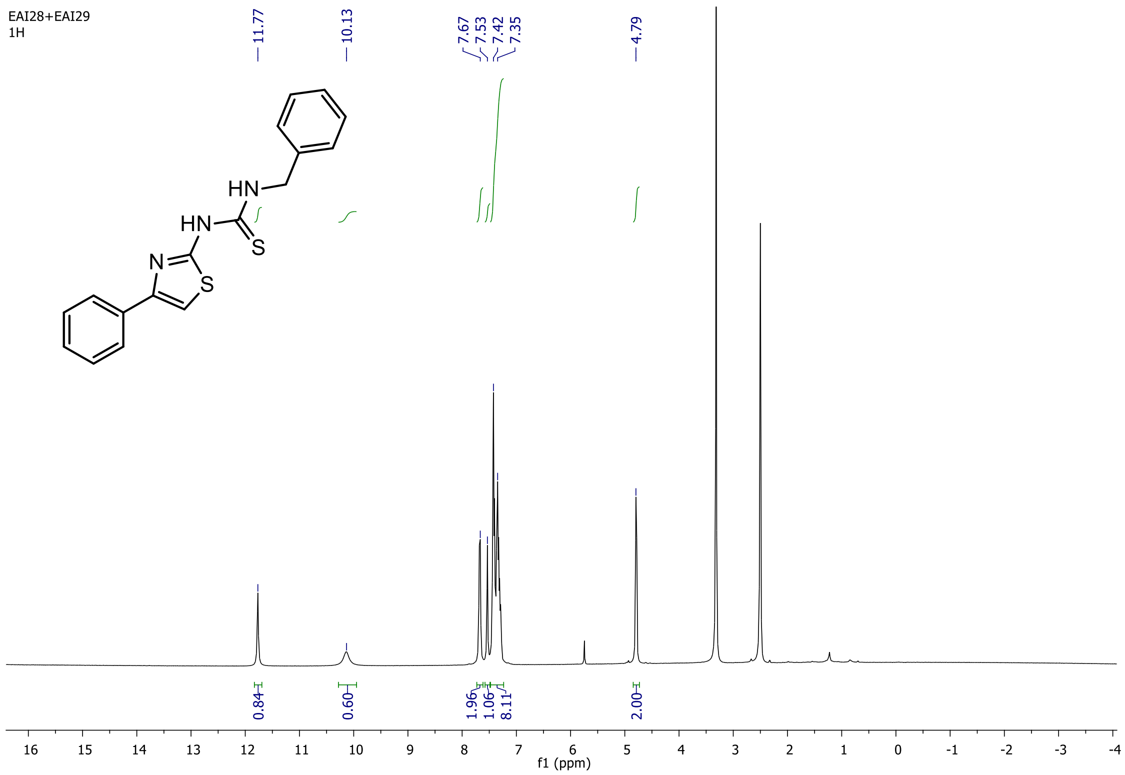

Figure SI_48. ^{1}H NMR of compound **49** (1-benzyl-3-(4-phenylthiazol-2-yl)thiourea) in DMSO

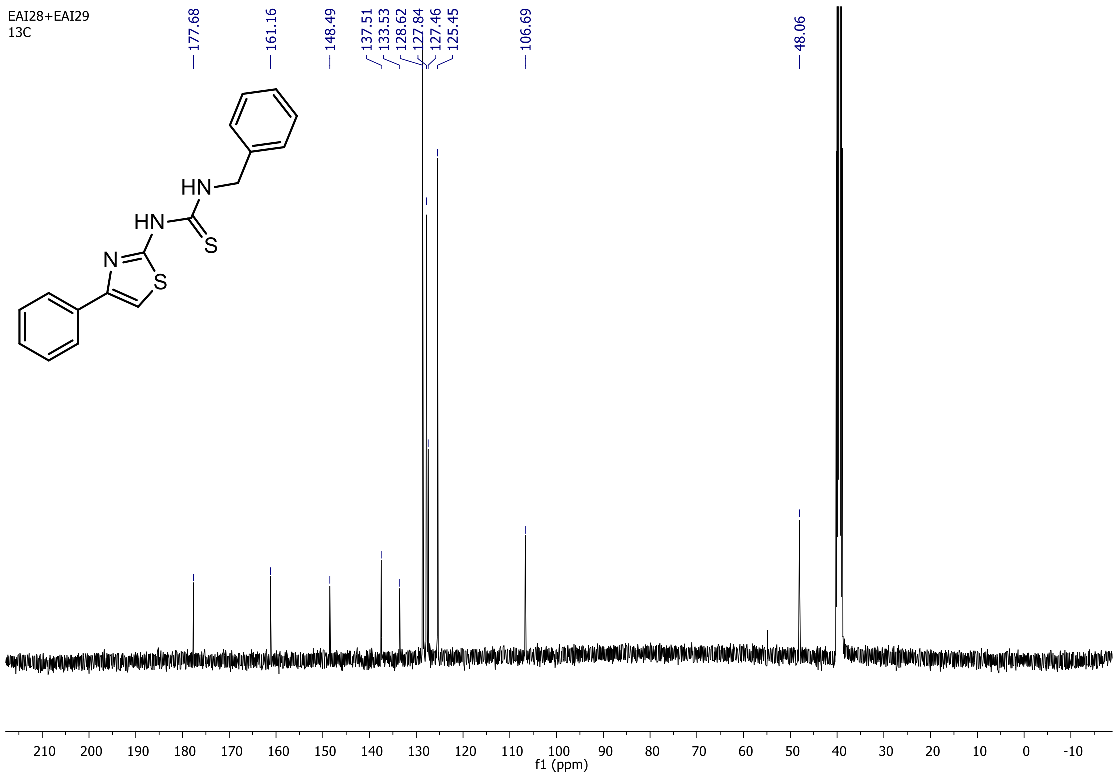

Figure SI_49. ^{13}C NMR of compound **49** (1-benzyl-3-(4-phenylthiazol-2-yl)thiourea) in DMSO

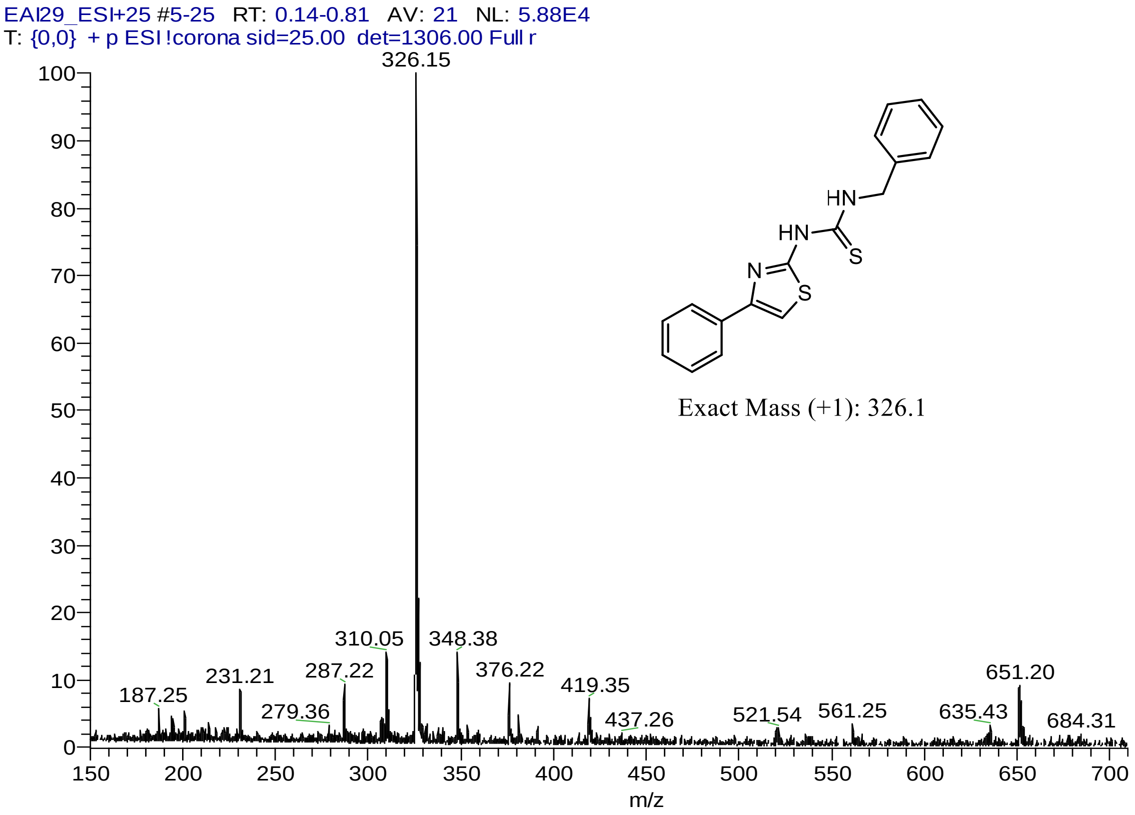

Figure SI_50. ESI MS of compound **49** (1-benzyl-3-(4-phenylthiazol-2-yl)thiourea)

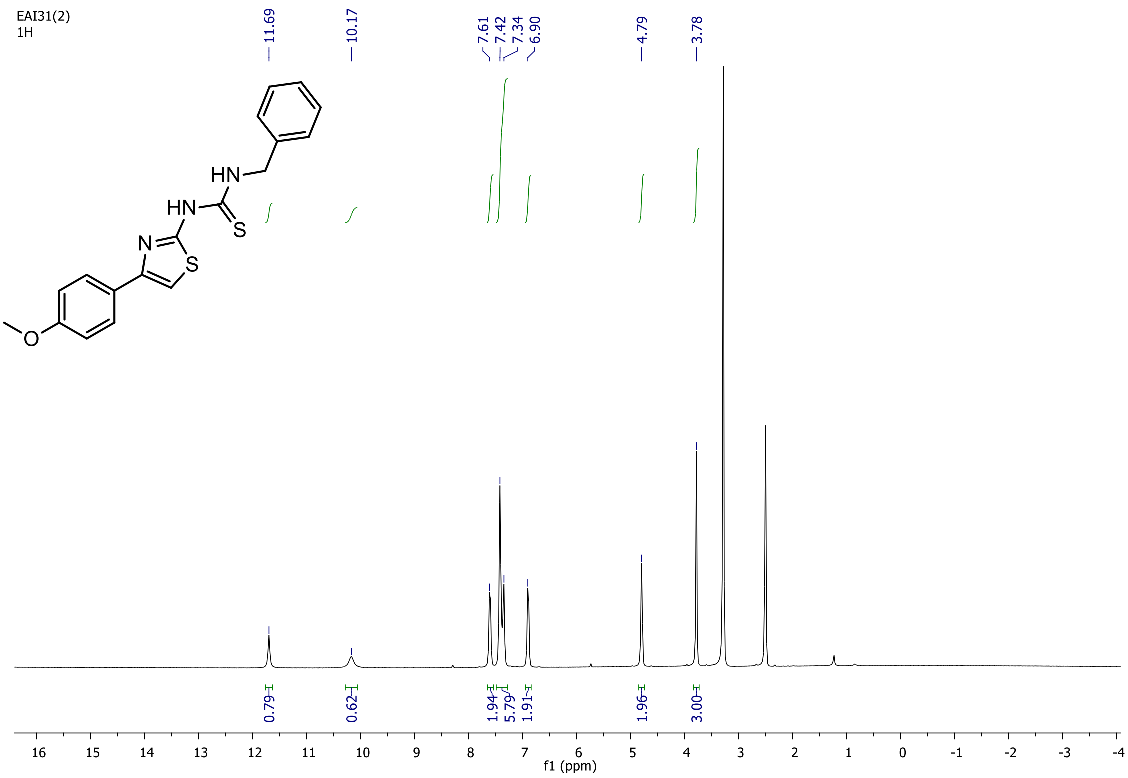

Figure SI_51. ^{1}H NMR of compound **50** (1-benzyl-3-(4-(4-methoxyphenyl)thiazol-2-yl)thiourea) in DMSO

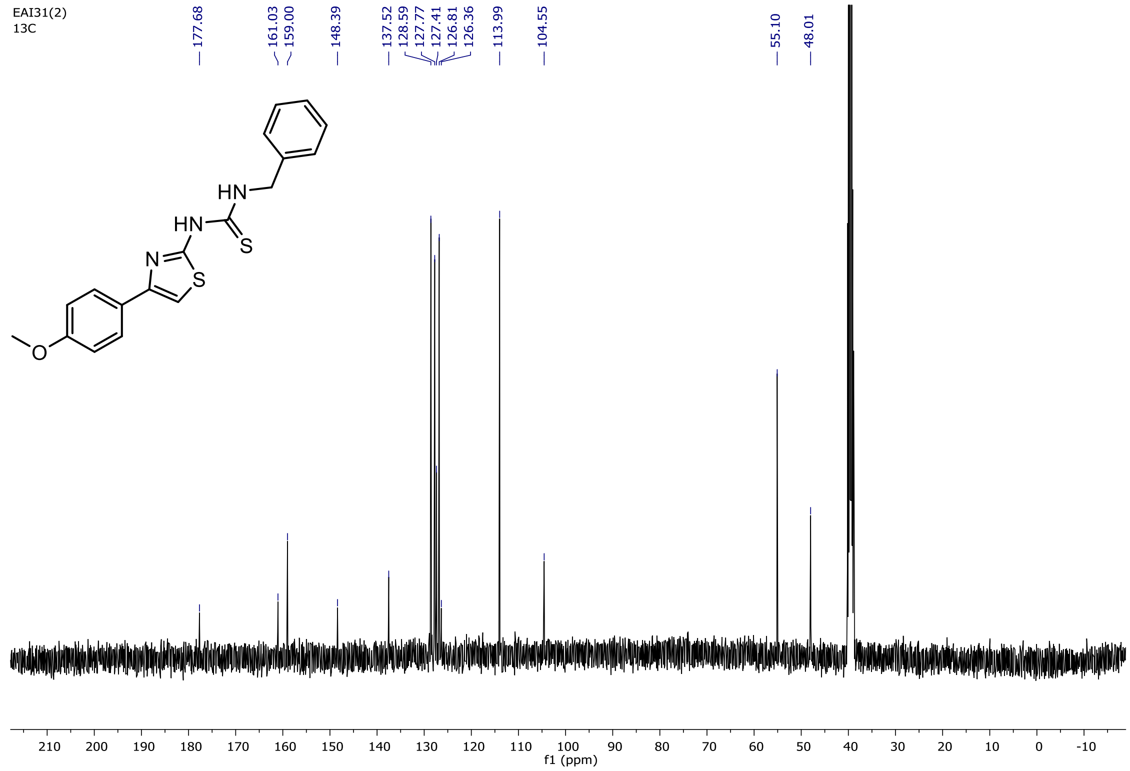

Figure SI_52. ^{13}C NMR of compound **50** (1-benzyl-3-(4-(4-methoxyphenyl)thiazol-2-yl)thiourea) in DMSO

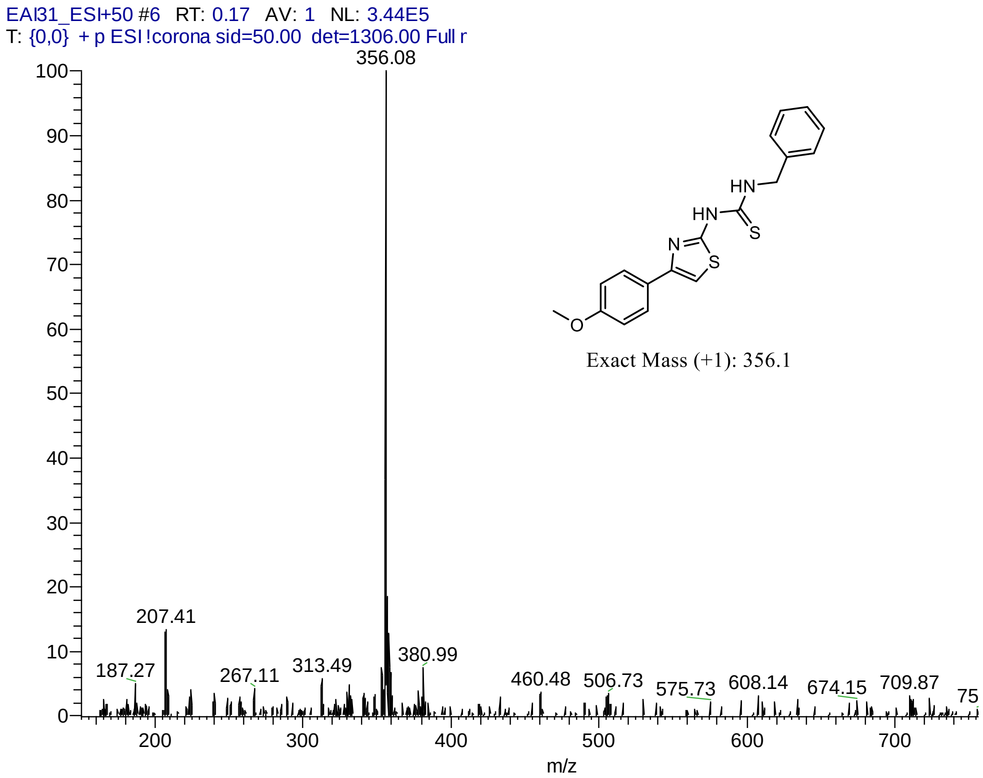

Figure SI_53. ESI MS of compound **50** (1-benzyl-3-(4-(4-methoxyphenyl)thiazol-2-yl)thiourea)

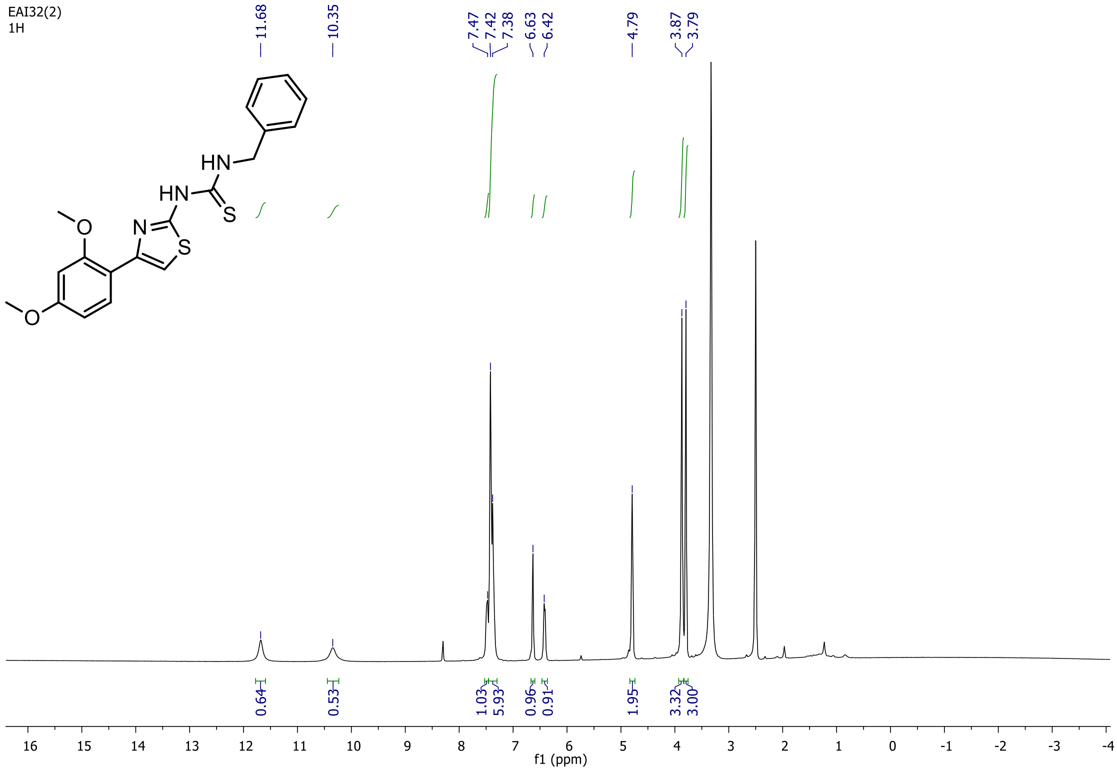

Figure SI_54. ^{1}H NMR of compound **51** (1-benzyl-3-(4-(2,4-dimethoxyphenyl)thiazol-2-yl)thiourea) in DMSO

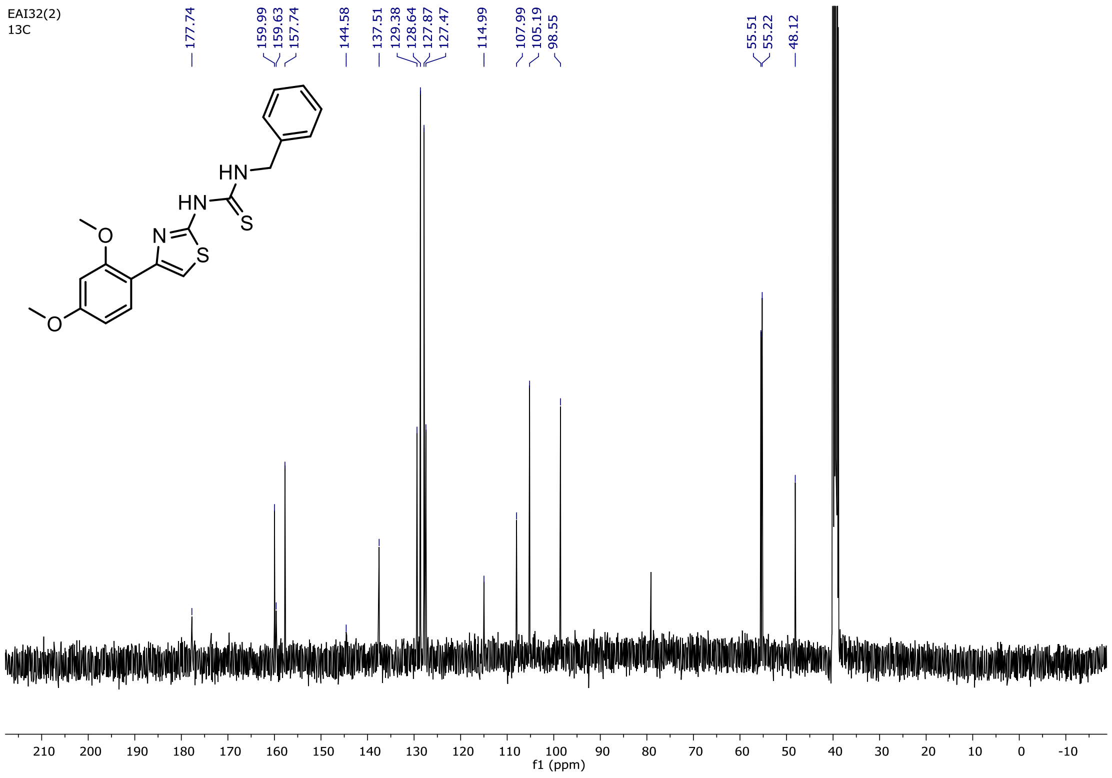

Figure SI_55. ^{13}C NMR of compound **51** (1-benzyl-3-(4-(2,4-dimethoxyphenyl)thiazol-2-yl)thiourea) in DMSO

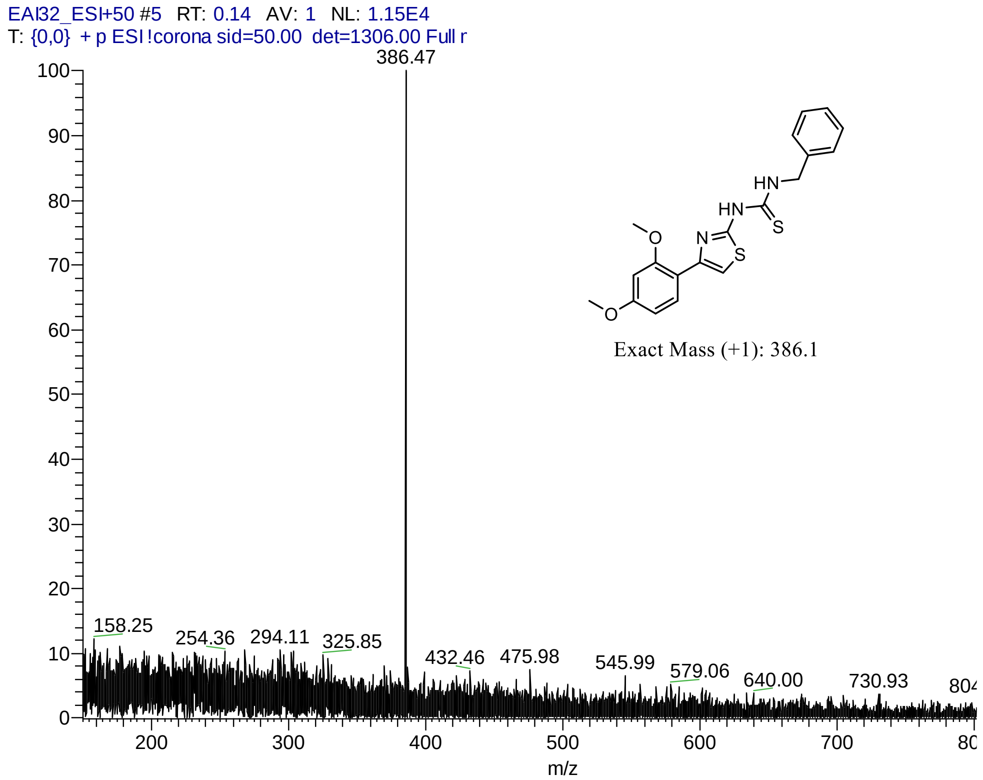

Figure SI_56. ESI MS of compound **51** (1-benzyl-3-(4-(2,4-dimethoxyphenyl)thiazol-2-yl)thiourea)

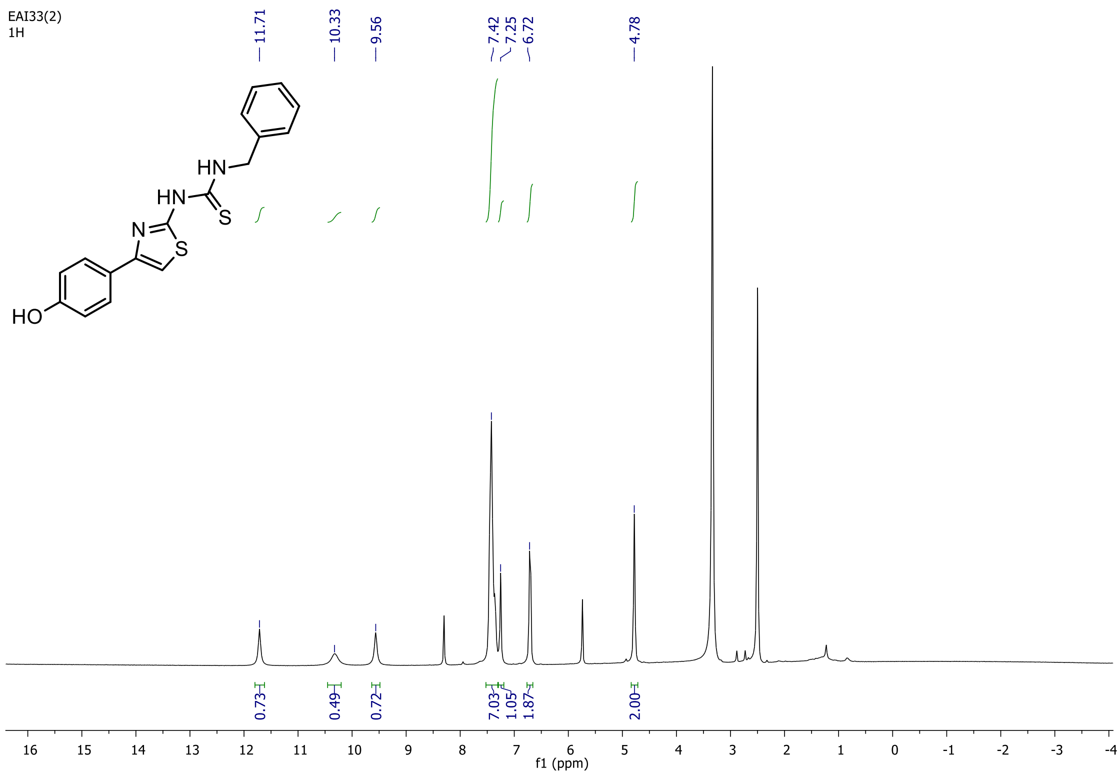

Figure SI_57. ^{1}H NMR of compound **52** (1-benzyl-3-(4-(4-hydroxyphenyl)thiazol-2-yl)thiourea) in DMSO

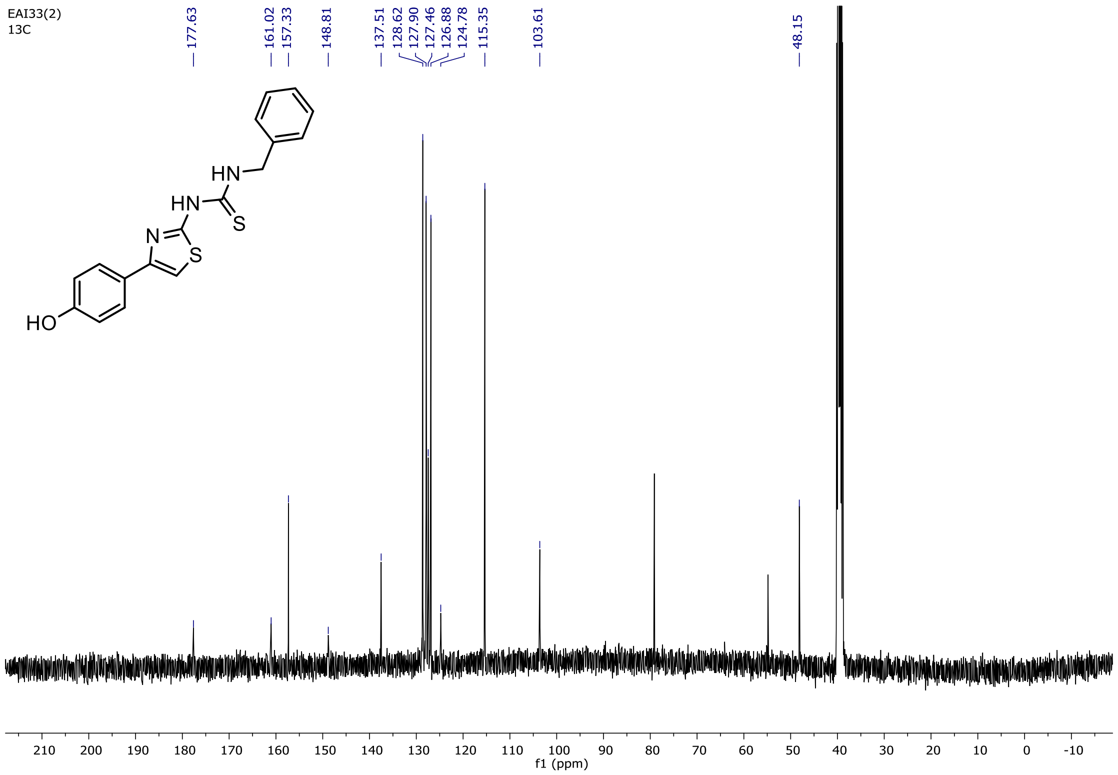

Figure SI_58. ^{13}C NMR of compound **52** (1-benzyl-3-(4-(4-hydroxyphenyl)thiazol-2-yl)thiourea) in DMSO

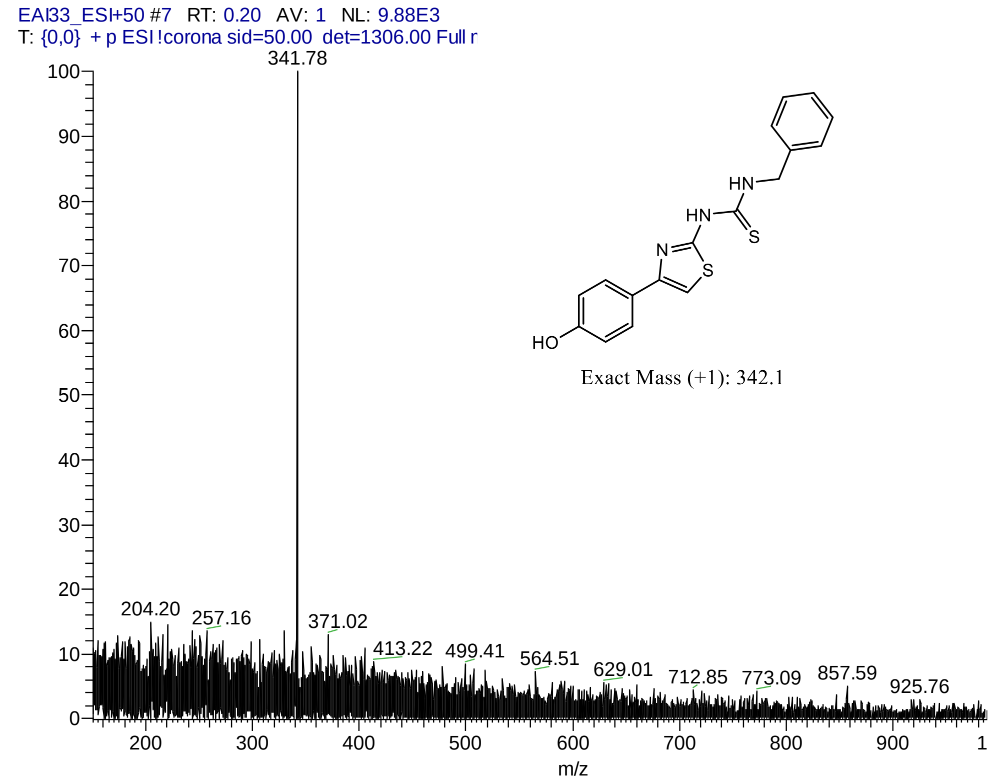

Figure SI_59. ESI MS of compound **52** (1-benzyl-3-(4-(4-hydroxyphenyl)thiazol-2-yl)thiourea)

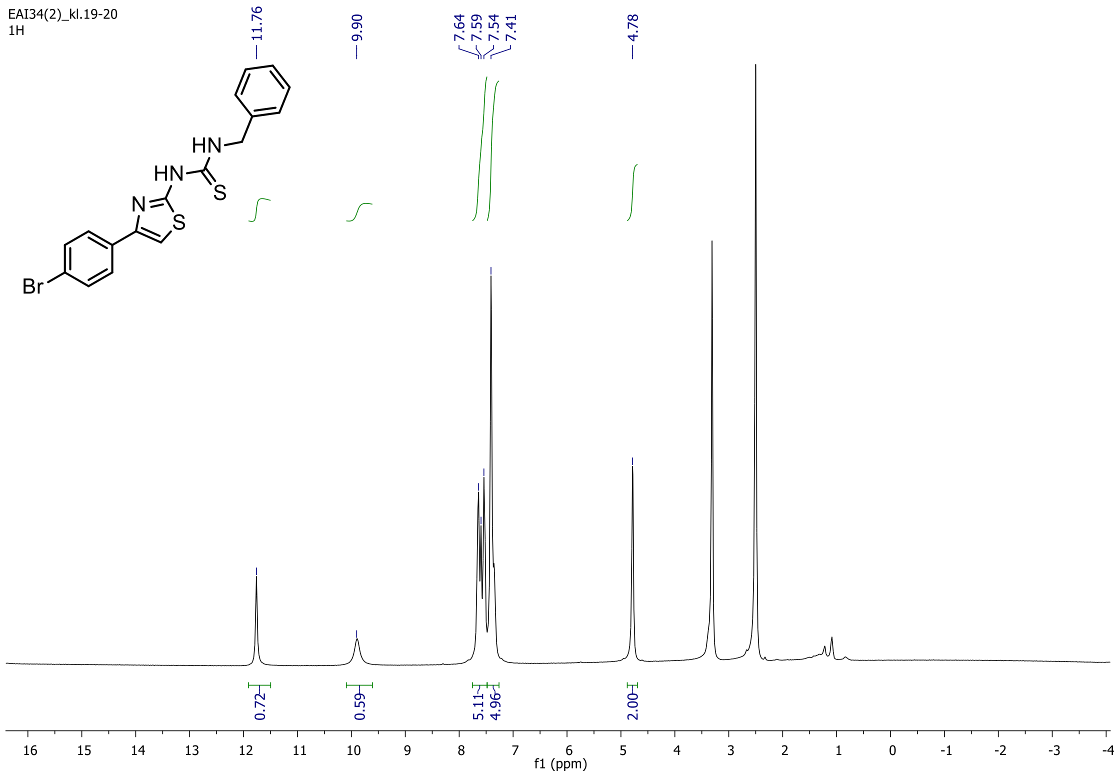

Figure SI_60. ^{1}H NMR of compound **53** (1-benzyl-3-(4-(4-bromophenyl)thiazol-2-yl)thiourea) in DMSO

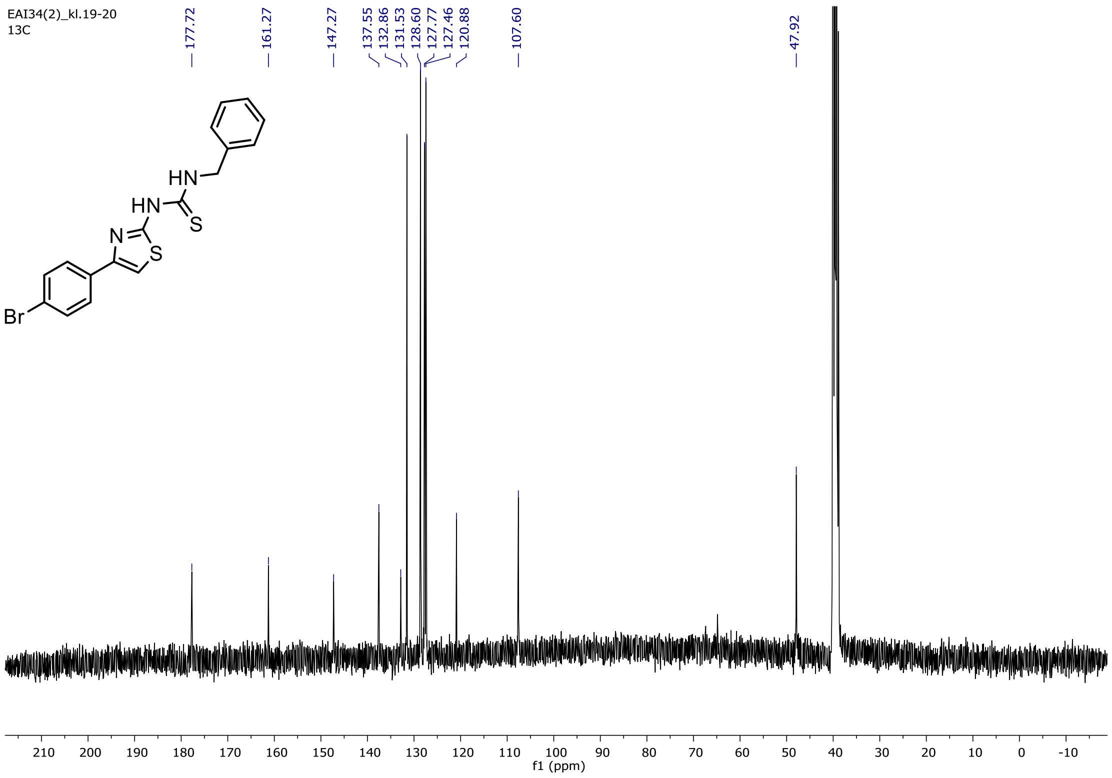

Figure SI_61. ^{13}C NMR of compound **53** (1-benzyl-3-(4-(4-bromophenyl)thiazol-2-yl)thiourea) in DMSO

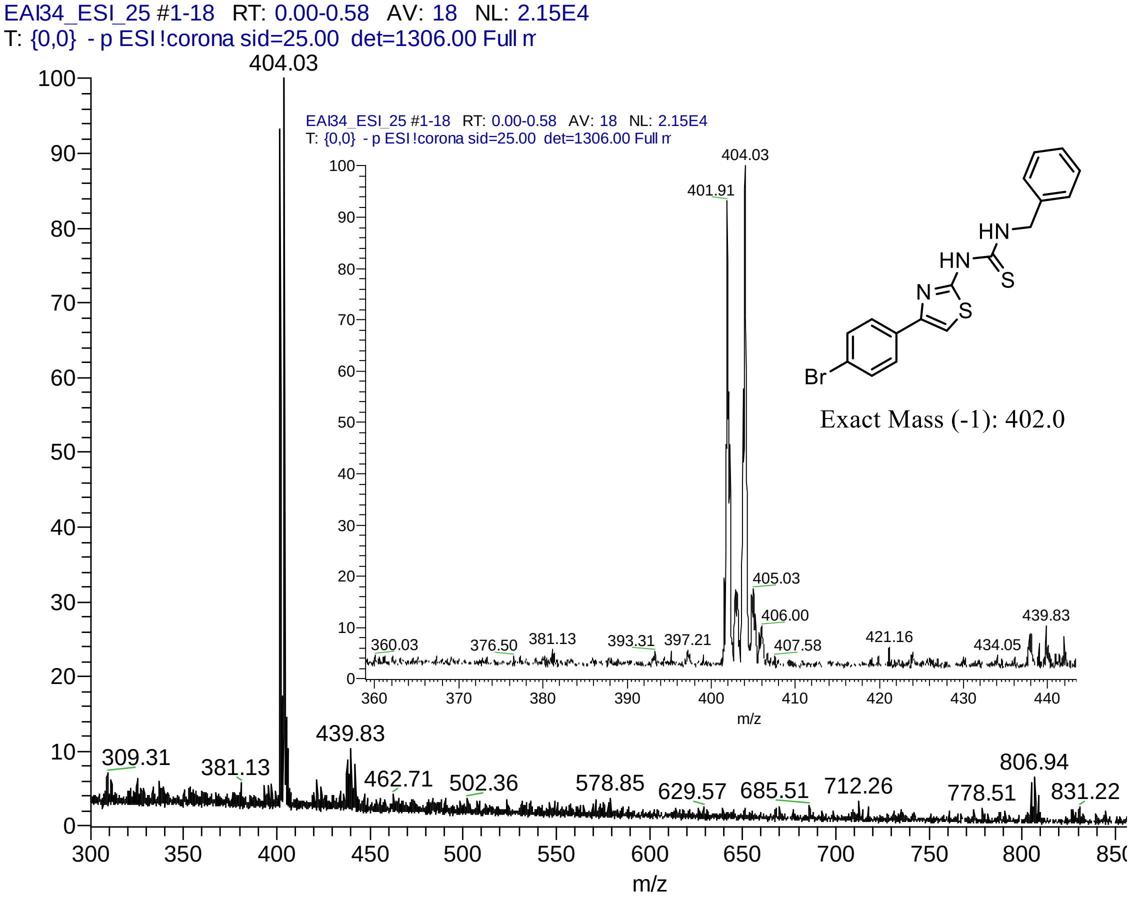

Figure SI_62. ESI MS of compound **53** (1-benzyl-3-(4-(4-bromophenyl)thiazol-2-yl)thiourea)

Table SI_1. Kinetic characteristics of studied thiourea derivatives.

Entry	
**15**	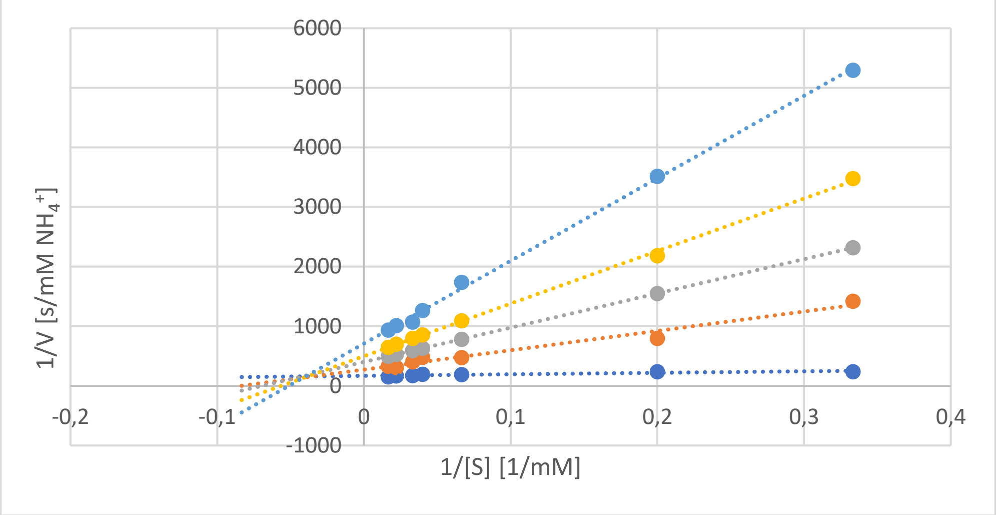
**16**	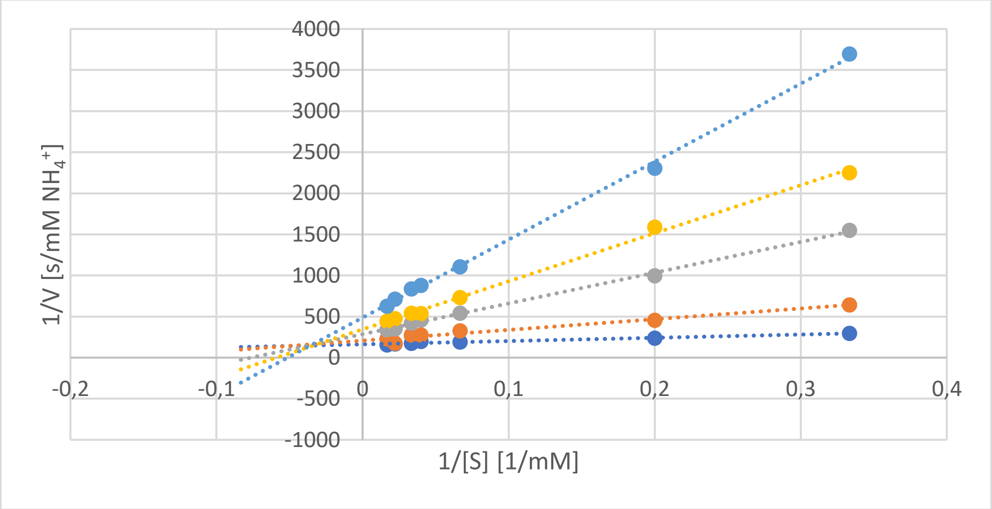
**17**	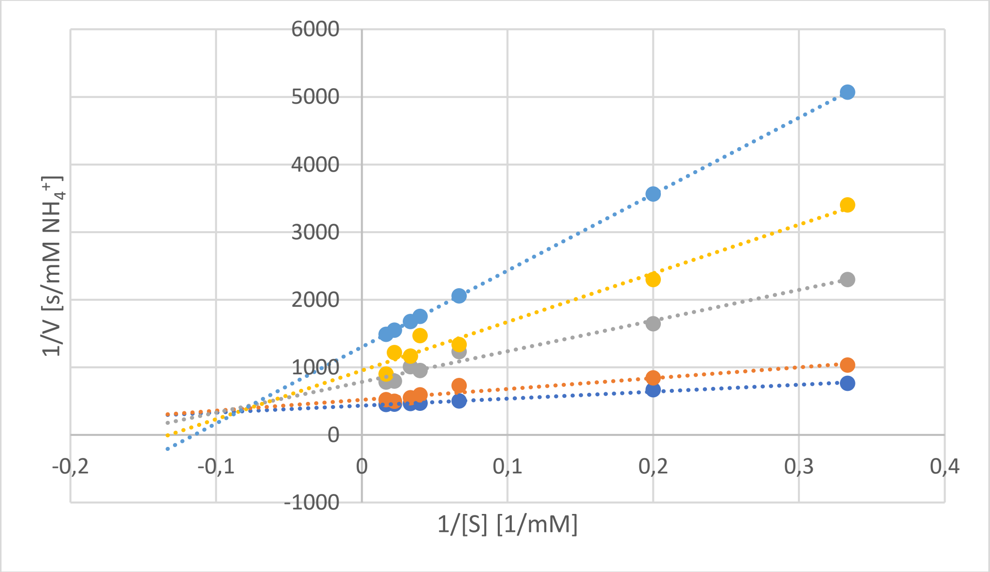

18

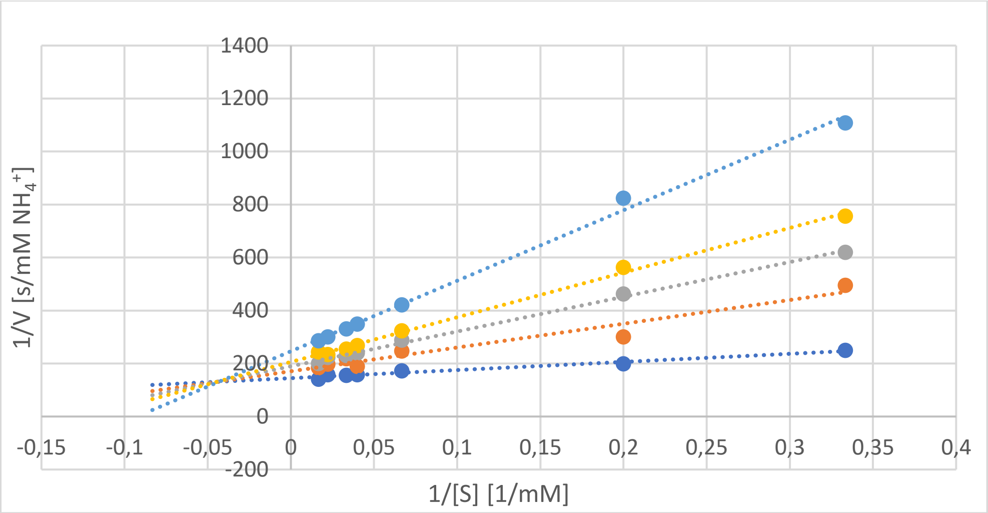
1400
1200
1000
800
600
400
200
0
-200
-0,15
-0,1
-0,05
0
0,05
0,1
0,15
0,2
0,25
0,3
0,35
0,4
1/V [s/mM NH4+]
1/[S] [1/mM]

19

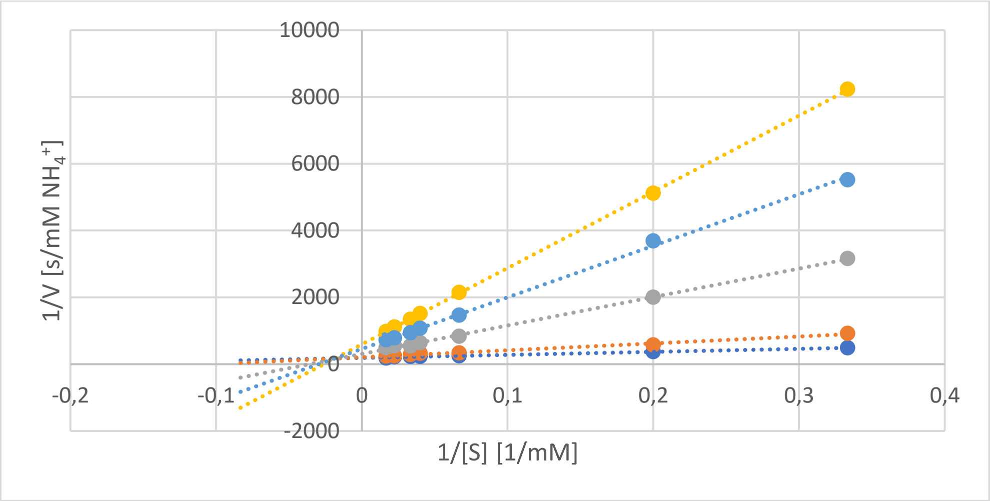
10000
8000
6000
4000
2000
0
-2000
-0,2
-0,1
0
0,1
0,2
0,3
0,4
1/V [s/mM NH4+]
1/[S] [1/mM]

20

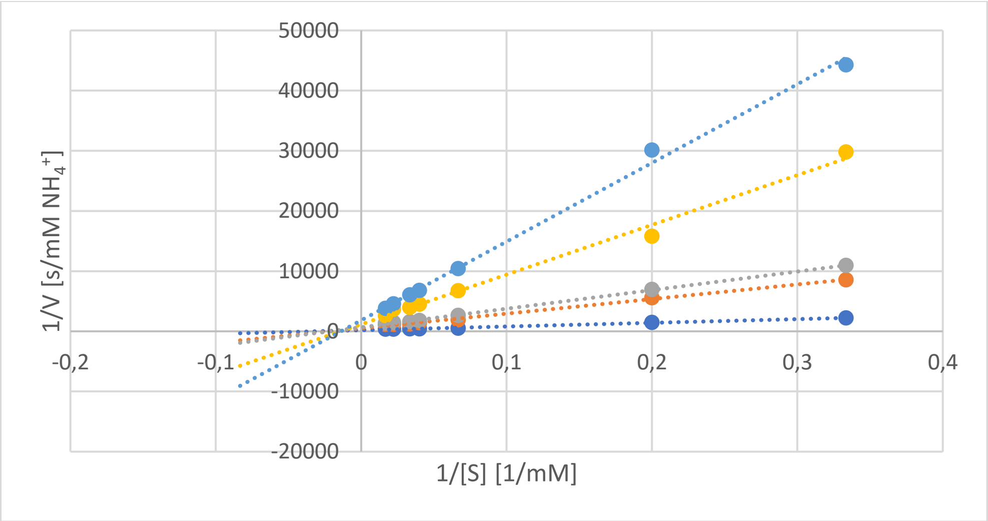
50000
40000
30000
20000
10000
0
-10000
-20000
-0,2
-0,1
0
0,1
0,2
0,3
0,4
1/V [s/mM NH4+]
1/[S] [1/mM]

21

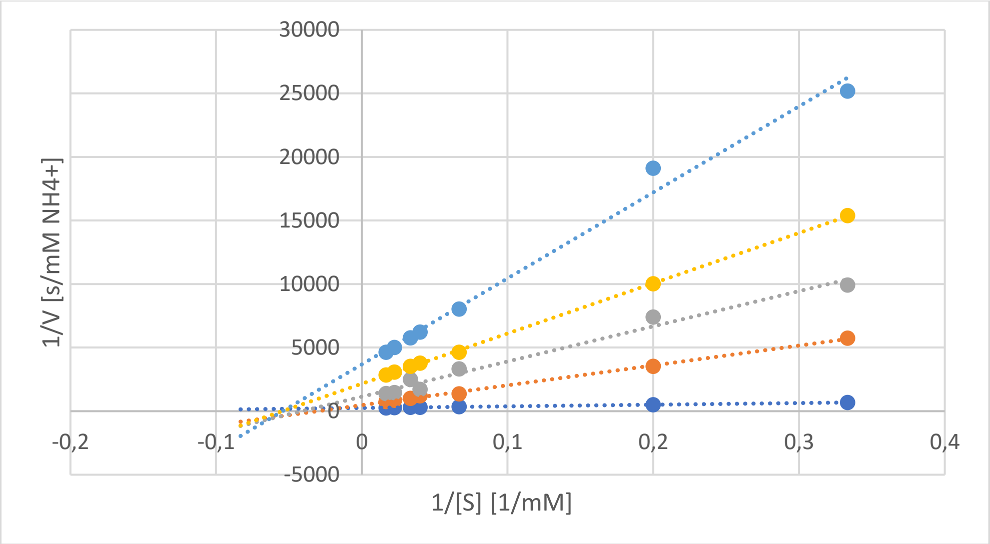

30000
25000
20000
15000
10000
5000
0
-5000
-0,2
-0,1
0
0,1
0,2
0,3
0,4
1/V [s/mM NH4+]
1/[S] [1/mM]

22

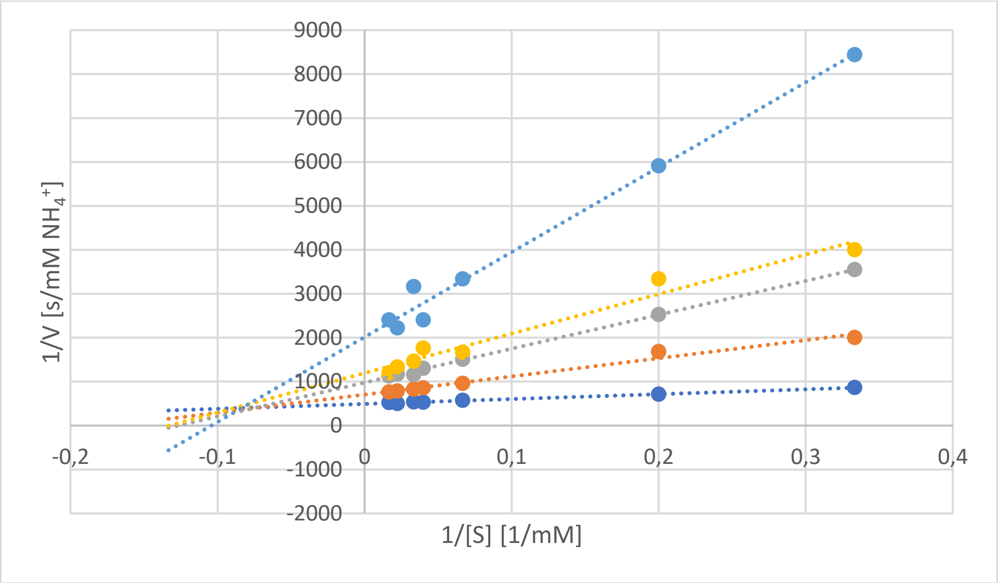

9000
8000
7000
6000
5000
4000
3000
2000
1000
0
-1000
-2000
-0,2
-0,1
0
0,1
0,2
0,3
0,4
1/V [s/mM NH_4^+]
1/[S] [1/mM]

23

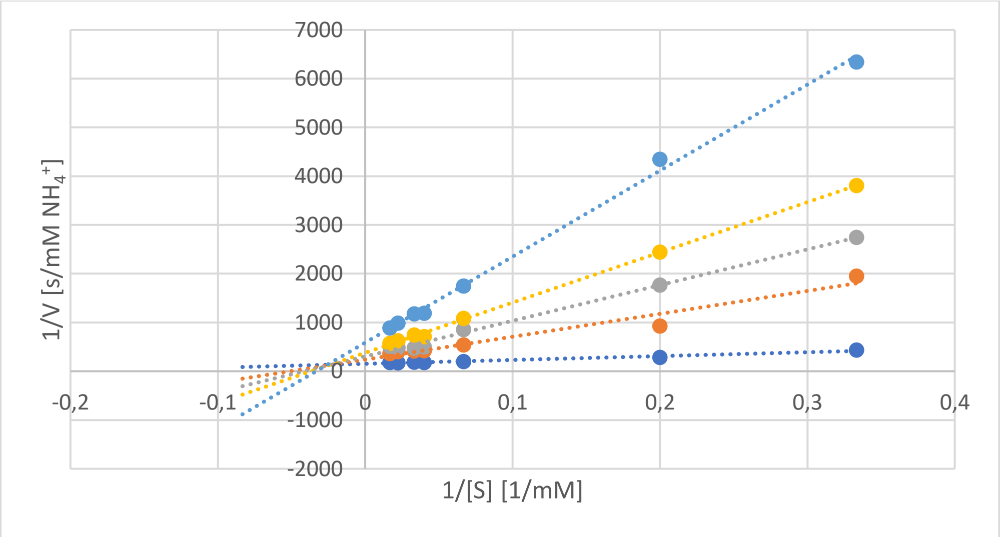

7000
6000
5000
4000
3000
2000
1000
0
-1000
-2000
-0,2
-0,1
0
0,1
0,2
0,3
0,4
1/V [s/mM NH_4^+]
1/[S] [1/mM]

24

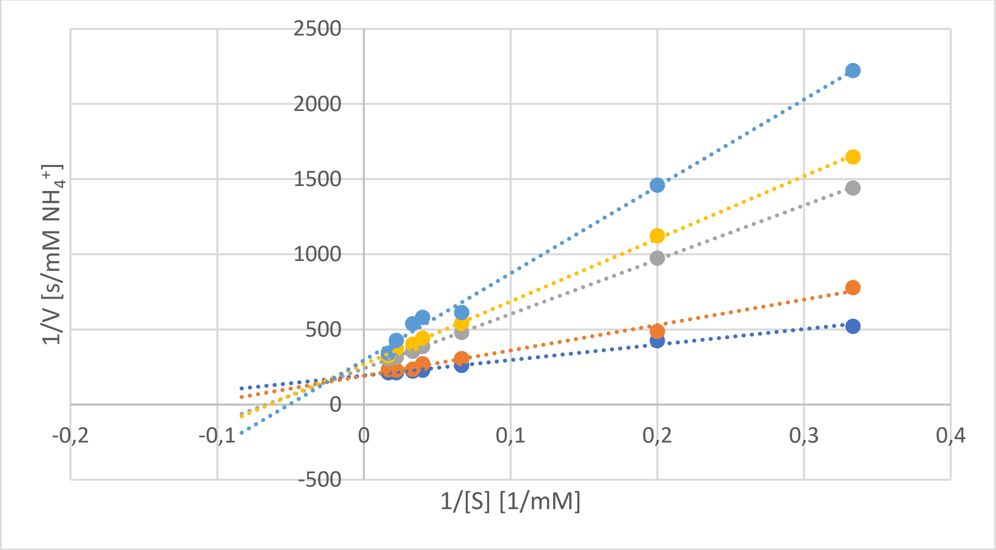
2500
2000
1500
1000
500
0
-500
-0,2
-0,1
0
0,1
0,2
0,3
0,4
1/V [s/mM NH_4^+]
1/[S] [1/mM]

25

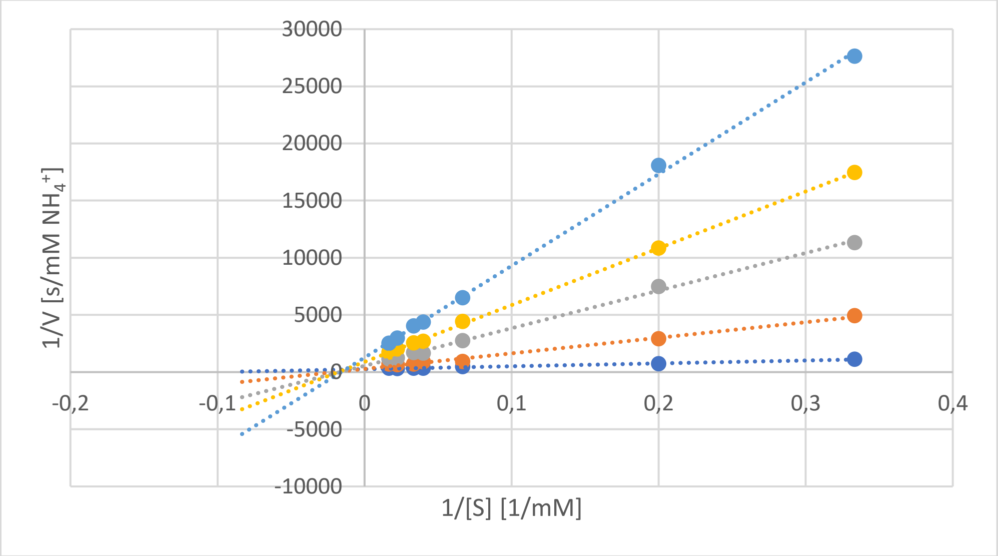
30000
25000
20000
15000
10000
5000
0
-5000
-10000
-0,2
-0,1
0
0,1
0,2
0,3
0,4
1/V [s/mM NH_4^+]
1/[S] [1/mM]

26

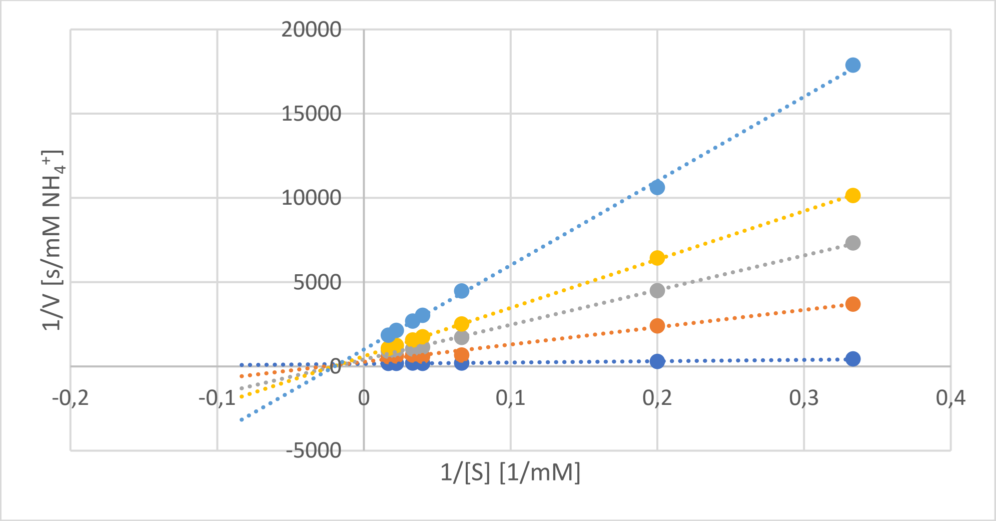
20000
15000
10000
5000
0
-5000
-0,2
-0,1
0
0,1
0,2
0,3
0,4
1/V [s/mM NH_4^+]
1/[S] [1/mM]

27

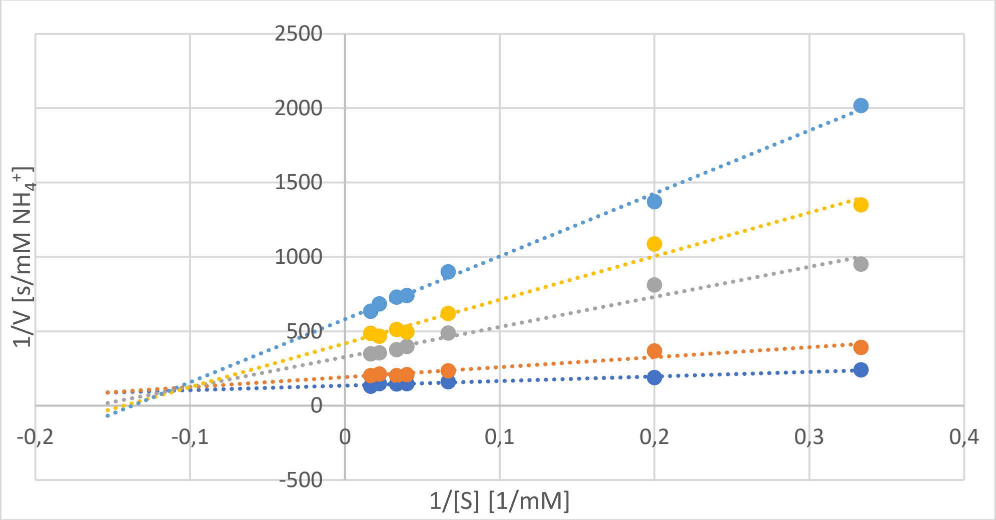

2500
2000
1500
1000
500
0
-500
-0,2
-0,1
0
0,1
0,2
0,3
0,4
1/V [s/mM NH$_4^+$]
1/[S] [1/mM]

28

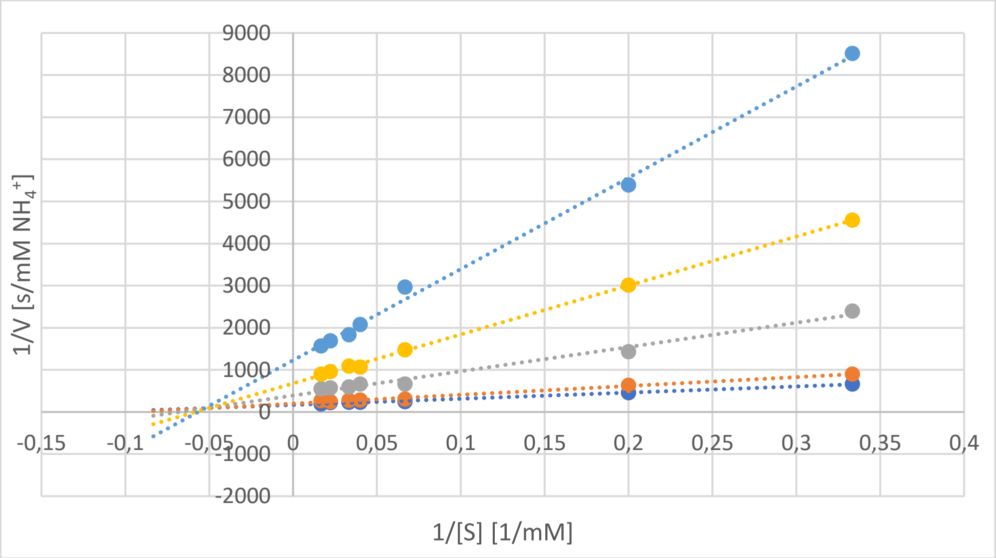

9000
8000
7000
6000
5000
4000
3000
2000
1000
0
-1000
-2000
-0,15
-0,1
-0,05
0
0,05
0,1
0,15
0,2
0,25
0,3
0,35
0,4
1/V [s/mM NH$_4^+$]
1/[S] [1/mM]

29

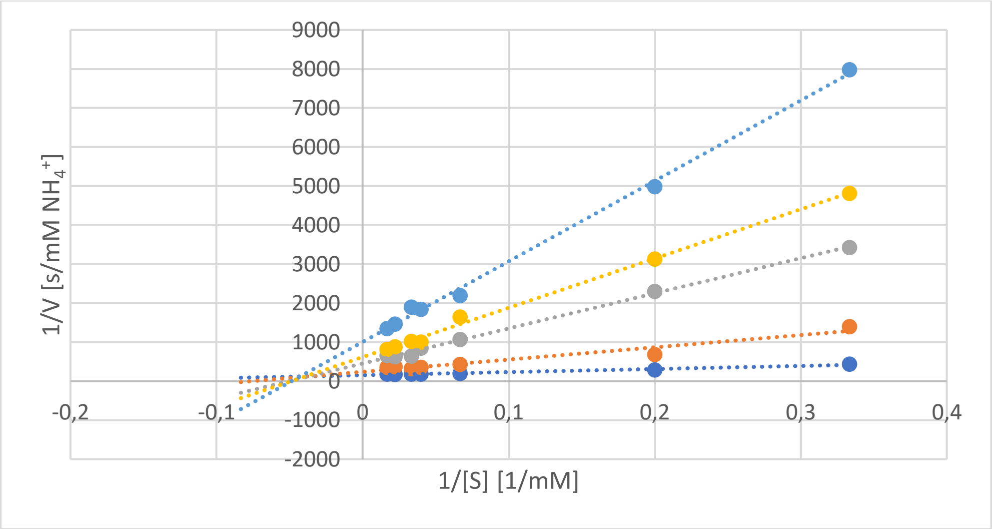

9000
8000
7000
6000
5000
4000
3000
2000
1000
0
-1000
-2000
-0,2
-0,1
0
0,1
0,2
0,3
0,4
1/V [s/mM NH$_4^+$]
1/[S] [1/mM]

30

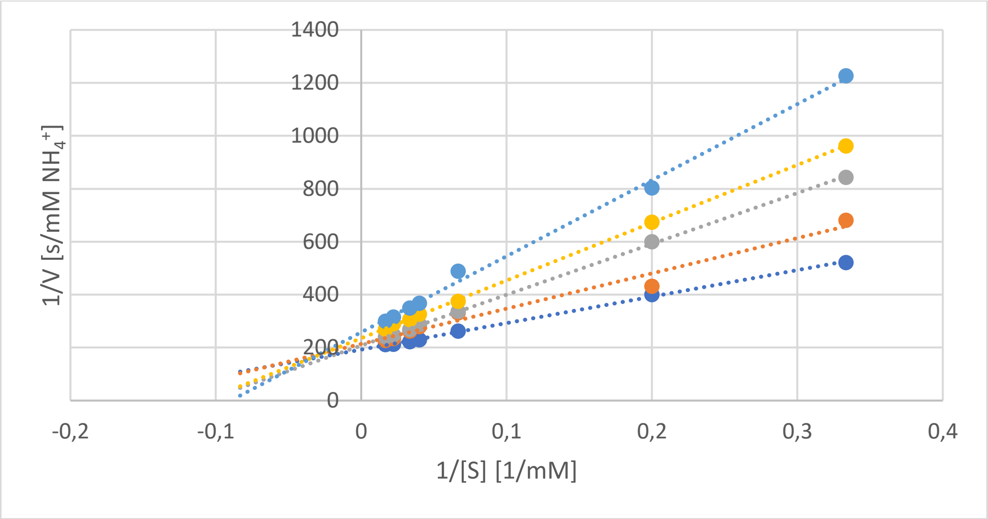
1400
1200
1000
800
600
400
200
0
-0,2
-0,1
0
0,1
0,2
0,3
0,4
1/V [s/mM NH4+]
1/[S] [1/mM]

31

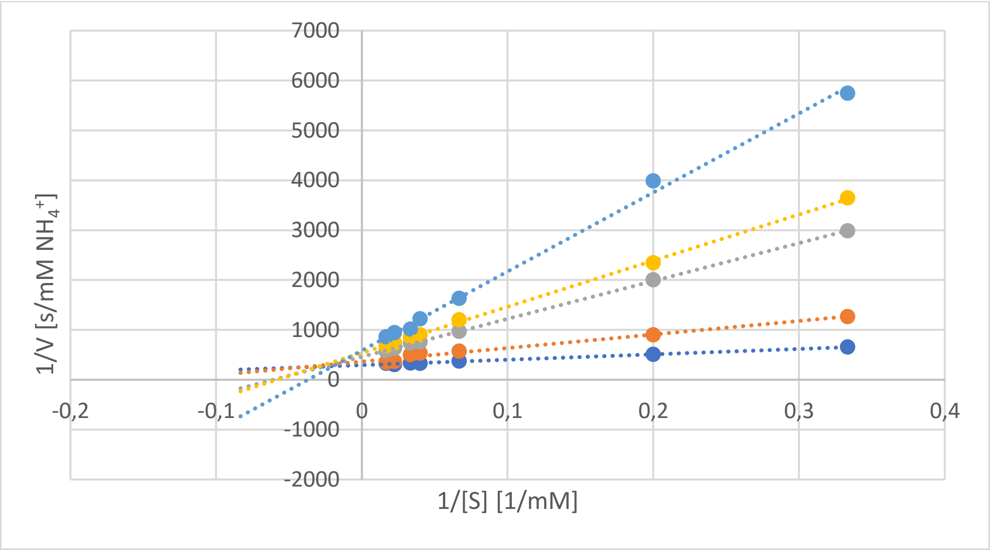
7000
6000
5000
4000
3000
2000
1000
0
-1000
-2000
-0,2
-0,1
0
0,1
0,2
0,3
0,4
1/V [s/mM NH4+]
1/[S] [1/mM]

49

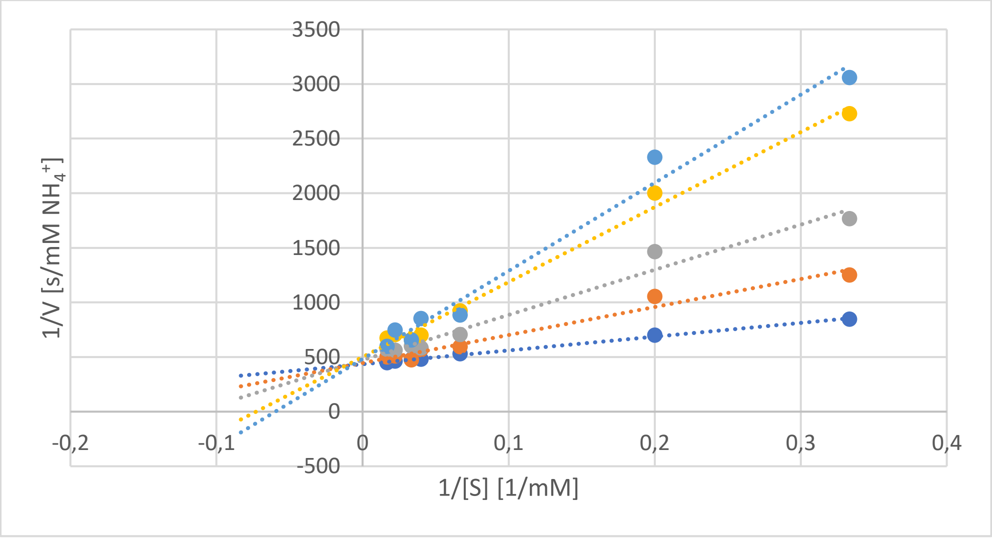
3500
3000
2500
2000
1500
1000
500
0
-500
-0,2
-0,1
0
0,1
0,2
0,3
0,4
1/V [s/mM NH4+]
1/[S] [1/mM]

50

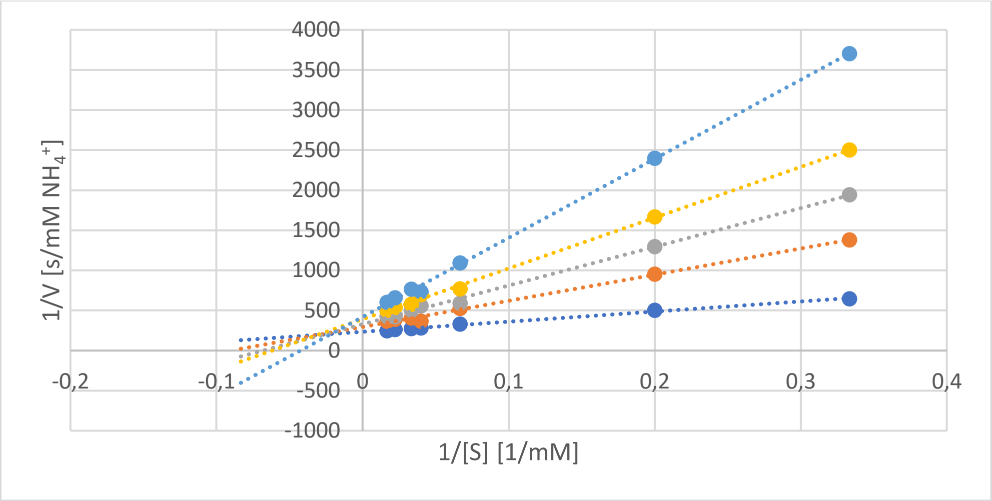

4000
3500
3000
2500
2000
1500
1000
500
0
-500
-1000
-0,2
-0,1
0
0,1
0,2
0,3
0,4
1/V [s/mM NH4+]
1/[S] [1/mM]

51

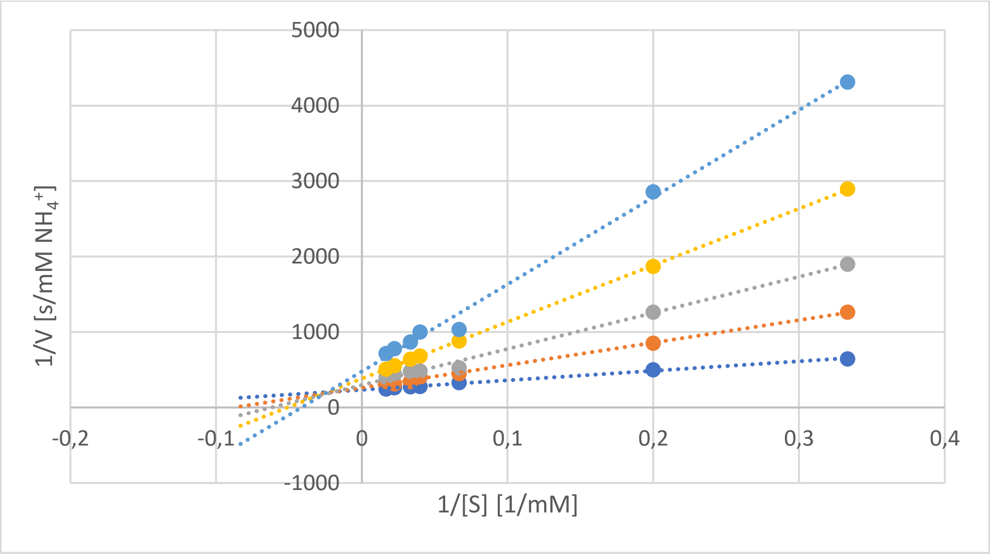

5000
4000
3000
2000
1000
0
-1000
-0,2
-0,1
0
0,1
0,2
0,3
0,4
1/V [s/mM NH4+]
1/[S] [1/mM]

52

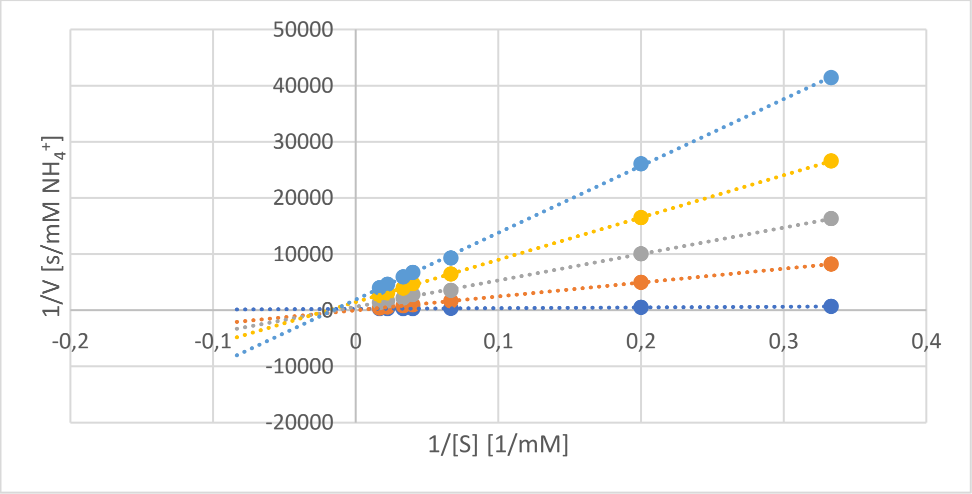

50000
40000
30000
20000
10000
0
-10000
-20000
-0,2
-0,1
0
0,1
0,2
0,3
0,4
1/V [s/mM NH4+]
1/[S] [1/mM]

53

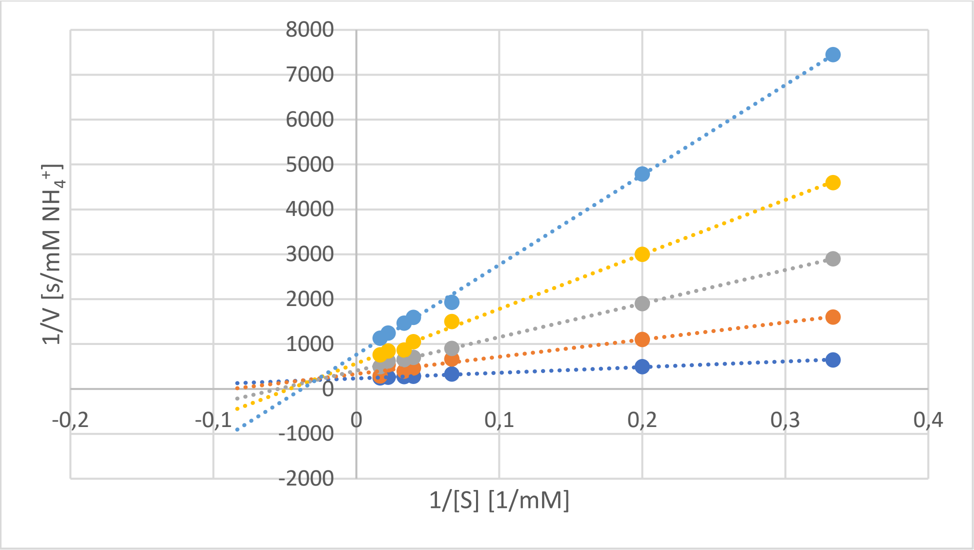

8000
7000
6000
5000
4000
3000
2000
1000
0
-1000
-2000
-0,2
-0,1
0
0,1
0,2
0,3
0,4
1/V [s/mM NH_4^+]
1/[S] [1/mM]

32

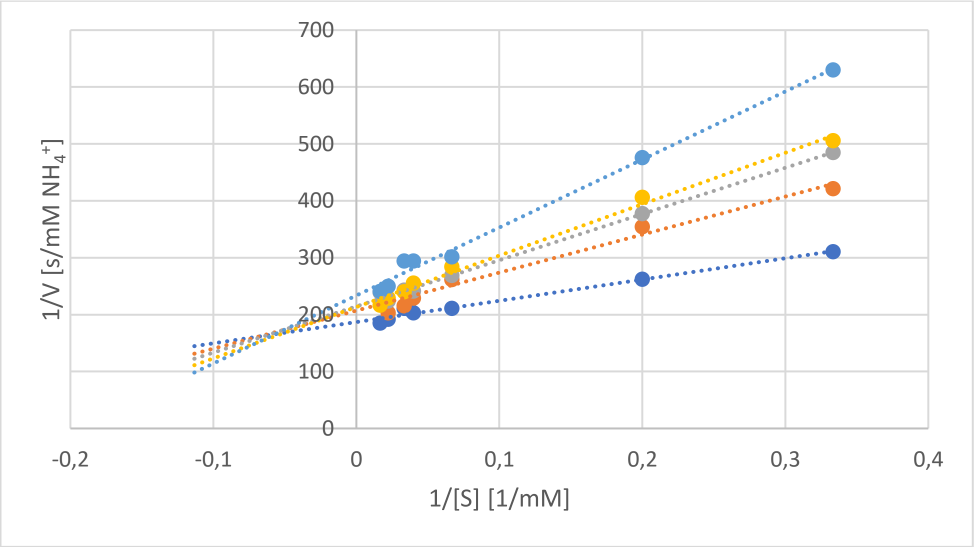

700
600
500
400
300
200
100
0
-0,2
-0,1
0
0,1
0,2
0,3
0,4
1/V [s/mM NH_4^+]
1/[S] [1/mM]

33

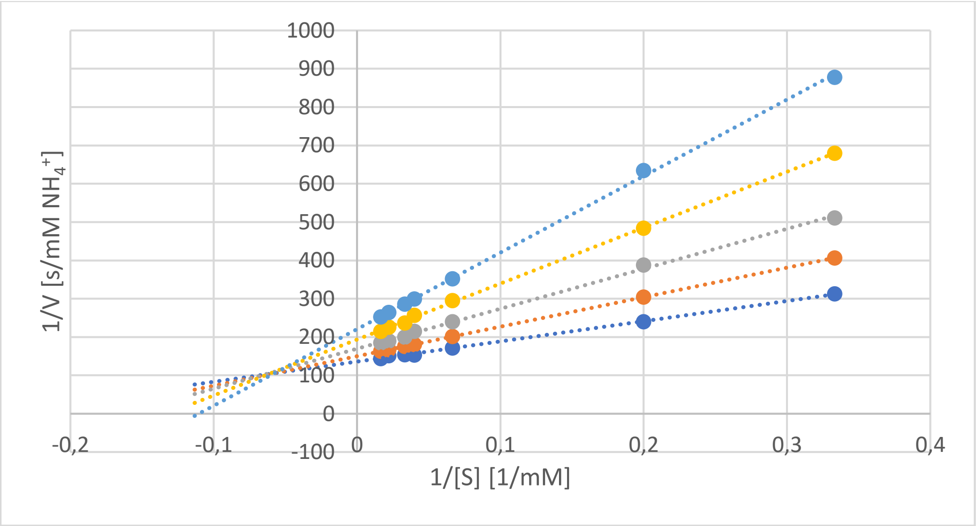

1000
900
800
700
600
500
400
300
200
100
0
-100
-0,2
-0,1
0
0,1
0,2
0,3
0,4
1/V [s/mM NH_4^+]
1/[S] [1/mM]

34

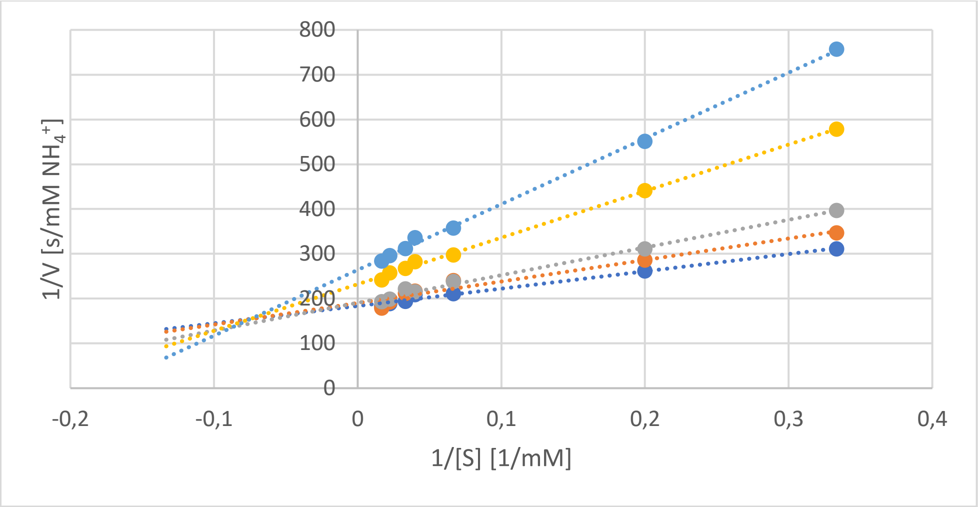
800
700
600
500
400
300
200
100
0
-0,2
-0,1
0
0,1
0,2
0,3
0,4
1/V [s/mM NH4+]
1/[S] [1/mM]

35

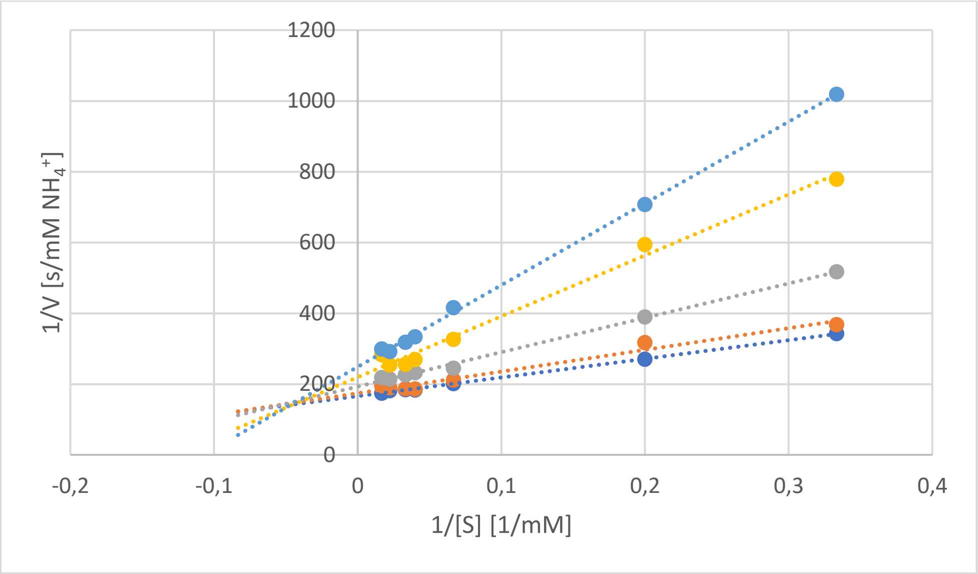
1200
1000
800
600
400
200
0
-0,2
-0,1
0
0,1
0,2
0,3
0,4
1/V [s/mM NH4+]
1/[S] [1/mM]

36

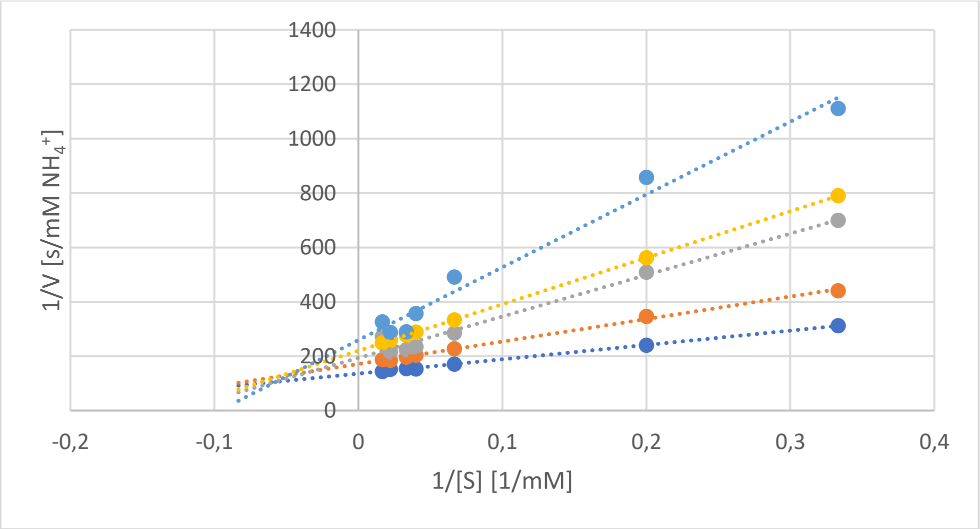
1400
1200
1000
800
600
400
200
0
-0,2
-0,1
0
0,1
0,2
0,3
0,4
1/V [s/mM NH4+]
1/[S] [1/mM]

37

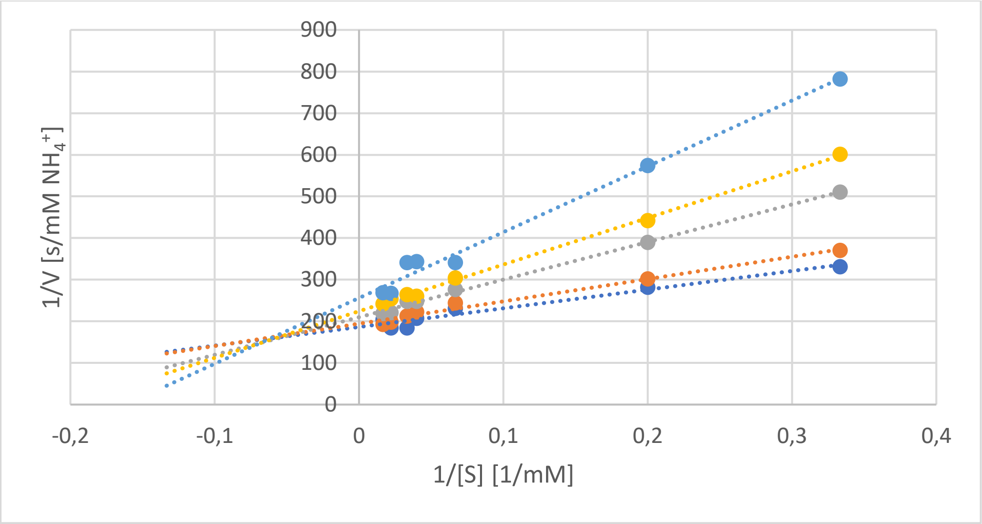
900
800
700
600
500
400
300
200
100
0
-0,2
-0,1
0
0,1
0,2
0,3
0,4
1/V [s/mM NH_4^+]
1/[S] [1/mM]

38

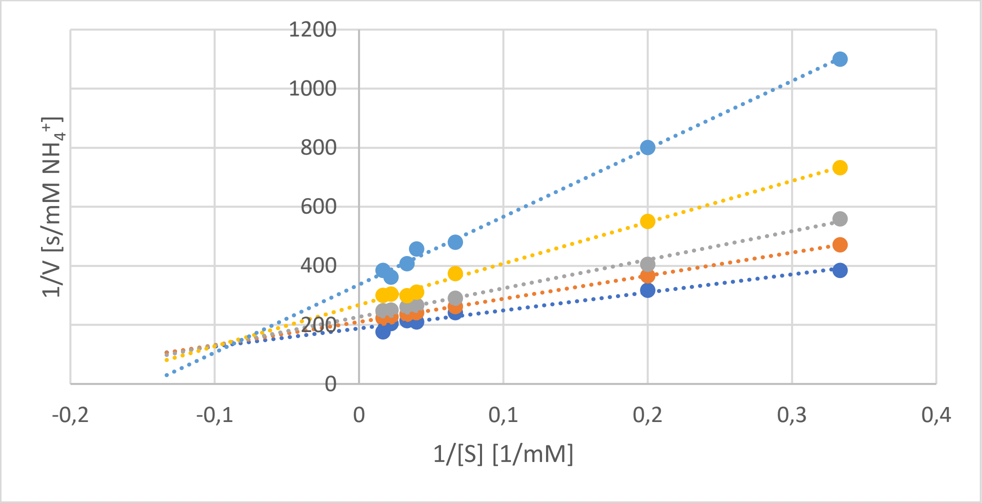
1200
1000
800
600
400
200
0
-0,2
-0,1
0
0,1
0,2
0,3
0,4
1/V [s/mM NH_4^+]
1/[S] [1/mM]

39

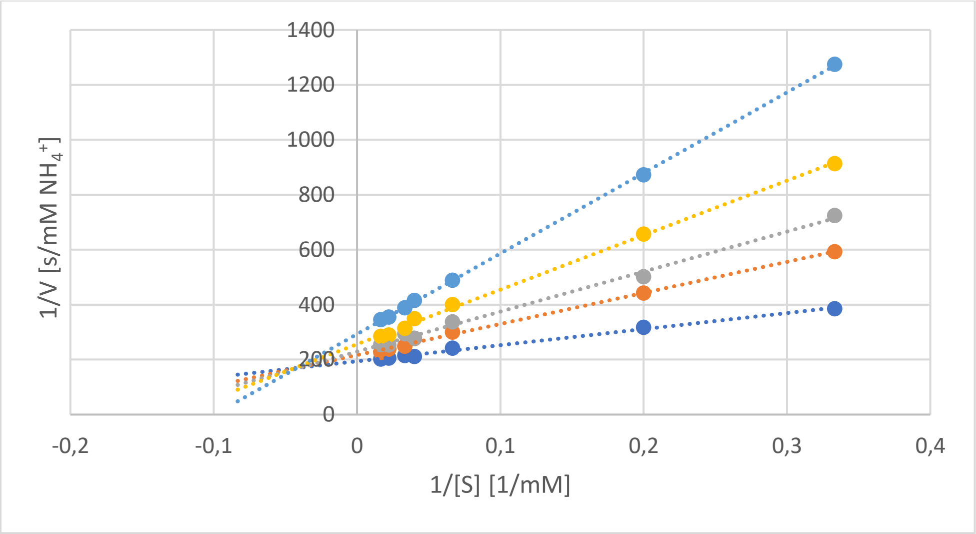
1400
1200
1000
800
600
400
200
0
-0,2
-0,1
0
0,1
0,2
0,3
0,4
1/V [s/mM NH_4^+]
1/[S] [1/mM]

40

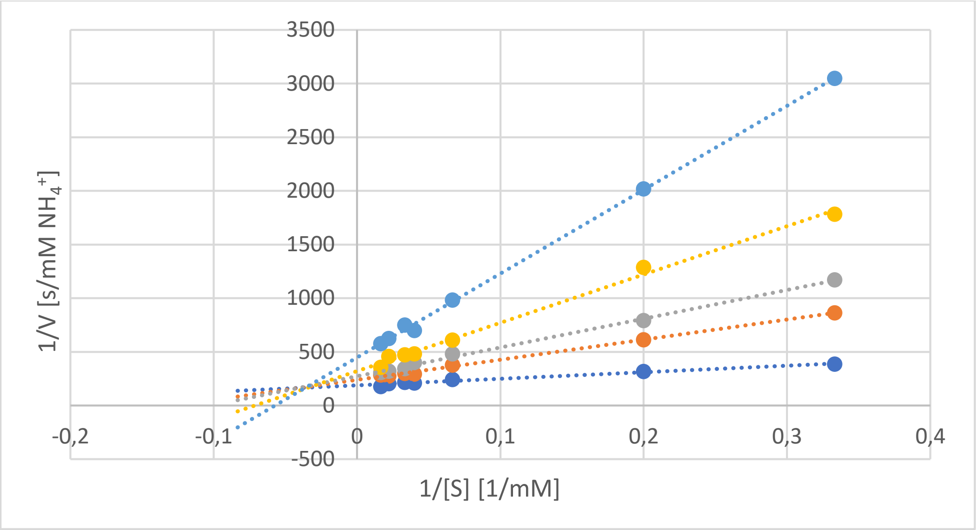

3500
3000
2500
2000
1500
1000
500
0
-500
-0,2
-0,1
0
0,1
0,2
0,3
0,4
1/V [s/mM NH4+]
1/[S] [1/mM]

41

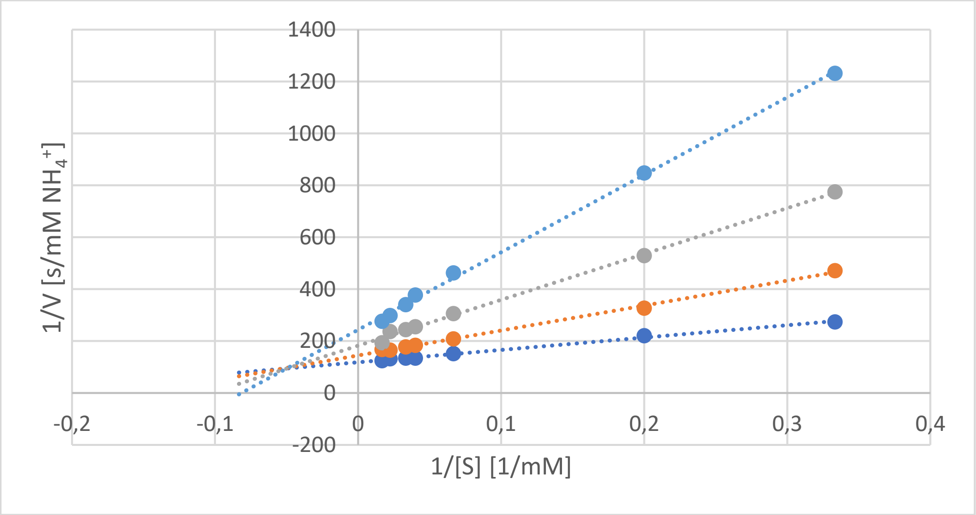

1400
1200
1000
800
600
400
200
0
-200
-0,2
-0,1
0
0,1
0,2
0,3
0,4
1/V [s/mM NH4+]
1/[S] [1/mM]
